# Magnetically assisted soft milli-tools for occluded lumen morphology detection

**DOI:** 10.1126/sciadv.adi3979

**Published:** 2023-08-16

**Authors:** Yingbo Yan, Tianlu Wang, Rongjing Zhang, Yilun Liu, Wenqi Hu, Metin Sitti

**Affiliations:** ^1^ Physical Intelligence Department, Max Planck Institute for Intelligent Systems, Stuttgart 70569, Germany.; ^2^ Laboratory for Multiscale Mechanics and Medical Science, SV LAB, School of Aerospace, Xi’an Jiaotong University, Xi’an 710049, China.; ^3^ Department of Information Technology and Electrical Engineering, ETH Zurich, 8092 Zurich, Switzerland.; ^4^ School of Medicine and College of Engineering, Koç University, Istanbul 34450, Turkey.

## Abstract

Methodologies based on intravascular imaging have revolutionized the diagnosis and treatment of endovascular diseases. However, current methods are limited in detecting, i.e., visualizing and crossing, complicated occluded vessels. Therefore, we propose a miniature soft tool comprising a magnet-assisted active deformation segment (ADS) and a fluid drag-driven segment (FDS) to visualize and cross the occlusions with various morphologies. First, via soft-bodied deformation and interaction, the ADS could visualize the structure details of partial occlusions with features as small as 0.5 millimeters. Then, by leveraging the fluidic drag from the pulsatile flow, the FDS could automatically detect an entry point selectively from severe occlusions with complicated microchannels whose diameters are down to 0.2 millimeters. The functions have been validated in both biologically relevant phantoms and organs ex vivo. This soft tool could help enhance the efficacy of minimally invasive medicine for the diagnosis and treatment of occlusions in various circulatory systems.

## Teaser

Active and passive deformations of miniature soft robotic tools assist in detecting complex occlusions in lumens.

## INTRODUCTION

Minimally invasive endovascular operations through microcatheters have offered new intervention opportunities to treat various diseases in vessels, including the ones accompanied by the internal morphological changes of vascular lumens, such as stenosis (*1*, *2*), chronic total occlusion (CTO) (*3*, *4*), aneurysm (*5*, *6*), and dissection (*7*, *8*). A proper understanding of the details of these changes is beneficial for the precise localization, accurate diagnosis, and further proper treatments of these diseases on time. However, most state-of-the-art imaging techniques based on medical imaging and microcatheters, including contrast agent–based imaging and intravascular imaging techniques, have difficulties in detecting the lesion’s fine features (*9*–*13*) due to the limited distribution of blood flow and accessibility in complex vessels by existing tools (please refer to table S1 for their detailed comparison). These limitations could be caused by several factors. First, given the complex flow distributions around the internal morphological changes, it is challenging to diffuse contrast agents evenly and continuously along the lumen both axially and radially to visualize all the detailed endovascular features, such as the entry point of occlusions (*14*, *15*) and the shape of atherosclerosis (*16*, *17*). As a result, some contrast agent–dependent imaging techniques, i.e., x-ray, computed tomography, ultrasonography, and magnetic resonance imaging, struggle to accurately depict the details of the internal morphology (*16*, *18*). Second, some intravascular imaging, e.g., intravascular ultrasound and optical coherence tomography, which are usually combined with relatively stiff cables and components, usually sense the features along the cross-section plane perpendicular to the lumen axis (*3*, *19*–*21*), which make it challenging to visualize the details ahead when advancing tools (*22*–*24*) and imposes the risk of hitting blood clots and entering subintimal space of vessels during advancement in the vasculature with notable tortuosity and narrowing (*25*–*28*). Although some advanced imaging methods with high resolution, e.g., photoacoustic imaging, have been used for preclinical and clinical diagnosis in recent years, they have limited penetration depth up to around 4 cm in vivo (*29*, *30*), e.g., they have difficulty in detecting deeply located vasculatures, internal organs, and diseased tissues (*31*, *32*).

As typical examples, limited by the diffusion of the contrast agent and the access of intravascular imaging devices, it is clinically challenging to detect, i.e., visualize and cross, the detailed morphology of the narrow vessels, which are due to diseases, such as stenosis (narrowing of blood vessels by fatty deposits) (*1*, *33*) and CTO (the vessel’s complete or nearly complete occlusion by the heavy atherosclerotic plaques within the artery) (*34*, *35*). Consequently, there are clinical difficulties in making an accurate diagnosis and developing a timely treatment plan, e.g., the grade of partial occlusions and the following size selection of the stenting/ballooning are hard to properly decide (*28*, *36*) and the microchannels (MCs) inside severe occlusions are nontrivial to cross (*14*, *15*). Those delayed treatments may lead to further serious complications, such as heart attack, stroke, and even death (*37*–*39*).

Over the past few years, the advancements in microfabrication and soft robotics fields have led to the development of miniature soft robotic devices with remarkable locomotion capabilities and exceptional adaptability to complex and constrained environments (*40*–*45*). Merging these technologies has opened up exciting possibilities for the advancement of medical devices (*13*, *46*–*59*), holding notable potential to improve the diagnosis and treatment of various diseases. Building upon the benefits of soft robot designs and microfabrication techniques, we propose a miniature soft tool with an active deformation segment (ADS) and a fluid drag-driven segment (FDS) that can properly visualize and cross the occluded lumens with complex internal structures. We investigate the tool’s performance in various vascular occlusions, which can occur in various body parts (for details on the physiological features of the possible occlusions, please refer to note S1), and we use the coronary artery as a reference for simulants. First, using the soft-bodied deformation and interactions, the ADS could detect the structural details of stenosis-like partial occlusions, which were hard to be visualized by the dissolved dye in the flow, in real-time with an average visualization error down to around 0.06 mm. Second, by leveraging the fluid-structure interactions with the pulsatile fluid flow, the FDS could automatically and selectively detect the entry point and CTO-like severe occlusions with complicated MCs whose diameters are down to 0.2 mm and curvature radius of the route as small as 0.33 mm. Last, we validated the tool’s functionality, i.e., visualizing and crossing the complicated internal structures of tubular structures, in both biologically relevant phantoms and ex vivo organs. The working regions of this flow-driven tool could be expanded to distal vascular networks, potentially allowing for applications in the vasculature that are difficult to reach with existing medical tools and offering a new solution in dealing with multiple types of vascular occlusions.

## RESULTS

### Design of the magnetically assisted milli-tool

**Fig. 1.**
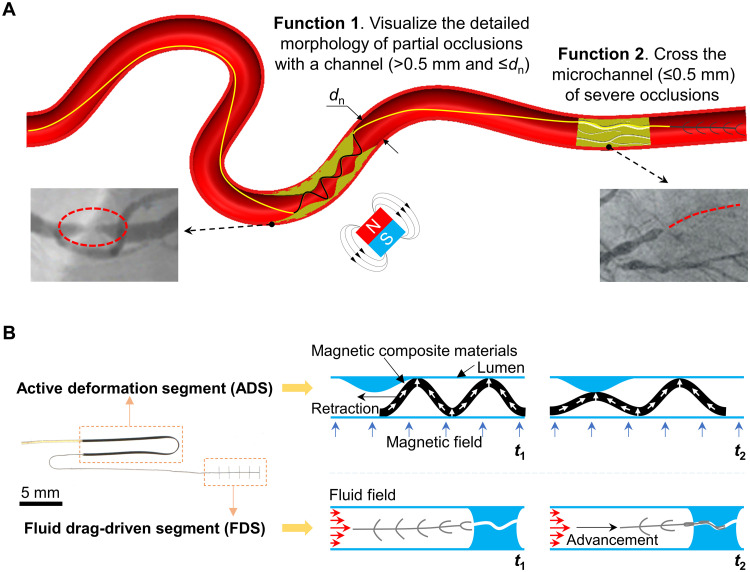
Design of the magnetically assisted milli-tool for occlusion detection. (**A**) Conceptual schematics of the two functions of the proposed milli-tool. The first function is to visualize the morphology of partial occlusions during retraction. The second function is to detect the entry point and cross the severe occlusion with complex internal structures (minimum diameter *d*_c_min_ down to 0.2 mm). (**B**) Details of the tool design. The tool has two functional segments and can be connected to various devices or robots. ADS with a programmed magnetization profile is used for the morphological visualization of partial occlusions. The FDS is an array of beam-like structures which could leverage the fluidic drag to detect the entry point and cross the severe occlusion. Medical images were reproduced from (*36*) and (*60*) under the CC BY license.

While stenosis usually represents an abnormal narrowing of blood vessels (*36*), CTO is a severe closure of the vessel lumen for at least 3 months with only micrometer-sized, random-distributed MCs (*60*), whose minimum diameter *d*_c_min_ is not larger than 0.5 mm and cross-section area *A*_mc_ is not larger than 2.4 mm^2^ (Fig. 1A). Accordingly, we define the stenosis-like occlusion as partial occlusion, containing a channel where *d*_c_min_ > 0.5 mm and ≤ *d*_n_ (the diameter of the uniform-sized lumen). In contrast, we define CTO-like occlusion as severe occlusion, containing one or several MCs, where *d*_c_min_ ≤ 0.5 mm. For the details, please refer to the physiological features of the possible occlusions in note S1.

To deal with these occlusions of different levels, we designed two major segments on the principal tool, a thin and long magnetic composite segment ADS, whose length *l*_ADS_ is 20 mm, width *w*_ADS_ is 0.28 mm, and thickness *t*_ADS_ is 0.05 mm, and a purely passive segment FDS, whose length *l*_FDS_ is 5 mm, width *w*_FDS_ is 0.15 mm, and thickness *t*_FDS_ is 0.04 mm. There is an array of soft beams with length *l*_b_ = 0.6 mm, width *w*_b_ = 0.15 mm, and thickness *t*_b_ = 0.02 mm designed on the FDS (Fig. 1B and fig. S1) (the nomenclature of the variables could be seen in table S2). The Young’s modulus of the ADS and FDS is around 3.92 ± 0.30 MPa and 0.50 ± 0.08 MPa, respectively, potentially enabling much safer interactions in vasculature compared with the medical devices, e.g., catheters and guidewires, made by various materials with modulus in the range of 0.1 to 300 GPa (*61*).

The ADS was composed of the poly(dimethylsiloxane) (PDMS) elastomer (Sylgard 184, Dow Inc.) loaded with neodymium-iron-boron particles (NdFeB, 5 μm, Magquench GmbH) and fabricated by laser cutting. The magnetization profile of ADS was preprogrammed with a vibrating-sample magnetometer (VSM; EZ7, Microsense), providing a uniform magnetic field of 1.8 T. The ADS could deform into a sinusoidal shape under the external actuation magnetic field when positioned in a uniform-sized lumen. Given internal occlusions, the soft ADS could adapt to surrounding complex environments and deform into various shapes. Thus, continuous monitoring of the shape changes of ADS while retracting it could provide detailed morphological information of the partial occlusions.

The FDS was fabricated by a soft polymer (Dragon Skin 30, Smooth-On Inc.) using optical lithography and molding techniques. The beams on it were bent by the fluidic drag from the incoming flow, and the complete tool was carried toward the area with the highest flow velocity. Notably, the well-designed soft beams with increased projection areas rather than a single wire could effectively leverage the fluidic drag for advancement, detecting the entry point of severe occlusions and even crossing them automatically via the inherent compliance of the soft body. For the details of the fabrication of these two segments, please refer to the “Fabrication of the tool” section in Materials and Methods.

### Morphological visualization of partial occlusions by the ADS

**Fig. 2.**
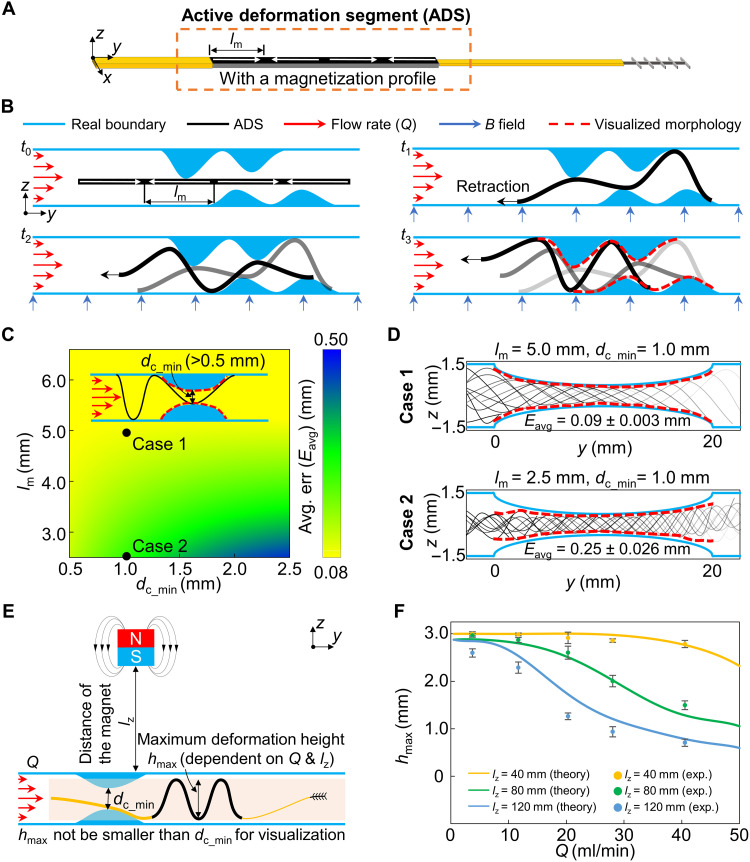
Morphology visualization of the partial occlusions using the proposed tool. (**A**) Design of the ADS with the programmed magnetization profile. (**B**) Morphological visualization principle of partial occlusions. (**C**) Influence of the magnetization segment length *l*_m_ and minimum diameter of the channel inside occlusions *d*_c_min_ on visualization error *E*_avg_ in phantoms A to E. *E*_avg_ was the average of absolute deviations of the visualized morphology (red dotted lines) from the real boundaries. *E*_avg_ increased as *l*_m_ decreased and *d*_c_min_ increased. (**D**) Two typical cases of morphology visualization. The black lines represent ADS at different positions during retraction. The pulsatile flow rate *Q* into the inlet was set to around 10.0 ml/min. (**E**) Investigation on the influence of *Q* and the distance between the magnet and the lumen *l*_z_ on the maximum deformation height *h*_max_ of the ADS. To successfully visualize occlusions in a lumen, *h*_max_ should not be smaller than *d*_c_min_. (**F**) Experimental and theoretical results of the investigation in phantom F. Error bars represent the standard deviation (SD) (*N* = 5). The retraction speed *v*_r_ of the tool was 1.0 mm/s in (C) and (F), and *l*_z_ was 50 mm in (C).

The tool visualized the morphology of stenosis-like partial occlusions by the outermost points of deformed ADS during retraction (Fig. 2, A and B). When a magnet providing the magnetic field approached the ADS, the ADS could deform and adapt to the surrounding occlusions; hence, we could obtain the positions of points on luminal borders by extracting the locations of the outmost points of the deformed ADS. Then, during the retraction, the deformation of the ADS was continuously changed to adapt to the surrounding boundary, i.e., contact points between the ADS and the real boundary were continuously changing. Therefore, the morphology of the occlusion could be visualized by combining the outermost points of the deformed ADS at different time steps. When the tool was retracted in a lumen with the pulsatile fluidic flow, the visualization results could be influenced by the following factors: (i) the morphology of the occlusion, which was quantified by the minimum diameter of the channel inside the occlusion $d_{c_{\min}}$ and the size of the internal plaques or thrombi *R* (subsequently termed as plaques), (ii) the length of the magnetization segment *l*_m_, (iii) the flow rate *Q*, (iv) the distance between the magnet and the lumen *l*_z_, (v) the retraction speed *v*_r_, and (vi) the viscosity of the flow η (the nomenclature of the variables could be seen in table S2).

We first investigated the effects of *l*_m_ and *d*_c_min_ on the accuracy of visualization. We fabricated various prototypes with different *l*_m_ ranging from 2.5 to 10.0 mm, which were connected to the medical tubings [inner diameter (ID) = 0.7112 mm, polyurethane medical tubing, Nordson Medical]. In all prototypes, the length and thickness of the connection segments C1 (*l*_C1_, *t*_C1_) and C2 (*l*_C2_, *t*_C2_) were the same as the ADS and the width of C1 and C2 were *w*_C1_ = 0.28 mm and *w*_C2_ = 0.10 mm, respectively (fig. S1). The investigations were conducted in three-dimensional (3D) printed phantoms A to E (material: Clear V4, printer: Form 3B, Formlabs Inc.) with partially occluded lumens; the data were collected using a commercial camera (Blackfly S USB3, Teledyne FLIR LLC) and the compatible software (SpinView 2.4.0). The fabrication details of phantoms can be seen in table S3 and in the “Preparation of simulants” section in Materials and Methods. In phantoms A to E, the diameter of the uniform-sized lumen *d*_n_ was 3.0 mm, and the length of occluded areas *l*_o_ was 20 mm. The experimental setup can be seen in the “Experimental test platforms” section in Materials and Methods and fig. S2. On the basis of the physiologically relevant flow rate in vascular stenosis (please refer to note S1), we set the flow rate *Q* of pulsatile blood analog [η = 4.4 centipoise (cP)] pumped into all phantoms to be around 10.0 ml/min with 80 beats per minute in the experiments. Besides, the retraction speed *v*_r_ of the tool was set to 1.0 mm/s, and the distance between the cubic actuation magnet (50 mm, N45, IMPLOTEX GmbH) and the lumen *l*_z_ was set to 50 mm. To quantify the accuracy of visualization using our approach, we defined a metric, the visualization error *E*_avg_. To compute it, we first visualized the body profiles of the ADS every 0.5 mm after it started to be retracted to enter the partial occlusion (fig. S3A). Second, we sampled the intersection points between these collective body profiles and the sampling lines. These lines were perpendicular to the lumen central axis and set every 0.5 mm from the start to the end of the occlusion (fig. S3B). Third, we connected all outermost intersection points on the upper and lower sides via straight lines, respectively (fig. S3C). Last, we computed the absolute deviation of the above two lines from the real boundaries on both sides at each sampling line and averaged all the deviations to acquire the final *E*_avg_.

Experimental results showed that *E*_avg_ increased as *l*_m_ decreased and *d*_c_min_ increased (Fig. 2C). For example, for the tool with *l*_m_ = 5.0 mm and occlusion with *d*_c_min_ = 1.0 mm, the visualization results by our approach matched the real luminal boundary well, and *E*_avg_ was only 0.09 ± 0.003 mm. In contrast, when *l*_m_ was 2.5 mm, visualization of the same occlusion with *d*_c_min_ = 1.0 mm mismatched the real luminal boundary, and the mismatch occurred mainly in the regions without occlusions. Notably, *E*_avg_ increased to 0.25 ± 0.026 mm (Fig. 2D and fig. S4). Besides, *E*_avg_ increased with the increase of *d*_c_min_ mainly occurring when *l*_m_ was smaller than 5 mm since the deformed ADS with short *l*_m_ was difficult to contact the boundary (fig. S5), while, for the tools with *l*_m_ more than 5.0 mm, *E*_avg_ remains almost constant for occlusions with different *d*_c_min_. It was worth mentioning that, for the latter case mentioned above, more contact points between the deformed ADS and the luminal boundary could be visualized for each time step, which might be useful for the operator to retrieve more information (fig. S5). Thus, we set *l*_m_ = 5.0 mm for further investigations.

Occluded vessels are often accompanied by complex internal structures, e.g., diffuse stenosis, which is characterized by multiple or especially long, continuous plaques within the vessels (please refer to note S1). On the basis of the above fundamental investigations, we further investigated the performance of the tool in two scenarios: cases with uniformly distributed smooth plaque simulants in occlusions (*l*_o_ = 20 mm) (phantoms A and T to U) (fig. S6) and extreme cases with a triangular-shaped plaque simulant in occlusions (*l*_o_ = 5.0 mm) (phantoms V to X) (fig. S7). In all investigations, *d*_n_ = 3.0 mm, *d*_c_min_ = 0.5 mm, and *Q* = 10.0 ml/min. In both scenarios, the results indicated that *E*_avg_ increased with the decreases of the size of plaques *R*. It should be mentioned that, although the detailed morphologies were unavailable when *R* < 2.5 mm, the *d*_c_min_ of the occlusion could still be detected.

Clinically, the flow rate *Q* of the blood in vessels correlates to the stenosis grade, e.g., *d*_c_min_ of the occlusion, and the minimum allowed distance between the magnet and the lumen *l*_z_min_ varies for different organs and tissues given the physical constraints (please refer to note S1). To ensure the effectiveness of the tool, we theoretically analyzed the effects of the distance between the magnet and the lumen *l*_z_ on the magnetic force *F*_m_ and magnetic torque *T*_m_ inducing the deformation of the ADS (fig. S8) and experimentally investigated the influence of *Q* and distance *l*_z_ on *E*_avg_ in the occluded lumen with uniform plaques (phantom T, *d*_n_ = 3.0 mm, *d*_c_min_ = 0.5 mm, *l*_o_ = 20.0 mm, *R* = 2.5 mm) (fig. S9). Investigation results showed that, in the lumen with the higher *Q*, *E*_avg_ also was higher because the drag force reduced the deformation of the ADS. On the other hand, the smaller *l*_z_ favored decreasing *E*_avg_ of the tool because of the larger magnetic torque. As the morphology visualization of the occlusion was realized by the tool’s retraction, we also investigated the influence of retraction speed *v*_r_ from 0.5 to 3.0 mm/s on *E*_avg_ in phantom T. The results showed that, in the range that we investigated, the differences among *E*_avg_ at various *v*_r_ were not significant [*P* = 0.998, one-way analysis of variance (ANOVA) test] (fig. S10). Considering the healthy state of the human body, η could be changed in the range of 4 to 45 cP (please refer to note S1). According to our investigation, varying η in the range of 0.9 to 50.0 cP did not lead to significant differences in visualizing the partial occlusions by ADS (*P* = 0.566, one-way ANOVA test) (fig. S11A).

As a summary of the previous investigations, the morphology visualization of occlusions relied on the contact of deformed ADS and the luminal boundary. Thus, to successfully detect the occlusion areas/points, the maximum deformation height *h*_max_ of the ADS should not be smaller than *d*_c_min_ as shown in Fig. 2E. Here, we developed a theoretical model to predict *h*_max_ of ADS (see the “Modeling of the deformation of the ADS” section in Materials and Methods and note S2). The model matched well with the experimental results of visualization in the phantom F (without occlusions and *d*_n_ = 3.0 mm) (Fig. 2F), which provided guidance for designing a proper tool in various scenarios. For example, in terms of 75% stenosis in arteries with *d*_n_ = 3.0 mm, *Q* is around 50.0 ml/min, and *d*_c_min_ is 1.5 mm (the cross section of the channel is assumed to be circular-shaped) (*62*), *h*_max_ is estimated to be 2.3 mm when *l*_z_ is 40 mm and 1.0 mm when *l*_z_ is 80 mm. The tool should then be applicable in the scenarios of peripheral, carotid, and coronary arteries, as the *l*_z_ for those arteries is in the range of 20 to 40 mm (*63*–*65*). In contrast, the tool might be challenging to be used for detecting the above stenosis in the renal artery as the *l*_z_min_ could be larger than 80 mm (*64*).

### Benefits for visualizing complex partial occlusions

**Fig. 3.**
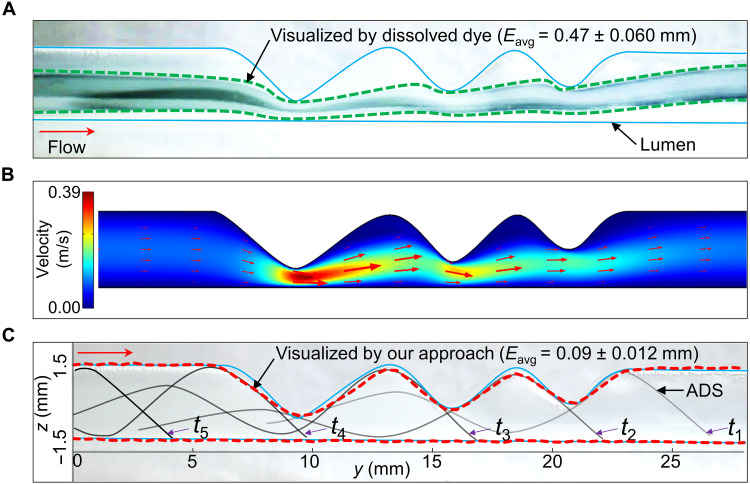
Benefits of visualizing the complex partial occlusions. (**A**) Morphology of phantom G visualized by the dissolved dye in the flow. (**B**) Computational fluidic dynamics investigation of the flow distribution in the lumen. (**C**) Morphology visualized by our proposed approach. The method relying on the dissolved dye in the flow could be highly affected by the flow distribution across the lumen, i.e., highest in the center, lowest close to the boundary. In contrast, our tool could actively deform and adapt to the real boundary to visualize the detailed morphologies. The flow rate *Q* was around 20.0 ml/min, the distance between the magnet and the lumen *l*_z_ was 50 mm, and the retraction speed *v*_r_ was 1.0 mm/s in (C).

Partial occlusions, such as stenosis, are usually detected by contrast agents clinically. However, given the pure dependence on flow distributions and usually insufficient uniform axial and radial diffusions, detailed morphological information of these occlusions is challenging to retrieve (*66*, *67*). Consequently, the proper sizing and deployment of the following medical devices are affected negatively. As a potential solution, we demonstrated that our tool could assist in depicting the local details of these partial occlusions. The benefits of using our approach were demonstrated in phantom G (table S3), which had complicated occlusions containing different plaques-like structures along the axial direction (Fig. 3 and movie S1). The pulsatile blood analog pumped into this phantom was set to around 20.0 ml/min. We first injected some dissolved food dye (volume ratio of 50 to 1 in deionized water, Airbrush Farben, Bakeryteam) as a basic simulant of the contrast agent, using a syringe (Injekt-F Solo, B. Braun Company) at the speed of around 1.0 ml/s, which is comparable to the injection speed (0.5 to 5.0 ml/s) of medical contrast agent (please refer to note S1). As seen in Fig. 3A, the dye mostly diffused in the axial direction along with the flow, and its diffusion to the radial direction of the lumen was not complete, especially in the occlusion area, making it troublesome to visualize the local details of boundaries. This phenomenon could be explained by the flow distributions as quantified by the computational fluidic dynamics simulations in COMSOL Multiphysics (COMSOL 5.6, COMSOL Inc.), where the flow with the highest speed was centralized in the middle of the lumen, centralizing the dye, especially for the occluded regions (Fig. 3B). As a result, the visualized morphology showed poor details, and the visualization error *E*_avg_ reached 0.47 ± 0.06 mm. In contrast, the ADS could actively deform and adapt well to complex boundaries, and the morphology visualized by our approach matched the real lumen well with a smaller *E*_avg_ = 0.09 ± 0.012 mm (Fig. 3C). Furthermore, the ADS could be used for local drug delivery applications. As a proof of concept, using the simulant (fluorescein sodium, Fisher Scientific UK Limited) enveloped in a customized carrier fabricated by Dragon Skin 30, we demonstrated that the deformed ADS could release the cargo to the vicinity of the occluded luminal walls (*t*_3_ – *t*_6_ in fig. S12). This contrasts the passive drug release diffusing to the lumen’s center given the flow distribution (*t*_2_ in fig. S12). Such a local drug application exactly on the lesions could be beneficial for the clinical treatments of occlusions (*68*, *69*).

### Visualization of 3D complex partial occlusions

**Fig. 4.**
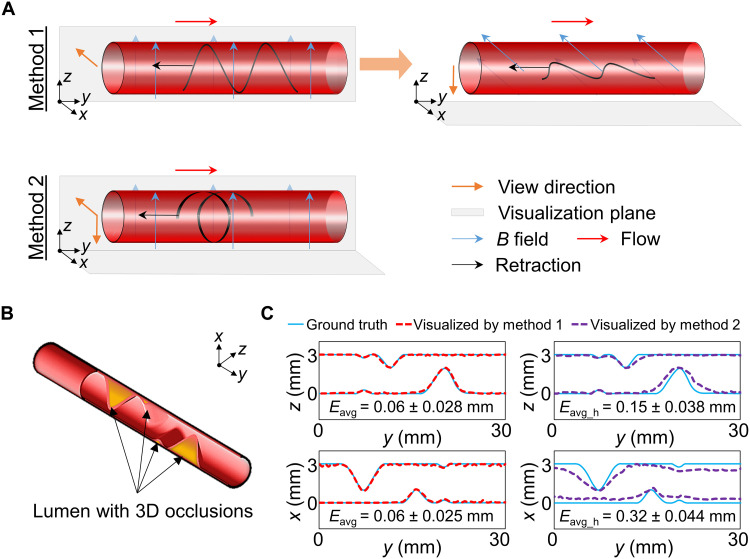
Visualization of the 3D complex partial occlusions. (**A**) Principle of two 3D morphology visualization methods. Method 1 deploys and visualizes the tool along two perpendicular planes independently and sequentially in two retractions. Method 2 deploys and deforms the helix-shaped tool once, then visualize it along two perpendicular planes simultaneously in one retraction. (**B**) Phantom H with 3D occlusions. (**C**) Results of method 1 and method 2. The visualization errors *E*_avg_h_ of method 2 were relatively higher, while method 1 might be inefficient given two sequential steps. The ADS for 3D visualization was actuated by a uniform magnetic field (around 50 mT, parallel with the *yz* plane in method 2). The flow rate *Q* and retraction speed *v*_r_ in (C) were around 12.0 ml/min and 1.0 mm/s, respectively.

In realistic scenarios, the internal occlusions can be 3D structures along the circumference of the lumen. We proposed two methods to deal with it (Fig. 4A). The first one is to use the existing design with 2D deformation, where we could deploy and visualize the tool along two perpendicular planes independently and sequentially in two retractions. The second one is to revise the design to achieve 3D deformation, e.g., a helix shape. We could deploy and deform it once, then visualize it along two perpendicular planes simultaneously in one retraction. For such an ADS design, the thickness *t*_ADS_ was increased to 0.25 mm to avoid the local twisting under the magnetic field (fig. S13). Besides, the length *l*_ADS_ was changed to 31.4 mm, which allowed the ADS to be wrapped twice around a nonmagnetic rod with a diameter of 5 mm for magnetization. We evaluated the visualization by two methods in phantom H (table S3), which is with four plaques distributed at different axial and circumferential locations (Fig. 4B). The pulsatile flow rate *Q* and the retraction speed *v*_r_ was set to around 12.0 ml/min and 1.0 mm/s, respectively. During evaluations, two ADSs were both actuated by a uniform magnetic field with a magnitude of around 50 mT provided by a Halbach array.

In method 1, we first viewed the frontal morphology of the occlusion in the *yz* plane, and then, by reorienting the magnetic field, we viewed the morphology of the occlusion in the *xy* plane from the top by retraction again. The errors *E*_avg_ in two visualizations were 0.06 ± 0.028 mm and 0.06 ± 0.025 mm, respectively (Fig. 4C). In method 2, we set the orientation of the magnetic field parallel with the *yz* plane and simultaneously visualized the morphology of the occlusion in two planes. During the retraction, the helix shape of the ADS might be hard to maintain, given the friction in the axial direction. Therefore, for each retraction of 2.5 mm, we rotated the magnetic field along the *y* axis to recover a helix shape and then sampled the deformation from two planes, as suggested by Ren *et al*. (*59*). Visualization errors *E*_avg_h_ for this method were 0.15 ± 0.038 mm and 0.32 ± 0.044 mm, respectively (Fig. 4C). While method 1 could have a relatively high accuracy of visualization from two views, it might be inefficient given two sequential steps. In contrast, although the errors for method 2 were more notable, it could provide a quick insight into the geometry of the occluded areas from simultaneous views. Thus, these two methods can be selected according to particular needs.

### Cross the severe occlusions by FDS

**Fig. 5.**
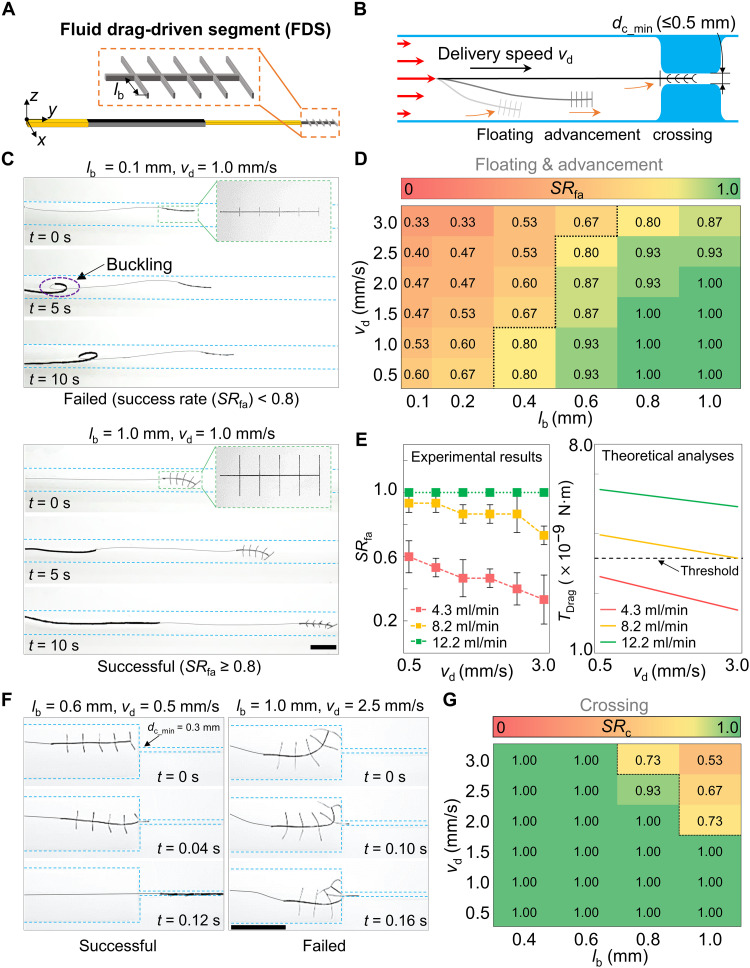
FDS of the proposed tool for crossing the severe occlusions. (**A**) Design of FDS. The length of the beams in the *y* and *z* directions was 0.02 mm and 0.15 mm, respectively. (**B**) Principle of tool floating, advancing in the normal lumen, and crossing the severely occluded lumen. (**C**) Two typical cases of FDS advancing in the lumen. When *l*_b_ = 0.1 mm, the tool failed to advance due to buckling. (**D**) Effects of delivery speed *v*_d_** and length of beams *l*_b_ on the success rate of floating and advancement (*SR*_fa_) of the tool in the normal region of phantom I. We considered the design with *SR*_fa_ ≥ 0.8 to be optimal. (**E**) Effects of flow rate *Q* and *v*_d_** on the *SR*_fa_ (*l*_b_ = 0.1 mm). Experimental results and theoretical analyses are on the left and right sides, respectively. The threshold *T*_t_ = 4.18 × 10^−9^ N·m is the torque produced by the gravity and buoyancy of the tool. *T*_Drag_ is the torque produced by the drag on the tool. Increasing the *T*_Drag_ by changing the flow was favorable to improving *SR*_fa_. (**F**) Two typical cases of FDS crossing the MC (minimum diameter *d*_c_min_ = 0.3 mm) in a severely occluded region of phantom I. (**G**) Effects of *l*_b_ and *v*_d_ on *SR*_c_. Larger *l*_b_ and higher *v*_d_** have a negative influence on *SR*_c_. The pulsatile flow rate *Q* was 3.6 to 5.0 ml/min in (D) and (G). Error bars represent SD (*N* = 9). All scale bars, 3 mm.

The second segment of the tool with an array of beam-like structures, FDS (Fig. 5A), could leverage the fluidic drag to float, advance, locate, and cross the severe occlusions with narrow MCs, where the minimum diameter *d*_c_min_ ≤ 0.5 mm and the low flow rate *Q* < 10.0 ml/min (for details on the physiological features of the possible occlusions, please refer to note S1) (Fig. 5B). Its performance was affected by several key design and environmental parameters, including (i) lengths of beam *l*_b_, (ii) delivery speed *v*_d_, (iii) flow rate *Q*, and (iv) viscosity of flow η (the nomenclature of the variables could be seen in table S2).

We started by investigating a class of prototypes with various *l*_b_ ranging from 0.1 to 1.0 mm and *v*_d_ ranging from 0.5 to 3.0 mm/s. The prototypes were tested in phantom I (table S3), which has a severely occluded lumen with an MC located in the occluded region. The MC has a circular cross section with *d*_c_min_ = 0.3 mm and length *l*_mc_ = 15.0 mm (see the “Preparation of simulants” section in Materials and Methods). To quantify the performance, we defined a metric, success rate of floating and advancement *SR*_fa_, which is the ratio between the times the prototype successfully advanced 60 mm in the normal region of the lumen and the times of tests. We regarded that the advancement succeeds when *SR*_fa_ ≥ 0.8. The pulsatile blood analog pumped into this phantom was set to around 4.0 ml/min, which is in the same order of magnitude as the blood flow rate seen in CTOs. Experiments have shown that the tool with shorter *l*_b_ and higher *v*_d_ was prone to buckle during advancement, leading to the failure of the advancement and the decrease of *SR*_fa_ (Fig. 5, C and D).

To further understand the impact of flow rates, using the tool with *l*_b_ = 0.1 mm, we investigated the effects of *Q* on *SR*_fa_ when *v*_d_ was in the range of 0.5 to 3.0 mm/s. Experimental results showed that a larger *Q* was favorable to improving the *SR*_fa_ (Fig. 5E). To understand the underlying physics, we theoretically analyzed the influence of *Q* and *v*_d_ on the floating and advancement of the tool (note S3). To achieve successful floating and advancement, the applied torques from fluidic drag *T*_Drag_ and buoyancy *T*_b_ should be larger than the torques from gravity *T*_g_

$$T_{\mathrm{Drag}}+T_{b}>T_{g}$$ (1)

Since *T*_b_ and *T*_g_ are fully decided by the tool’s design and the properties of the blood analog, the feasible floating and advancement should require the *T*_Drag_, which is determined by *Q* and *v*_d_, to satisfy the following relations

$$T_{\mathrm{Drag}}(Q,v_{d})>T_{g}-T_{b}$$ (2)

For the convenience of illustration, we defined the threshold as *T*_t_ = *T*_g_ − *T*_b_ Given the chosen designs and the settled experimental setup, *T*_t_ was calculated to be 4.18 × 10^−9^ N·m. For various *Q* and *v*_d_**, *T*_Drag_ was computed and shown in Fig. 5E. These quantified results matched well with the experimental phenomenon. When *Q* was 4.3 ml/min, *T*_Drag_ was smaller than *T*_t_ for all *v*_d_**, indicating an impractical floating and advancement of the tool, which was in line with the experimental results that *SR*_fa_ was very low. In contrast, when *Q* was 8.2 and 12.2 ml/min, *T*_Drag_ was larger than *T*_t_, indicating a feasible tool delivery that can be seen in the experimental results where *SR*_fa_ was high.

Using prototypes with relatively high *SR*_fa_, i.e., *l*_b_ = 0.4 to 1.0 mm, we further investigated the success rate of crossing the MC, *SR*_c_, in the same phantom. The tool’s delivery started at 20 mm in front of the occlusion, and the delivery success rate *SR*_c_ was the ratio of the times that the tool passed the MC over the times that it was successfully delivered to the proximal end of the occlusion. Experiments indicated that the tool with longer *l*_b_ and higher *v*_d_ tended to be stuck at the entrance of the MC, as seen in Fig. 5F. The slower *v*_d_ and shorter *l*_b_ were favorable to improving the *SR*_c_ of the tool’s crossing (Fig. 5G). A higher *Q* was also favorable to improving the *SR*_c_ of the tool’s crossing (fig. S14). Furthermore, our investigation showed that there was no significant difference in *SR*_fa_ (*P* = 0.415, one-way ANOVA test) and *SR*_c_ (*P* = 0.452, one-way ANOVA test) when the tool crossed severe occlusions for η = 0.9 to 50.0 cP (fig. S11B).

In summary, lower *v*_d_ = 0.5 to 1.0 mm/s and medium *l*_b_ = 0.6 to 0.8 mm were favorable for the tool’s floating, advancement in the lumen, and crossing the severe occlusions. Thus, we selected *v*_d_ = 0.5 mm/s and the tool with *l*_b_ = 0.6 mm for further investigations.

### Demonstrations of locating the entry point and crossing severe occlusions

In addition to a very low flow rate, severe occlusions like CTO contain complicated internal structures. For example, the MCs inside could be tortuous and have multiple points extending to various locations, e.g., vessel walls, the distal end of the vessel, and even stopping in the middle (false MC) (please refer to note S1). This complexity has notably raised the clinical challenges in successfully identifying the suitable entry point of CTOs and crossing them for the following diagnosis and treatments. Leveraging the tool’s unique advantages in automatically crossing these severe occlusions, we further validated its effectiveness in the phantoms with physiologically relevant features (see the “Preparation of simulants” section in Materials and Methods).

**Fig. 6.**
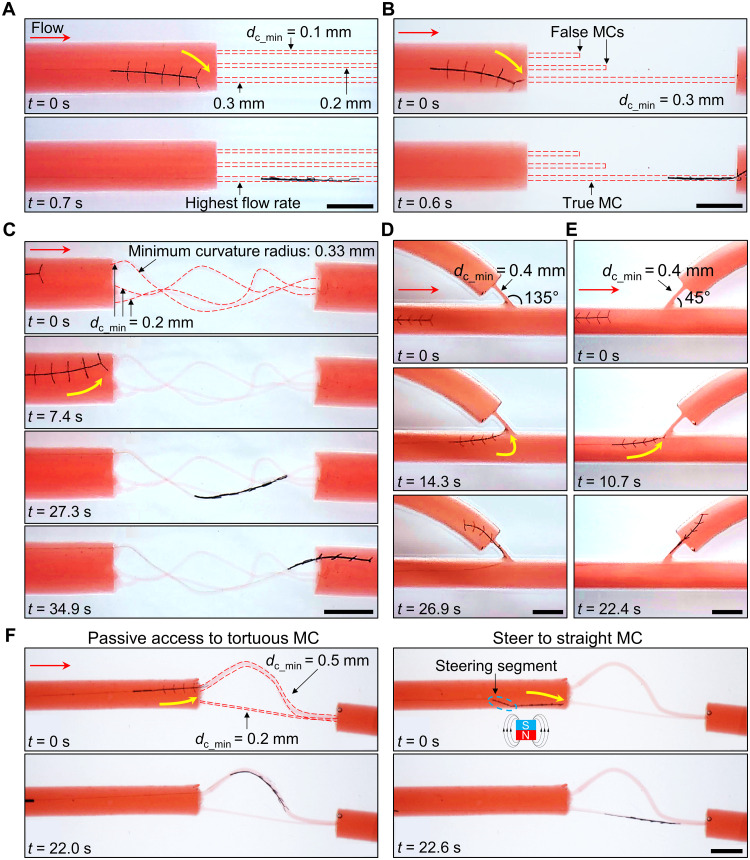
Demonstrations of the tool locating the entry point and crossing the severe occlusions. (**A**) Crossing of the MC with the highest flow rate (phantom J). About 82% of the flow rate at the inlet was distributed into the MC with the largest diameter *d*_c_min_. (**B**) Crossing of true MCs (phantom K). (**C**) Crossing of tortuous MCs with diameters down to 0.2 mm and the curvature radius of the route as small as 0.33 mm (phantom L). (**D** and **E**) Crossing of MC on side branches [phantom M in (D), N in (E)]. (**F**) Crossing of MC with the relatively straight route (phantom O). The tool with a magnetic steering segment was steered by a cubic permanent magnet (20 mm), and the distance between the magnet and the lumen was around 40 mm. The length, thickness, and width of the soft beams in all experiments here were 0.6, 0.02, and 0.15 mm, respectively. The delivery speed was set to 0.5 mm/s. The pulsatile flow rate into the inlet was set to 3.6 to 7.9 ml/min (comparable to the flow rate in vessels with chronic total occlusion) in (A) to (C) and (F), and 18.0 to 22.1 ml/min (comparable to the flow rate in occluded vessels) in (D) and (E). All scale bars, 3 mm.

First, we demonstrated that our proposed tool could locate the entry point and cross the MC with the highest flow rate in the severe occlusion with multiple MCs (phantom J) (see table S3) (Fig. 6A and movie S2). The phantom contains three different MCs with a circular-shaped cross section and *d*_c_min_ was 0.1, 0.2, and 0.3 mm, respectively. *Q* was set to 3.6 to 7.9 ml/min, and *v*_d_ was set to 0.5 mm/s. When the tool was delivered to about 1 mm ahead of the occlusion, it was bent downward by the concentrated flow, located the entry point of the MC with *d*_c_min_ = 0.3 mm, and then successfully crossed this MC. We quantified the flow field in the abovementioned experimental conditions, which showed that when *Q* was 4.0 ml/min, about 82% of the flow was distributed into the MC with the largest *d*_c_min_ = 0.3 mm, 17% was distributed into the MC with *d*_c_min_ = 0.2 mm and 1% was distributed into the MC with *d*_c_min_ = 0.1 mm (fig. S15). This phenomenon of crossing the MC with the largest *d*_c_min_ remained consistent when the sequence of three MCs was shuffled (phantoms Y and Z) (figs. S15 and S16). Second, using the same experimental conditions as above, we demonstrated that the tool could avoid falling into the false MC and cross the true MC with a through channel (phantom K) (Fig. 6B and movie S3). Third, we demonstrated that the same tool could cross the MC with tortuous routes where *d*_c_min_ was down to 0.2 mm (phantom L) and the curvature radius of the route as small as 0.33 mm (Fig. 6C and movie S4). Fourth, in addition to crossing the MCs in the main branches, we demonstrated that the tool could cross MCs on the side branches (phantoms M and N, cross-section area of the MC *A*_mc_: 0.4 × 2 mm^2^) (Fig. 6, D and E, and movies S5 and S6), even with obtuse bifurcation angles (please refer to note S1). Here, we further investigated the influence of *Q* from 6.0 to 30.5 ml/min on *SR*_c_ of crossing the MC on branches when *v*_d_ was swept from 0.5 to 3.0 mm/s (fig. S17). Last but not least, we demonstrated that the crossing of MCs was steerable using a magnetic segment between the FDS and C2 (fig. S18). As shown in Fig. 6F and movies S7, in the phantom O, the tool driven by the flow automatically located the entry point and crossed a tortuous MC with larger *d*_c_min_ = 0.5 mm. In addition, when an external magnet was introduced, the tool could be steered toward a relatively straight MC with *d*_c_min_ = 0.2 mm. This enhanced feature has great potential to assist in the selection of the most proper MC for following operations.

### Functional demonstrations in physiologically relevant phantoms under x-ray imaging

**Fig. 7.**
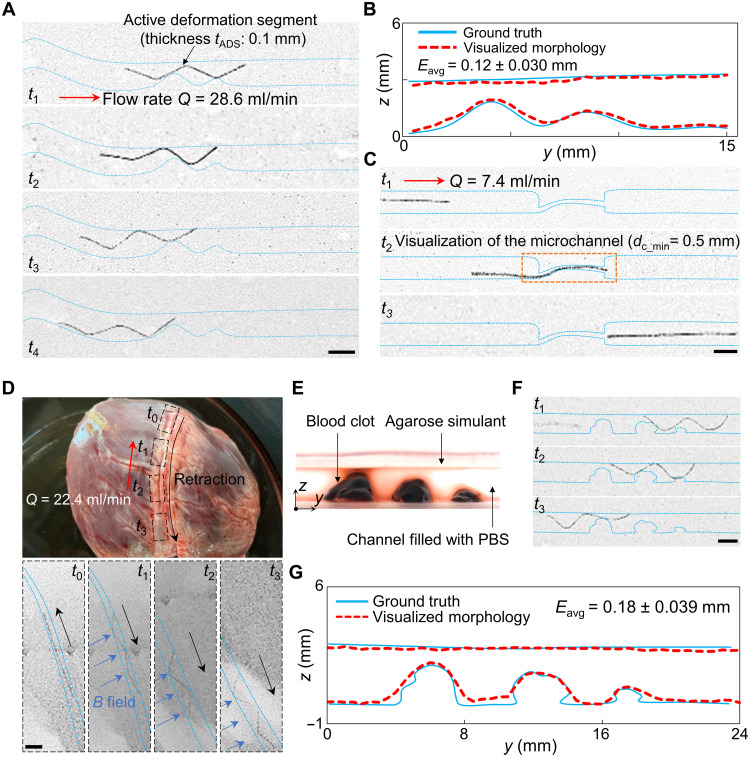
Demonstrations of the tool’s functions in physiologically relevant phantoms under x-ray imaging. (**A**) Morphology visualization of the partial occlusion in agarose-based phantom P. (**B**) Results of visualization. (**C**) MC crossing and visualization of severe occlusion in agarose-based phantom Q. The length and location of the MC (minimum diameter *d*_c_min_ = 0.5 mm) can be detected by the deformation of the tool. (**D**) Delivery and visualization of the tool in a coronary artery of the porcine heart. (**E**) Snapshots of blood clots immobilized in a PBS-filled channel (phantom R). (**F**) Morphology visualization process in phantom R. (**G**) Results of visualization. The distance between the actuation magnet (50 mm) and the lumen *l*_z_ was 50 mm in (A) and (D), and 100 mm in (F). The x-ray imaging parameters for all detections: 50 kV, 65 μA. All scale bars, 3 mm.

To evaluate the tool in more medically relevant settings, we demonstrated its functions in physiologically relevant phantoms under x-ray imaging using a cabinet x-ray system (XPERT 80, Cabinet X-ray System, KUBTEC Scientific) and the compatible operating software (KubtecNC 3.0.0.0). The imaging parameters were set to 50 kV and 65 μA, which are consistent with the range of parameters used for medical x-ray imaging (please refer to note S1). First of all, two major functions of the tool, i.e., morphology visualization of partial occlusions and crossing of the severe occlusions, were validated in phantoms fabricated by the agarose (A9539, Sigma-Aldrich), which is a tissue-mimicking material suitable for x-ray imaging (*70*, *71*). We fabricated two phantoms with a complicated partial occlusion (phantom P) and a severe occlusion containing an MC with *d*_c_min_ = 0.5 mm (phantom Q), respectively (see the “Preparation of simulants” section in Materials and Methods). The pulsatile flow rate *Q* pumped into phantom P was set to 28.6 ml/min (comparable to the flow rate in the condition with stenosis), and phantom Q was set to 7.4 ml/min (comparable to the flow rate in the condition with CTOs), respectively. The tool tested here was designed to be with *t*_ADS_ = 0.1 mm and *l*_C1_ = 20 cm to match the test environments. In phantom P, while the occlusion was unclearly shown using a contrast agent [Iomeron 400, Bracco UK Limited; mass ratio to phosphate-buffered saline (PBS): 1:1] with the injection speed of 0.4 to 1.2 ml/s (please refer to note S1) (fig. S19, A and B), it could be visualized by our approach with *E*_avg_ = 0.12 ± 0.030 mm when *l*_z_ was 50 mm (Fig. 7, A and B). In phantom Q, the location and length of the MC in severe occlusion could be detected via the deformation of the tool while crossing the MC (Fig. 7C), which could be challenging to be localized by the contrast agent (fig. S19, C and D).

To further validate the tool’s effectiveness, we confirmed that its interaction with soft tissue would not affect its function negatively. Here, we first delivered the tool in freshly cut porcine coronary arteries with varying diameters (see the “Preparation of porcine coronary arteries and blood clots for ex vivo tests” section in Materials and Methods), where *Q* was 22.4 ml/min and *l*_z_ was 50 mm. Under x-ray imaging, given the applied magnetic field, we could extract the internal borders of the lumen based on the deformation of the ADS at different times and rebuild the morphology of the lumen, which has been validated by medical ultrasound imaging (Vevo 3100, FUJIFILM Sonosite Inc.) using a linear array transducer (MX 550D, FUJIFILM Visualsonics Inc.) (Fig. 7D and fig. S20). Next, we demonstrated that depicting the morphological details of thrombi using our tool is feasible. We fixed some blood clots, prepared by fresh porcine blood, in a channel fabricated by the agarose and filled with PBS (phantom R) (Fig. 7E and fig. S21). Along with the retraction, our tool could detect the location and morphology of blood clots under x-ray imaging, with a relatively high accuracy of *E*_avg_ = 0.18 ± 0.039 mm when *l*_z_ was 100 mm (Fig. 7, F and G).

### Untethered mode of the tool integrated with a wireless stent-shaped millirobot

Delivering tethered medical devices to regions with highly tortuous vascular routes far from the incision could be challenging (*19*, *27*), which might lead to severe complications such as dissection, perforation, and vasospasm. Wireless medical millirobot prototypes have been developed to overcome the above challenges and access these hard-to-reach regions (*44*, *45*, *54*). With the well-controllable and adaptive segments, our tool could be integrated into wireless medical millirobots for visualizing and crossing occlusions. We demonstrated that the tool could work in the untethered mode by being connected to a wireless stent-shaped magnetic soft millirobot, Stentbot (*54*), whose length was 5.0 mm and outer diameter was 2.8 mm.

**Fig. 8.**
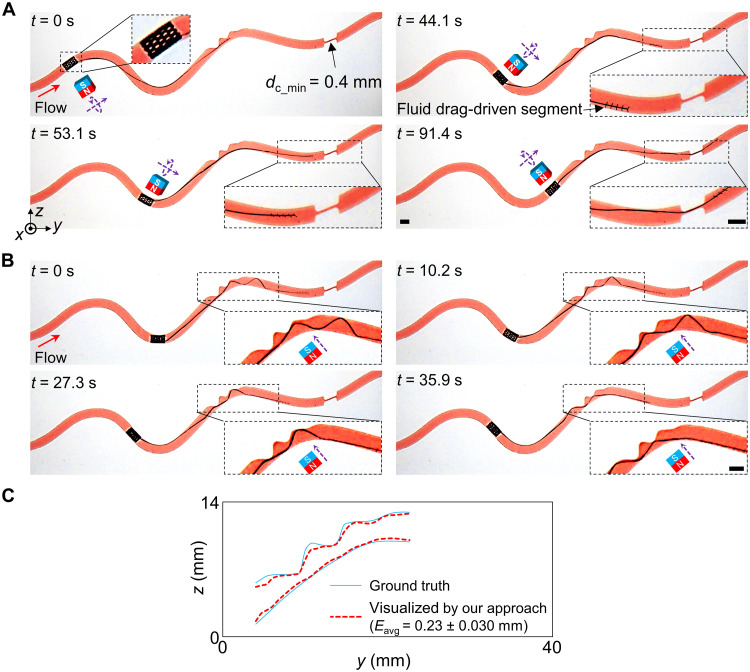
Untethered mode of the tool connected to a wireless stent-shaped soft millirobot. (**A**) Advancement and MC crossing in phantom S. Minimum diameter of the MC *d*_c_min_ = 0.4 mm. (**B**) Retraction and morphology visualization of the occluded lumen. The tool was attached to a stent robot steered by a translational and rotational permanent magnet. The magnet could also be used for morphology visualization. The morphology of the occlusion was visualized by the deformation of the ADS under the magnetic field when the stent robot dwelled at different positions. (**C**) Visualization results. The pulsatile flow rate *Q* into the inlet was set to around 14.0 ml/min. The distance from the cubic actuation magnet (20 mm) to the lumen was 40 mm when it was used for the steering of the stent-shaped millirobot and the deformation of the ADS. All scale bars, 3 mm.

The demonstration was performed in a 3D printed phantom (phantom S) (table S3) that contained various occlusions, i.e., a stenosis-like partial occlusion and a CTO-like severe occlusion with *d*_c_min_ = 0.4 mm and a cross-section area for the MC *A*_mc_ being set as 0.4 × 2 mm^2^. To reduce the negative effect of the deformed ADS on the tool’s navigation, we increased *l*_C1_ to 30 mm. Meanwhile, *l*_C2_ was changed to 10 mm to keep the overall length of the tool constant as before. Besides, the flow rate of the pulsatile blood analog pumped into this phantom was set to be around 14.0 ml/min. The Stentbot was steered by a translational and rotational permanent magnet (20 mm), which was placed 40 mm away from the phantom and manipulated by two servo motors and an X-Y stage (fig. S22). Assisted by the Stentbot, the FDS driven by the fluid drag could cross the CTO-like severe occlusion, i.e., locating the entry point and crossing the MC (Fig. 8A and movie S8).

Reversing the translation and rotation directions of the permanent magnet, the tool could be retracted by the Stentbot, which went against the incoming flow. During the retraction, the morphology of the lumen was visualized by the deformation of the ADS under the magnetic field when the Stentbot dwelled at different positions. For each 2.5 mm of retraction, the actuation magnet was moved to approach the ADS in the *yz* plane with the magnet placed 40 mm away and its moment’s orientation perpendicular to the lumen axis. After capturing the deformation of the ADS, the magnet was returned to the dwelling position again to steer the Stentbot to the next dwelling position, as shown in Fig. 8B and movie S9. After collecting all the outermost points of the deformed ADS at different positions, the morphology of the occlusion could be reconstructed using our approach. The morphology visualized by our approach matched the ground truth well with *E*_avg_ = 0.23 ± 0.030 mm (Fig. 8C).

## DISCUSSION

Given the challenge of insufficient detection, i.e., visualization and crossing, of the complicated occluded structures in lumens, we developed a millimeter-scale soft robotic tool with the synergy of two functional segments, i.e., ADS and FDS. Our experimental investigations with theoretical support have pointed out two key features of this device: ADS enables active soft-bodied deformation under external magnetic stimulus to visualize morphological details of the partial occlusions via interactions, which is hard to be precisely visualized by the dissolved matter in pulsatile flow; and FDS could leverage active steering and fluid-structure interaction to enable the selective crossing of the severely complicated occlusions. These functions have been evaluated in physiologically relevant phantoms and ex vivo organs.

The visualization of the tool under x-ray depends on various factors, such as the resolution of the device, the density of the metal particles, the size of the tool, and the presence of the calcified tissue in the occlusion. According to the needs of different applications, we could appropriately increase the size of the ADS and the ratio of its internal metallic components, i.e., NdFeB particles, to enhance the contrast of the tool.

In very low flow rates, the tool might be difficult to float and advance. Thus, polymers with low density can be adopted. On the other hand, with a very high flow rate, due to the relatively high drag force to extend the tool, ADS might be hard to deform fully, and the morphology visualization is discounted. Using the polymer with a lower Young’s modulus would favor the deformation of the ADS. Besides, increasing the ratio of magnetic particles in the composite can help maintain the performance as the higher magnetic torque deforming the ADS could be provided. The biocompatibility and hemocompatibility of these materials can be feasibly achieved (please refer to note S4) (*54*).

In medical applications, the sub-millimeter tool that we proposed can be combined with state-of-the-art devices, e.g., microcatheters and guidewires, to assist in improving the effectiveness of diagnosis and treatment of endovascular diseases accompanied by internal morphological changes, e.g., determining the location, grade, shape, and size of stenosis for sizing and placing the medical stents, locating the entry point and assessing the internal tortuosity of the MCs of CTOs for the following delivery of medical catheters. Given its soft design, one benefit of this tool is its safe interaction with the surrounding structure (please refer to note S5 and fig. S23) to minimize potential harm, such as dissection, perforation, and vasospasm, making it suitable for navigating in the tortuous, severely calcified, and narrowed lumen. Moreover, with the assistance of the fluidic drag, the operators could ease the difficulty of the crossing of CTO by purely relying on the insertion and torquing of the guidewires at the proximal end. Toward clinical applications, multiple measurements could help enhance accuracy. Accordingly, we have quantified and confirmed the mechanical durability of the tool after several rounds of delivery and retraction, as shown in fig. S24.

## MATERIALS AND METHODS

### Fabrication of the tool

The tool we proposed comprised two functional segments, i.e., ADS and FDS, and two connection segments (C1 and C2). The base material of the ADS was PDMS elastomer (Sylgard 184, Dow Inc.) (base and cross-linker mass ratio 10:1) loaded with neodymium-iron-boron particles (NdFeB, 5 μm, Magquench GmbH). The PDMS to NdFeB mass ratio was 1:3. To fabricate the ADS, the prepolymer was cast onto a flat poly(methyl methacrylate) plate to form a film with the desired thickness. After thermal polymer curing in the hot oven at 90°C for 3 hours, the polymer was demolded and folded (method 1 for visualization.)/wrapped around a nonmagnetic rod (method 2 for 3D visualization.) for magnetization in a VSM (EZ7, Microsense) with 1.8 T. Then, the unfolded polymer was put on the flat plate again and cut by the laser into the desired geometry. Last, the ADS was demolded and ready for assembly.

The base material of the FDS was Dragon Skin 30 polymer matrix (Smooth-On Inc.). To fabricate the FDS, the positive model of the tool, which was used to fabricate the negative PDMS mold (base and cross-linker mass ratio 10:1), was first prepared by lithography using a patterned mask. The base material was cast into the negative PDMS mold and cured at room temperature (23°C) for 24 hours. Then, the sample was demolded and ready for assembly.

To fabricate the C1 and C2, PDMS was cast onto a flat poly plate to form a 0.05 mm thick film. After thermal polymer curing in the hot oven at 90°C for 3 hours, the connection segments were demolded after using the laser to cut them into the desired geometry. The width of the C1 (*w*_C1_) and C2 (*w*_C2_) was 0.28 and 0.10 mm, respectively. The length of C1 (*l*_C1_) and C2 (*l*_C2_) was both 20 mm normally, but they were adjustable when used for various scenarios, e.g., *l*_C1_ changed to 20 cm in the functional demonstrations in physiologically relevant phantoms under x-ray imaging.

After all of those segments were prepared, put all parts together, and used PDMS to connect them, the detailed fabrication process is shown in fig. S1. After assembly, a dimethylacrylamide hydrogel layer (*56*, *72*) was coated on the tool to improve its biocompatibility and hemocompatibility, as well as to reduce friction between the tool and phantoms. For delivery and retraction, the tool was connected to a medical catheter (ID = 0.7112 mm, polyurethane medical tubing, Nordson Medical) using PDMS.

### Preparation of simulants

On the basis of the geometrical features of occlusions and the advantages and limitations in terms of fabrication and materials for vessel-related phantoms (table S4 and S5), we fabricated phantoms with occlusions by three methods: 3D printing, molding technique, and hybrid method.

1) 3D printing. Except for agarose-based phantoms (phantoms P and R), all phantoms with stenosis-like partial occlusions were fabricated by 3D printing (Form 3B, Formlabs Inc.) using ultraviolet (UV) curable resin Clear V4 (Formlabs Inc.) given the feasibility of fast prototyping. Besides, two phantoms (phantoms M and N) with CTO-like severe occlusion containing an MC on the side branch and the phantom with two branches (phantom AA) were also 3D printed.

2) Molding technique. Given the fine sizes and tortuous shapes of MCs with *d*_c_min_ ≤ 0.5 mm, except for the agarose-based phantoms with tortuous MCs (phantom Q), phantoms with side MCs (phantoms M and N), and the phantom with both partial occlusion and severe occlusion (phantom S), most phantoms with severe occlusions were fabricated by the modeling technique. The straight MCs were realized by needles of different sizes (SEIRIN J-Type, SEIRIN Corporation, Japan), and the tortuous MCs were realized by bent copper wires (diameter: 0.2 and 0.5 mm.) as the positive molds. The normal lumen was realized using 3D printed pins as positive molds, which with holes on the sides to fix needles or wires. After assembling the positive molds of MCs and normal lumens in a container, PDMS (mass ratio 10:1) was poured into the container. After PDMS was cured in the hot oven at 90°C for 3 hours, the positive molds were pulled out to get the final phantoms. The agarose-based phantom R was also fabricated using this method. We first configured the positive molds for the channel and the adjustable pins used to tune the locations of the blood clots in a container. Second, we mixed agarose (A9539, Sigma-Aldrich) with water (2.5 wt %) and heated the mixture in the microwave for 1.5 min at 600 W. Third, we poured the agarose-based solution into the container with the assembled simulants and then cooled it in ice for 1 hour. After the phantom was formed, we pulled out all positive molds.

3) Hybrid method. This method was used to prepare agarose-based phantoms with a tortuous route or an MC (phantom P and Q). Because of the complexity of the lumen shape or the tortuosity of the MC, a negative mold for the desired shape was firstly 3D printed (Ultimaker 3, Ultimaker Ltd.) using water-soluble materials (PVA, Ultimaker Ltd.), and then the soft polymer (Ecoflex 0050, Smooth-On Inc.) was injected into the negative mold by the syringe. After the polymer was cured, we put the negative mold in the water to dissolve. After 24 hours, the cured polymer with desired shapes as the positive mold was taken out and ready to mold the channel. The agarose-based solution was poured into a container with the positive mold and then cooled in ice for 1 hour. After the phantom was formed, the positive mold could be pulled out directly.

The detailed fabrication method and material for each phantom were summarized in table S3. To reduce the friction between tools and phantoms, phantoms composed of PDMS and UV-curable resin were coated with a hydrogel layer using the same method for tools.

The blood analog for the quantitative analyses in all phantoms was the glycerol/deionized water mixture, whose viscosity could be regulated by adjusting the ratio of glycerin to deionized water (*73*). For the investigations, the volume ratio of glycerin to deionized water was 44 to 65 and the mixture has a dynamic viscosity of 4.4 cP at room temperature 23°C, matching human blood for normal control subjects and moderate normal artery shear rates at 37°C, i.e., 4.4 ± 0.5 cP (*74*). For the ex vivo demonstrations in vessels, PBS (pH 7.4, Gibco, Thermo Fisher Scientific) was pumped to the porcine coronary arteries to clean the route first, and then the blood analog was used for the tests.

### Experimental test platforms

In the tethered mode, the experimental setup consists of an introducer, a linear positioner, a pulsatile pump, and an actuation magnet (fig. S2). The introducer is a catheter sheath set (Radifocus Introducer II, Terumo Corp., Japan) composed of a side tube with a three-way stop cock and a catheter sheath with a check valve. The medical tubing connects the tool delivered from the catheter sheath. The blood analog, dye, and contrast agent were injected through the three-way stop cock. The linear positioner controlled the forward and backward motion of the medical catheter, which was used for the advancement and retraction of the tool. The blood analog was pumped by a commercial pulsatile blood pump (Harvard Apparatus). The permanent magnet used to actuate the deformation of the ADS was a 50-mm cubic permanent magnet (N45, Implotex GmbH). In characterizations and demonstrations, the magnet’s position along the *y* axis was kept constant because its size was much larger than the occlusion.

In the investigation and demonstration of the 3D visualization, the magnetic field, with a strength of around 50 mT, was provided by a Halbach array. In the demonstration of the steering, the permanent actuator magnet steering tool was a 20-mm cubic magnet (N45).

### Modeling of the deformation of the ADS

We developed a theoretical model to predict the maximum deformation height of the ADS using the pseudo–rigid body (PRB) model and the energy-based approach (*75*). We considered the lumen as rigid boundaries without deformation since experiments showed that phantoms with different properties did not lead to notable differences in the tool’s deformation (fig. S25). The tool was assumed to be always along the *yz* plane. We considered the bending areas of the ADS as the flexible joints and adjacent magnetization segments of the joint to be two magnetic rods with opposite magnetization directions when the ADS is deformed (fig. S26). Besides, we assumed that each joint has one degree of freedom for bending.

Therefore, in the final state, we could know that the elastic energy stored in the ADS was

$$E=\sum_{i=1}^{n-1}\frac{1}{2}E_{\mathrm{ADS}}I_{\mathrm{ADS}}{\left(\frac{\theta_{i}}{l_{\mathrm{joint}}}\right)}^{2}l_{\mathrm{joint}}$$ (3)

where *E*_ADS_ and *I*_ADS_ were Young’s modulus and the area moment of inertia of ADS, respectively. *n* was the count of the magnetic rods, *l*_joint_ was the length of the joint (bending area), and θ_i_ was the strain of the *i*th joints and could be written as

$$\theta_{i}=\alpha_{i+1}-\sum_{j=1}^{i}\alpha_{j}$$ (4)

where α_i_ was the rotation angle of the *i*th rod relative to the *y* axis in the final state.

The magnetic energy of all rods was

$$U=-\sum_{i=1}^{n}({m_{d}}_{i})B(p_{i}^{m})$$ (5)

where ${m_{d}}_{i}$ was the magnetic moment of the *i*th rod, and $B(p_{i}^{m})$ was the magnetic field of the actuation magnet at the middle point of the *i*th rod ($p_{i}^{m}$).

And the work done by the fluid drag during the deformation of the FDS was

$$W=\sum_{n=1}^{i}F_{\mathrm{Drag}}^{i}\;\cdot \;\delta p_{i}^{m}+F_{\mathrm{Drag}}^{\mathrm{FDS}}\;\cdot \;\delta p_{N}^{e}$$ (6)

where the $F_{\mathrm{Drag}}^{i}$ was the fluid drag applied on the *i*th rod, $\delta p_{i}^{m}$ was the displacement of the middle point of the *i*th rod from the initial state to the final state, $F_{\mathrm{Drag}}^{\mathrm{FDS}}$ was the fluid drag applied on the FDS and composed by the fluid drags applied on all beams $F_{\mathrm{Drag}}^{b}$ (fig. S27), and $\delta p_{N}^{e}$ was the displacement of the FDS in the final state.

To obtain the deformation height of the rods, a static equilibrium configuration can be solved by finding the local minimum of the total potential energy

δV=E+U−W (7)

The equilibrium equation can be solved by the fmincon solver in MATLAB (R2021b, MathWorks Inc.), which was used to find the minimum of the constrained nonlinear multivariable function, then θ_i_ and α_i_ could be obtained.

Given the results solved, the maximum deformation height of ADS could be calculated by

$$h_{\max}=l_{m}\cdot \max(\sin|\alpha_{1}|,\sin|\alpha_{2}|,\ldots ,\sin|\alpha_{N}|)$$ (8)

More details on the derivations could be referred to note S2.

### Preparation of porcine coronary arteries and blood clots for ex vivo tests

The porcine hearts and blood were received as an animal side product (registration number: DE 08 111 1008 21). The permit and registration number for the scientific use of animal side products were issued by the office for public order, in particular, the authorities for official food control, consumer protection, and veterinary services of the state capital Stuttgart. As requested by the permit, an official holding register of the biomaterial is kept, and the used animal side products are pressure-sterilized following the experiment.

The porcine coronary arteries for x-ray imaging were cut from the fresh porcine hearts acquired from the animals within 48 hours after they were slaughtered and stored under 5°C during transportation. The cut tissues were cleaned with PBS and then prepared for tests. The blood clots were manually fabricated from blood obtained from freshly slaughtered porcine within 48 hours and stored under 5°C. The liquid with a volume ratio of 50:1 for blood to calcium chloride aqueous solution (0.5 mol/L) was prepared. After 15 min at room temperature (23°C), the thrombi were formulated and then immersed in PBS for the test of morphology visualization. We used 3D-printed pins to immobilize and adjust the location of these clots (fig. S21).

### Statistical analysis

All quantitative values from the experiments were presented as means ± SD. Each quantitative investigation was conducted on at least three tool samples. The three-sample *t* test and one-way ANOVA test were used for the statistical analysis. We set the statistical significance at a 95% confidence level (*P* < 0.05).

## Supplementary Materials

### This PDF file includes:

Notes S1 to S5 Figs. S1 to S28 Tables S1 to S6 Legends for movies S1 to S9 References
