# Supplementary material for: Magnetically assisted soft milli-tools for occluded lumen morphology detection
Source: Sci Adv. 2023 Aug 16;9(33):eadi3979. doi: 10.1126/sciadv.adi3979 (PMC10431716; doi:10.1126/sciadv.adi3979)
Supplement: Supplementary file 1 — Notes S1 to S5 Figs. S1 to S28 Tables S1 to S6 Legends for movies S1 to S9 References [file sciadv.adi3979_sm.pdf]

Science Advances
AAAS

Supplementary Materials for

Magnetically-assisted soft milli-tools for occluded lumen morphology detection

Yingbo Yan *et al.*

Corresponding author: Wenqi Hu, wenqi@is.mpg.de; Metin Sitti, sitti@is.mpg.de

Sci. Adv. **9**, eadi3979 (2023)
DOI: 10.1126/sciadv.adi3979

The PDF file includes:

Notes S1 to S5
Figs. S1 to S28
Tables S1 to S6
Legends for movies S1 to S9
References

Other Supplementary Material for this manuscript includes the following:

Movies S1 to S9

Supplementary Note S1. Physiological features of the possible occlusions

Vascular occlusion can occur in various parts of the body (*76-82*). Here, we used the coronary artery as a reference for the design and fabrication of simulants. The diameter of the coronary artery is in the range of 2 - 4 mm (*83, 84*), and we chose 3.0 mm as the normal diameter d_{n} of the uniform size lumen of the simulants. For the simulants of partial occlusions, we used vascular stenosis as a reference. The partial occluded vessels could be accompanied by complex internal structures, e.g., diffuse stenosis (*77, 85, 86*), which may be caused by the presence of diffused small plaques. We set the length of occlusions l_{o} to 20.0 mm, which is within the range of the stenosis length (*87, 88*). For the simulants of severe occlusions, we chose CTO as a reference and mainly considering the minimum diameter $d_{\mathrm{c\_min}}$ of the internal MC, which is usually less than 0.5 mm and has a cross-sectional area A_{mc} of up to 2.4 mm^2 (*89, 90*). CTO could be on main branches, and on side branches with acute or obtuse bifurcation angles (*91*). MCs inside CTO are complex structures, they could be tortuous and have multiple points extending to various locations, e.g., vessel walls, the distal end of the vessel, and even stopping in the middle (false MC) (*92-95*). In our investigations and demonstrations, we utilized the CTO with the blunt type entrance as the reference, which is more difficult to detect and access than the tapered entrance clinically (*96-99*).

The minimum allowed distance between the magnet and the lumen $l_{\mathrm{z\_min}}$ varies for different organs, tissues, and imaging views given the physical constraints (*63-65*). For example, the distance between the skin and the heart varies depending on the imaging view used, e.g., 32.1 ± 7.9 mm for parasternal, 31.3 ± 11.3 mm for apical, and 70.8 ± 22.3 mm for subcostal (*65*). In our investigation, we investigated the effects of the distance l_{z} between the magnet and the lumen on the tool's performance in the range of 20 mm to 140 mm, and in demonstrations, we maintained the distance l_{z} in the range of 40 to 100 mm. For visualizing the morphology of partial occlusion, we set the imaging parameters of the X-ray to 50 kV and 65 μA, which are consistent with the range of parameters used for medical X-ray imaging (*100-104*). In the clinic, the injection speed of the medical contrast agent is in the range of 0.5 - 5.0 ml/s (*105-107*). In demonstrations, we maintained the injection speed of the contrast agent simulant, i.e., dissolved food dye, and medical contrast agent in the range of 0.4 - 1.2 ml/s.

There is also a correlation between the flow rate Q in narrowed vessels and the degree of occlusions. Q of the partial occlusion, i.e., mild, moderate, and severe stenosis, is usually in the range of 10.0 – 70.0 ml/min and basically decreases as the grade of the stenosis increases (*62, 84*).

While for severe occlusions, i.e., CTO, the Q is no more than 10.0 ml/min (*90, 108*). Considering the body healthy state, the viscosity η of the blood could be changed in the range of 4 – 45 cP (*109*), we investigated the performance of the tool in the range of 0.9 – 50.0 cP. The average resting heart rate for adults ranges from 60 to 100 bpm, and we unified the rate to 80 bpm. We used a commercial pulsatile blood pump (Harvard Apparatus) to pump blood analog with 80 bpm in all the experimental conditions with the flow.

Supplementary Note S2. Modeling of the deformation of the ADS

Here, we utilized the pseudo-rigid-body (PRB) model and the energy-based approach to model and solve the kinematics of the active deformation segment (ADS) with multi-mode deformation (*75*). For a PRB model, it was assumed that the rod comprises N rigid links jointed by N-1 flexible joints. In this study, as all large bending deformation of the ABS occurred in bending sections as shown in **fig. S26**, we considered the bending sections of ADS as the flexible joints and adjacent magnetization segments of the joint to be two magnetic rods with opposite magnetization directions. Besides, we assumed that each joint has one degree of freedom for bending, which ignored the extension, shear, and twisting of the rod. Thus, rod kinematics could be described as the poses of the discrete links along with the arc length.

1.1. Elastic energy *E*

As shown in **fig. S26**, the length of the joint could be calculated by:

$$l_{\text{joint}} = \pi \cdot \ t_{\text{ADS}}, \tag{1}$$

where t_{ADS} was the thickness of ADS (in z direction).

The rotation angle of a single rod relative to y axis could be described by the angle α_{i}, which means that we could know the strain θ_{i} of the ith joints, as follows:

$$\theta_{\text{i}} = \alpha_{\text{i+1}} - \sum_{n=1}^{i} \alpha_{\text{n}}. \tag{2}$$

Therefore, in the final state, we could know the elastic energy stored in the ADS was

$$E = \sum_{i=1}^{n-1} \frac{1}{2} E_{\mathrm{ADS}} I_{\mathrm{ADS}} \left(\frac{\theta_{\mathrm{i}}}{l_{\mathrm{joint}}} \right)^2 l_{\mathrm{joint}}, \tag{3}$$

where E_{ADS} and I_{ADS} were Young's modulus and the area moment of inertia of ADS, respectively.

1.2. Magnetic energy *U*

As we know the deformation of a single rod relative to the y axis, we could know the direction vector for the ith rod was

$$\boldsymbol{r}_{\mathrm{i}}^{\mathrm{r}} = (\cos(\alpha_{\mathrm{i}}), \sin(\alpha_{\mathrm{i}})).$$

Furthermore, the vector from the zero point to the end point of the ith rod was

$$\boldsymbol{p}_{\mathrm{i}}^{\mathrm{e}} = l_{\mathrm{m}} \left(\sum_{n=1}^{i} \boldsymbol{r}_{\mathrm{n}}^{\mathrm{r}} \right), \tag{4}$$

where l_{m} was the length of the rod, which was equal to the length of one magnetization segment of the ADS. Therefore, we could know the location of the end point of the ith rod, which was $p_{\mathrm{i}}^{\mathrm{e}}$. And the vector from the zero point to the middle point of the ith rod was:

$$\boldsymbol{p}_{\mathrm{i}}^{\mathrm{m}} = \boldsymbol{p}_{\mathrm{i}}^{\mathrm{r}} - \frac{l}{2} \boldsymbol{r}_{\mathrm{i}}^{\mathrm{r}}, \tag{5}$$

Therefore, we could know the location of the middle point of the ith rod, which was $p_{\mathrm{i}}^{\mathrm{m}}$.

At the same time, as we know the direction vector for the ith magnetized section, we could know the dipole moment $\boldsymbol{m}_{\mathrm{d_i}}$ for the ith rod was:

$$\boldsymbol{m}_{\mathrm{d_i}} = m_{\mathrm{d}} \boldsymbol{r}_{\mathrm{i}}^{\mathrm{r}}, \tag{6}$$

where m_{d} was the magnetic moment of one magnetization segment of the ADS. Since we know the location of the actuator magnet $p^{\mathrm{a}}\ (y_{\mathrm{mag}}, z_{\mathrm{mag}})$, the magnetic energy of all rods in the point-dipole field could be calculated by:

$$U = -\sum_{i=1}^{n} (\boldsymbol{m}_{d_{\mathrm{i}}}) \boldsymbol{B}(p_{\mathrm{i}}^{\mathrm{m}}), \tag{7}$$

where $\boldsymbol{B}(p_{\mathrm{i}}^{\mathrm{m}})$ was the magnetic field of the actuation magnet at the middle point of the ith rod ($p_{\mathrm{i}}^{\mathrm{m}}$). As the cubic actuator magnet (50 mm) was much larger than the ADS, we considered the magnetic field B of the actuator magnet to be uniform in the y direction and only change in the z direction. We measured the magnetic field B of the actuator magnet in different positions with a Gaussian meter, as shown in **table S6**.

1.3. Work done by the fluid drag *W*

We considered the blood analogue as the laminar flow and W came from drag forces applied to the FDS and ADS. As we know the flow rate Q pumped into the inlet, we could know the average velocity of the blood analog in the lumen was:

$$v_{\mathrm{avg}} = \frac{Q}{A_{\mathrm{lumen}}}, \tag{8}$$

where A_{lumen} was the cross-section area of the lumen in the xz plane as shown in **fig. S27**. Moreover, the velocity profile of the blood analogue relative to the beams of the FDS in xz plane could be calculated by (*110*):

$$u_{(\mathrm{x},\mathrm{z})} = 2(v_{\mathrm{avg}} - v_{\mathrm{d}})\left(1 - \frac{(x^2 + z^2)}{R^2}\right), \tag{9}$$

where R was the radius of the lumen, v_{d} was the delivery speed of the tool.

We considered the tool always in the central area of the xy plane as it's a symmetric structure, and the length of the intermediate beam of the FDS in the x direction was 0 as it's much smaller than the length l_{b} (in the x direction) of the beams. The location of the FDS in the xz plane was $(0, z_0)$. Therefore, for one beam with the width w_{b} (in the z direction), the drag force applied on it in the y direction was:

$$F_{\mathrm{Drag}}^{\mathrm{b}} = \int_0^{l_{\mathrm{b}}} \int_{-\frac{w_{\mathrm{b}}}{2} - z_0}^{\frac{w_{\mathrm{b}}}{2} - z_0} \frac{1}{2} \rho_{\mathrm{b}} u_{(\mathrm{x},\mathrm{z})}^2 c_{\mathrm{d}} \mathrm{d}x \mathrm{d}z\,, \tag{10}$$

where ρ_{b} was the mass density of the blood analog and c_{d} was the drag coefficient. When the beam was placed vertically in low Reynolds number (Re) flow, the drag coefficient c_{d} changes rapidly with the flow speed changes (*111-113*), and given the results of (*112*), we could calculate drag coefficient c_{d} by:

$$c_{\mathrm{d}} = 5.7889Re^{-0.546}, \tag{11}$$

and

$$Re = \frac{\rho_{\mathrm{b}} v_{\mathrm{avg}}(2R)}{\mu}, \tag{12}$$

where μ was the viscosity of the blood analog, R was the radius of the lumen.

Therefore, the flow drag applied on FDS $\boldsymbol{F}_{\mathrm{Drag}}^{\mathrm{FDS}}$ was $(10 \cdot F_{\mathrm{Drag}}^{\mathrm{b}}, 0)$.

When the ADS was placed in the lumen and deformed by the actuator magnet, we assumed that the fluid drag $\boldsymbol{F}_{\mathrm{Drag}}^{\mathrm{i}}(F_{\mathrm{Drag}}^{\mathrm{i}}, 0)$ applied on the rods of the ADS remains constant, and the tool always in the central area of the lumen. Therefore, the fluid drag applied on the ith rod was:

$$F_{\mathrm{Drag}}^{\mathrm{i}} = \frac{1}{2}\rho_{\mathrm{b}} v^2 c_{\mathrm{d}} A'. \tag{13}$$

Here, we considered $v = 2 \cdot \left(v_{\mathrm{avg}} - v_{\mathrm{d}}\right)$ to be the maximum flow speed of the blood analogue in the lumen, as the rod, whose width was much less than the diameter of the lumen, was in the central area of the lumen. And A' was the reference area of the rod in the lumen in the final state and could be calculated by

$$A' = w_{\mathrm{ADS}} \cdot l_{\mathrm{m}} \cdot \sin(\alpha_{\mathrm{i}}), \tag{14}$$

where w_{ADS} was the width of ADS.

As we know the location of the rods, for the ith rod, we could know the displacement of the middle point of it in the final state, which was:

$$\delta \boldsymbol{p}_{\mathrm{i}}^{\mathrm{m}} = \boldsymbol{p}_{\mathrm{i}}^{\mathrm{m}} - \boldsymbol{p}_{\mathrm{i}}^{\mathrm{m_0}}, \tag{15}$$

where $p_{\mathrm{i}}^{\mathrm{m_0}}$ was the position of the middle point for the ith rod in the initial state. Also, we could know the displacement of the flow drag-driven segment (FDS) in the final state

$$\delta \boldsymbol{p}_{\mathrm{N}}^{\mathrm{e}} = \boldsymbol{p}_{\mathrm{N}}^{\mathrm{e}} - \boldsymbol{p}_{\mathrm{N}}^{\mathrm{e_0}}, \tag{16}$$

where $p_{\mathrm{N}}^{\mathrm{e_0}}$ and $p_{\mathrm{N}}^{\mathrm{e}}$ was the position of the end point of the last rod in the initial and final states, respectively.

Furthermore, the work done by the fluid drag during the deformation was

$$W = \sum_{n=1}^{i} \boldsymbol{F}_{\mathrm{Drag}}^{\mathrm{i}} \cdot \delta \boldsymbol{p}_{\mathrm{i}}^{\mathrm{m}} + \boldsymbol{F}_{\mathrm{Drag}}^{\mathrm{FDS}} \cdot \delta \boldsymbol{p}_{\mathrm{N}}^{\mathrm{e}}, \tag{17}$$

Since we have known the elastic energy E and magnetic energy U of the deformed ADS in the final state and the work W done by the fluid drag applied on the tool. In order to obtain the rotation angle α_{i}, a static equilibrium configuration could be solved by finding the local minimum of the total potential energy

$$\delta V = E + U - W. \tag{18}$$

The equilibrium equation could be solved by the fmincon solver in MATLAB (R2021b, MathWorks, Inc.), which was used to find the minimum of the constrained nonlinear multivariable function. Given the results solved, the maximum deformation height of ADS could be calculated by:

$$h_{\mathrm{max}} = l_m \cdot max(h_1, h_2, \dots, h_{\mathrm{N}}), \tag{19}$$

where h_{i} was the deformation height for the ith rod and could be calculated by

$$h_{\mathrm{i}} = sin|\alpha_{\mathrm{i}}|. \tag{20}$$

Supplementary Note S3. Floating and advancement of the tool

Here, we considered the tool to be rigid and composed of ADS, FDS, connection segment 1 (C1), and connection segment 2 (C2). When the tool was in the lumen, as the existence of gravity, buoyancy and fluid drag, there were three torques, generated by gravity ($\boldsymbol{T}_{\mathrm{g}}$), buoyancy ($\boldsymbol{T}_{\mathrm{b}}$), and fluid drag ($\boldsymbol{T}_{\mathrm{drag}}$), applied on the tool as shown in **fig. S28**. Those torques could lead to the rotation of the tool around the connection point p_{c} in the xz plane.

According to the experimental observation, the failure of advancement was caused by the buckling of the tool in contact with the luminal wall. Therefore, for successfully advancing and floating in occlusions, a torque

$$\boldsymbol{T}_{\mathrm{total}} = \boldsymbol{T}_{\mathrm{b}} + \boldsymbol{T}_{\mathrm{g}} + \boldsymbol{T}_{\mathrm{Drag}}, \tag{21}$$

was required to guide the tool rotating toward the center of the lumen to avoid contact with the wall.

We considered the FDS as a mass point. Given the geometric constraints (**fig. S27**), the initial position of FDS in the xz plane could be calculated by

$$x_0 = 0,$$

$$z_0 = -\left(R^2 - l_{\mathrm{b}}^2\right)^{\frac{1}{2}}.$$

2.1. Torque ($\boldsymbol{T}_{\mathrm{g}}$) generated by gravity

T_{g} was generated from four parts, which was the gravity of the C1 ($\boldsymbol{G}^{\mathrm{C1}}$), ADS ($\boldsymbol{G}^{\mathrm{ADS}}$), C2 ($\boldsymbol{G}^{\mathrm{C2}}$), and FDS ($\boldsymbol{G}^{\mathrm{FDS}}$), respectively.

The torque generated by the FDS was

$$T_{\mathrm{g}}^{\mathrm{FDS}} = \rho_{\mathrm{FDS}}\, g V_{\mathrm{FDS}} \cdot (l_{\mathrm{c1}} + l_{\mathrm{ADS}} + l_{\mathrm{c2}}) \cdot cos\theta, \tag{22}$$

where ρ_{FDS} and V_{FDS} were the mass density and volume of the FDS, respectively. g was the gravitational acceleration. θ was the angle between the tool and the y direction in the initial state and could be calculated by

$$sin\,\theta = \frac{-z_0}{l_{\mathrm{c1}} + l_{\mathrm{ADS}} + l_{\mathrm{c2}}}. \tag{23}$$

Besides, the torques, generated by the gravity, on the ADS, C1, C2 was

$$T_{\mathrm{g}}^{\mathrm{C1}} = \int_0^{l_{\mathrm{c1}}} \rho_{\mathrm{PDMS}} \cdot t_{\mathrm{c1}} \cdot w_{\mathrm{c1}} \cdot \mathrm{d}l \cdot g \cdot l\, cos\theta\,, \tag{24}$$

$$T_{\mathrm{g}}^{\mathrm{ADS}} = \int_{l_{\mathrm{c1}}}^{l_{\mathrm{c1}}+l_{\mathrm{ADS}}} \rho_{\mathrm{ADS}} \cdot t_{\mathrm{ADS}} \cdot w_{\mathrm{ADS}} \cdot \mathrm{d}l \cdot g \cdot l\, cos\theta, \tag{25}$$

$$T_{\mathrm{g}}^{\mathrm{C2}} = \int_{l_{\mathrm{c1}}+l_{\mathrm{ADS}}}^{l_{\mathrm{c1}}+l_{\mathrm{ADS}}+l_{\mathrm{c2}}} \rho_{\mathrm{PDMS}} \cdot t_{\mathrm{c2}} \cdot w_{\mathrm{c2}} \cdot \mathrm{d}l \cdot g \cdot l\, cos\theta, \tag{26}$$

respectively. Here, ρ_{PDMS} and ρ_{ADS} was the mass density of PDMS and ADS, respectively.

Thus, we could know that the torque generated by gravity and applied on the tool was:

$$T_{\mathrm{g}} = T_{\mathrm{g}}^{\mathrm{C1}} + T_{\mathrm{g}}^{\mathrm{ADS}} + T_{\mathrm{g}}^{\mathrm{C2}} + T_{\mathrm{g}}^{\mathrm{FDS}}, \tag{27}$$

and the direction of T_{g} was clockwise.

2.2. Torque ($\boldsymbol{T}_{\mathbf{b}}$) generated by buoyancy

As the four parts of the tool were under buoyancy in the fluid, T_{b} also was generated from four parts, which was

$$T_{\mathrm{b}}^{\mathrm{C1}}=\int_{0}^{l_{\mathrm{c1}}}\rho_{\mathrm{b}}\cdot t_{\mathrm{c1}}\cdot w_{\mathrm{c1}}\cdot \mathrm{d}l\cdot g\cdot l\,cos\theta, \tag{28}$$

$$T_{\mathrm{b}}^{\mathrm{ADS}}=\int_{l_{\mathrm{c1}}}^{l_{\mathrm{c1}}+l_{\mathrm{ADS}}}\rho_{\mathrm{b}}\cdot t_{\mathrm{ADS}}\cdot w_{\mathrm{ADS}}\cdot \mathrm{d}l\cdot g\cdot l\,cos\theta, \tag{29}$$

$$T_{\mathrm{b}}^{\mathrm{C2}}=\int_{l_{\mathrm{c1}}+l_{\mathrm{ADS}}}^{l_{\mathrm{c1}}+l_{\mathrm{ADS}}+l_{\mathrm{c2}}}\rho_{\mathrm{b}}\cdot t_{\mathrm{c2}}\cdot w_{\mathrm{c2}}\cdot \mathrm{d}l\cdot g\cdot l\,cos\theta, \tag{30}$$

$$T_{\mathrm{b}}^{\mathrm{FDS}}=\rho_{\mathrm{b}}gV_{\mathrm{FDS}}\cdot(l_{\mathrm{c1}}+l_{\mathrm{ADS}}+l_{\mathrm{c2}})\cdot cos\theta, \qquad (31),$$

respectively.

Thus, we could know that the torque generated by the buoyancy was:

$$T_{\mathrm{b}}=T_{\mathrm{b}}^{\mathrm{C1}}+T_{\mathrm{b}}^{\mathrm{ADS}}+T_{\mathrm{b}}^{\mathrm{C2}}+T_{\mathrm{b}}^{\mathrm{FDS}}, \tag{32}$$

and the direction of T_{b} was counterclockwise.

2.3. Torque ($\boldsymbol{T}_{\mathbf{Drag}}$) generated by the drag force

When the lumen existed the flow, there would be a drag force parallel to the flow direction applied on the tool.

The drag applied on the C1, ADS, C2 and FDS was

$$F_{\mathrm{Drag}}^{\mathrm{C1}}=\int_{0}^{l_{\mathrm{C1}}\cdot sin\theta}\int_{-\frac{D_{\mathrm{c1}}}{2}}^{\frac{D_{\mathrm{c1}}}{2}}\frac{1}{2}\rho_{\mathrm{b}}*u_{(\mathrm{x},\mathrm{z})}*c_{\mathrm{d}}\mathrm{d}x\mathrm{d}z\,, \tag{33}$$

$$F_{\mathrm{Drag}}^{\mathrm{ADS}}=\int_{l_{\mathrm{C1}}\cdot sin\theta}^{(l_{\mathrm{c1}}+l_{\mathrm{ADS}})\cdot sin\theta}\int_{-\frac{D_{\mathrm{c}}}{2}}^{\frac{D_{\mathrm{c}}}{2}}\frac{1}{2}\rho_{\mathrm{b}}*u_{(\mathrm{x},\mathrm{z})}*c_{\mathrm{d}}\mathrm{d}x\mathrm{d}z, \tag{34}$$

$$F_{\mathrm{Drag}}^{\mathrm{C1}}=\int_{(l_{\mathrm{c1}}+l_{\mathrm{ADS}})\cdot sin\theta}^{(l_{\mathrm{c1}}+l_{\mathrm{ADS}}+l_{\mathrm{C2}})\cdot sin\theta}\int_{-\frac{D_{\mathrm{c1}}}{2}}^{\frac{D_{\mathrm{c1}}}{2}}\frac{1}{2}\rho_{\mathrm{b}}*u_{(\mathrm{x},\mathrm{z})}*c_{\mathrm{d}}\mathrm{d}x\mathrm{d}z, \tag{35}$$

$$F_{\mathrm{Drag}}^{\mathrm{FDS}}=10\cdot F_{\mathrm{drag}}, \tag{36}$$

respectively.

Furthermore, the torque generated by the drag force applied on four parts was

$$T_{\mathrm{Drag}}^{\mathrm{C1}} = \int_{0}^{l_{\mathrm{c1}}} F_{\mathrm{Drag}}^{\mathrm{C1}} \cdot \mathrm{d}l \cdot cos\theta, \tag{37}$$

$$T_{\mathrm{Drag}}^{\mathrm{ADS}} = \int_{l_{\mathrm{c1}}}^{l_{\mathrm{c1}}+l_{\mathrm{ADS}}} F_{\mathrm{Drag}}^{\mathrm{ADS}} \cdot \mathrm{d}l \cdot cos\theta, \tag{38}$$

$$T_{\mathrm{Drag}}^{\mathrm{C2}} = \int_{l_{\mathrm{c1}}+l_{\mathrm{ADS}}}^{l_{\mathrm{c1}}+l_{\mathrm{ADS}}+l_{\mathrm{c2}}} F_{\mathrm{Drag}}^{\mathrm{C2}} \cdot \mathrm{d}l \cdot cos\theta, \tag{39}$$

$$T_{\mathrm{Drag}}^{\mathrm{FDS}} = 10F_{\mathrm{drag}} \cdot (l_{\mathrm{c1}} + l_{\mathrm{ADS}} + l_{\mathrm{c2}}) \cdot sin\theta, \tag{40}$$

respectively.

Thus, the torque generated by the fluid drag and applied on the tool was

$$T_{\mathrm{Drag}} = T_{\mathrm{Drag}}^{\mathrm{C1}} + T_{\mathrm{Drag}}^{\mathrm{ADS}} + T_{\mathrm{Drag}}^{\mathrm{C2}} + T_{\mathrm{Drag}}^{\mathrm{FDS}}, \tag{41}$$

and the direction of T_{Drag} was counterclockwise.

In our tests, for successful floating and advancement, the rotation direction of the tool should be counterclockwise. Hence, if the total torque $\boldsymbol{T}_{\mathrm{total}}$ applied on the tool was written as

$$T_{\mathrm{total}} = T_{\mathrm{b}} - T_{\mathrm{g}} + T_{\mathrm{Drag}}. \tag{42}$$

the total torque applied on the tool should satisfy

$$T_{\mathrm{total}} > 0. \tag{43}$$

Since T_{g} and T_{b} were decided by the design of the tool, T_{Drag} was related to the flow rate Q and delivery speed v_{d}. Therefore, we could define a threshold of the torque

$$T_t(Q, v_{\mathrm{d}}) = T_{\mathrm{g}} - T_{\mathrm{b}}. \tag{44}$$

Here, if

$$T_{\mathrm{t}} \geq 0, \tag{45}$$

the tool could float in the fluid even if the flow rate Q in the lumen was 0. And when the flow rate Q > 0, there would be a drag force applied on FDS in the flow direction, we could consider that

the tool could realize the advancement in the lumen. Else, the flow rate Q in the lumen and delivery speed v_{d} of the tool should satisfy the relation of

$$T_{\mathrm{Drag}} > T_t(Q, v_{\mathrm{d}}). \tag{46}$$

Supplementary Note S4. Biocompatibility and hemocompatibility of the tool

The current prototype was fabricated with three types of materials: NdFeB microparticles, PDMS, and Dragon Skin™ 30 polymer matrix. NdFeB is not biocompatible and hemocompatible due to its cytotoxicity and corrosive properties. To improve the biocompatibility, i.e., avoid the corrosion and cytotoxicity, of the NdFeB (*114*), the microparticles could be coated with various biocompatible materials such as silica (*56*), polyethylene glycol or dextran.

PDMS and Dragon Skin™ 30 polymer matrix have good safety and biocompatibility, which have been proven *in vivo* (*58, 115*). However, PMDS is not hemocompatible due to the fast adsorption of proteins and relatively high adhesion of blood platelets, which would induce thrombosis (*116*). To improve the hemocompatibility of PDMS, surface modifications can be carried out to reduce its protein adsorption and platelet adhesion.

To further enhance the safety, i.e., biocompatibility and hemocompatibility of the tool, we coated a layer of dimethylacrylamide (DMAA) hydrogel on its surface. DMAA-based hydrogel has been shown to have low levels of hemolysis, platelet adhesion, and minimal activation of coagulation and complement, making it a suitable choice for blood-contact applications. However, further research and validation are needed to ensure its safety in practical applications, particularly in the *in vivo* environment (*56*).

Supplementary Note S5. Safety interaction with vessels

When the ADS was deformed by the magnet and the tool was retracted, there were two groups of interaction forces, i.e., the radial forces ($F_{\mathrm{r}}^{\mathrm{b\_wall}}$ and $F_{\mathrm{r}}^{\mathrm{u\_wall}}$), and the axial forces ($F_{\mathrm{f}}^{\mathrm{b\_wall}}$ and $F_{\mathrm{f}}^{\mathrm{u\_wall}}$), applied on the wall of the lumen, as shown in **fig. S23A**.

Actuated by the magnet, the deformed ADS would contact the wall of the lumen on several contact points. We assumed that all external forces are uniformly distributed on each magnetized

segment of deformed ADS and selected one of the magnetized segments for analysis. When the wall was compressed by the deformed ADS, 1) the radial forces from two sources: the magnetic force and magnetic torque applied to the magnetization segments of the deformed ADS. For each magnetization segment, within the range of the distance l_z between the magnet and ADS we investigated (20 – 100 mm), the radial action force due to the magnetic force F_m was no more than 2×10^{-9} N (**fig. S8**), and radial action force $F_{T_m}^{a}$ due to the magnetic torque T_m was no more than 8×10^{-9} N (**fig. S23B**); 2) axial forces from two sources: the fluid drag applied to the tool and T_m applied to the ADS. At each magnetization segment of the ADS, the applied drag force F_{Drag}^{S}, i.e., one quarter of the total drag force applied to the tool, was in the range of 4×10^{-6} N – 3.5×10^{-5} N when the flow rate (Q) was in the range of 0 – 70.0 ml/min (**fig. S23C**). And the force in the flow direction generated from T_m was no more than 8×10^{-9} N (**fig. S23B**). In summary, when the tool was retracted in the lumen, the radial forces applied on the wall of the lumen ($F_a^{b\_wall}$ and $F_a^{u\_wall}$) were no more than 1×10^{-8} N, and the axial forces applied on the wall ($F_f^{b\_wall}$ and $F_f^{u\_wall}$) could be considered no more than 3.5×10^{-5} N. Both of two type interaction forces were much smaller than the forces (~ 10^{-1} N) generated by the medical catheter–phantom interaction (*54*) (**fig. S23D**).

Besides, given the forces we calculated and the contact area we measured (around 1.4×10^{-8} m^2), the maximum compressive stress and maximum shear stress applied on the wall of the lumen were 0.74 Pa and 2.5 kPa, respectively, both were within the range of previous investigations on the contact pressure of soft magnetic robots moving in a tubular structure (*54*, *59*), smaller than the rupture threshold of tissue and plaques on atherosclerotic blood vessels at around 190 kPa (*117*, *118*) and the rupture threshold of endothelial cell at around 12.4 kPa (*54*) **(fig. S23E**).

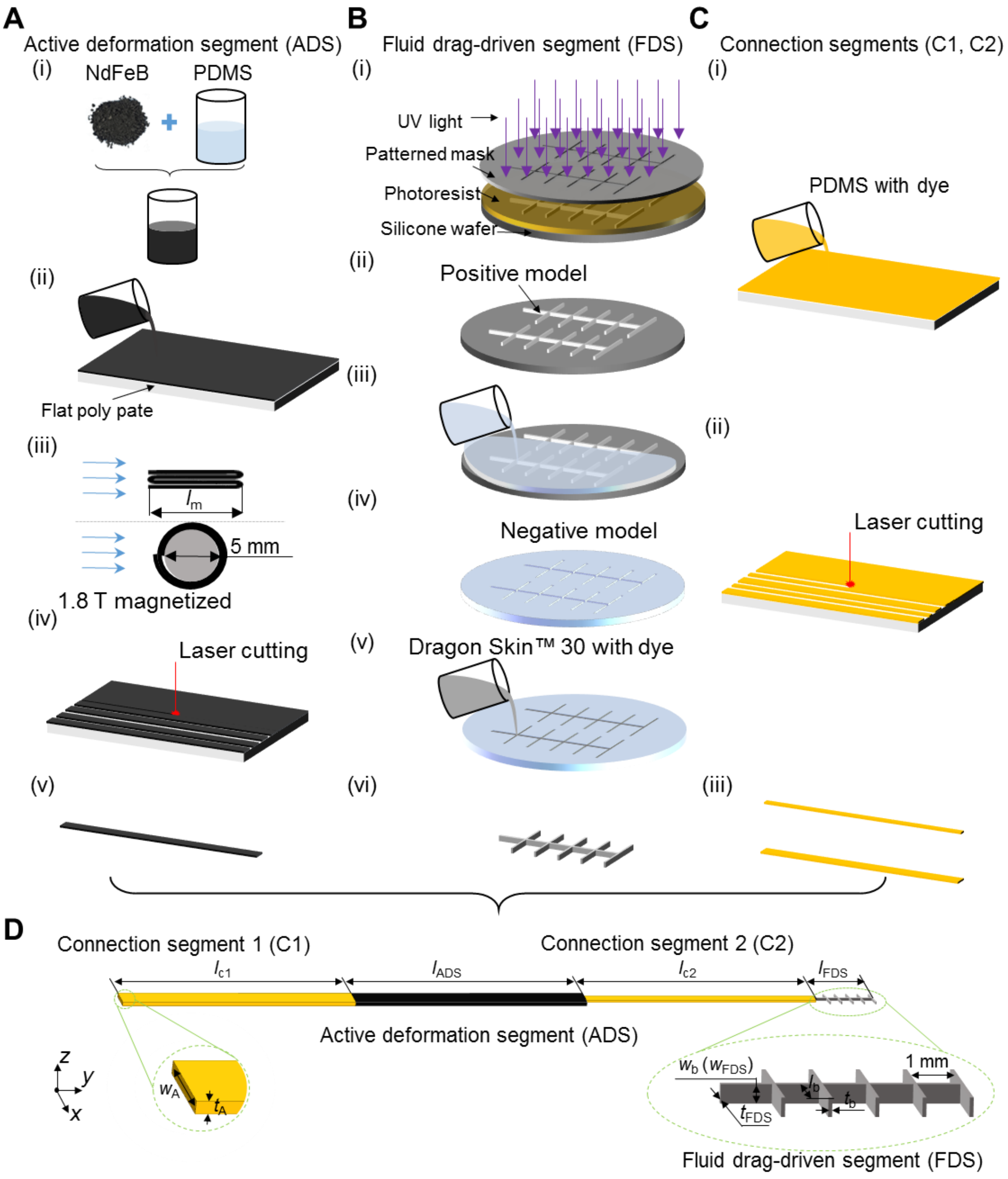

Fig. S1. Fabrication details of the tool. (**A**) Fabrication and magnetization of the active deformation segments (ADS). We used two magnetization methods for morphology visualization. (**B**) Fabrication of the fluid drag-driven segment (FDS). (**C**) Fabrication of the connection segments (C1, C2). (**D**) Final assembly of the complete tool.

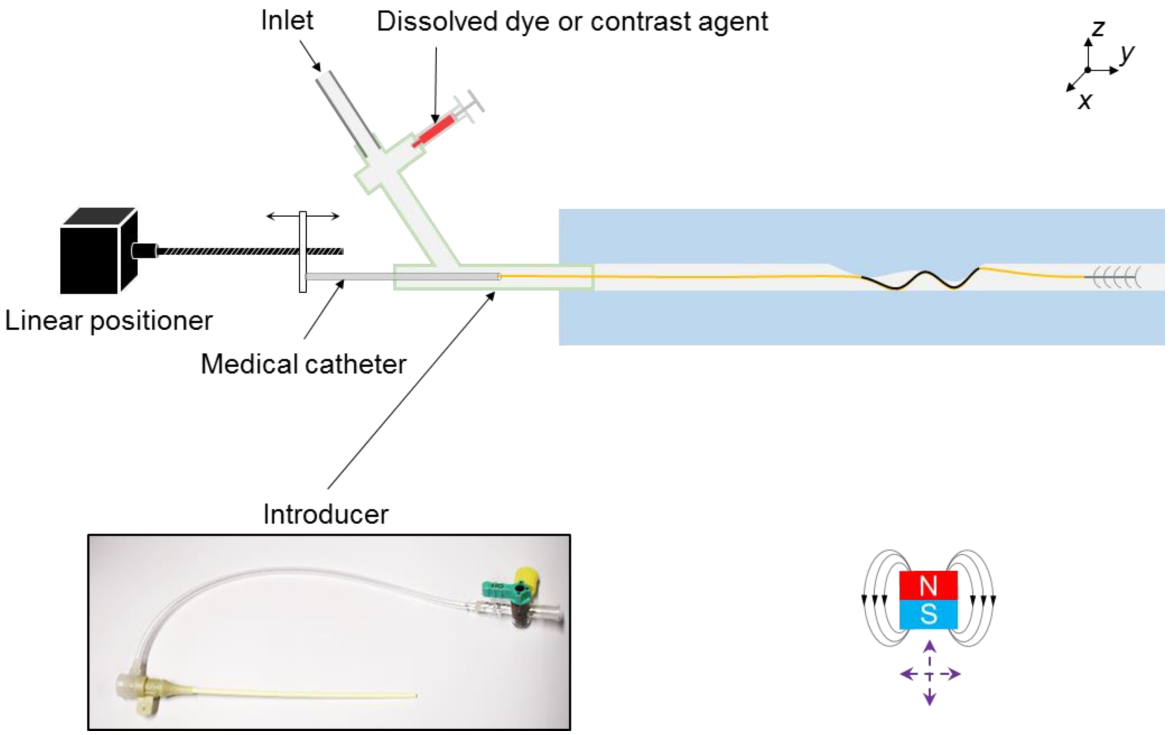

Fig. S2. Experimental setup. The tool was proximally connected to a medical tubing, and the position of the tool was controlled by the linear positioner. An introducer was used to insert the medical tubing and inject the pulsatile blood analog, dyes, and contrast agent. The position of the actuation magnet was controlled manually or through an X-Y stage.

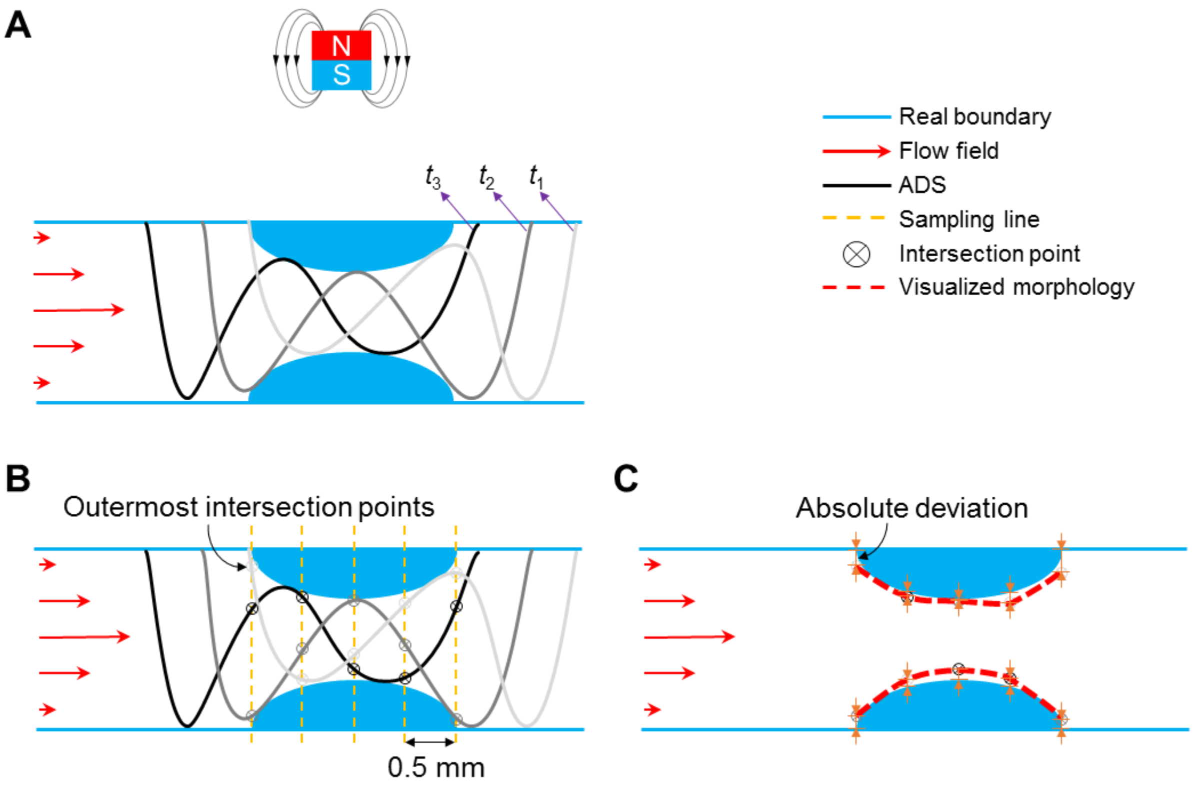

Fig. S3. Computation of the visualization error E_{avg}. (**A**) ADS at different times during retraction. (B) Intersection points on collective body profiles of ADS. **(C)** Absolute deviation of the outermost intersection points from the ground truth. E_{avg} was the average of absolute deviations for all intersection points.

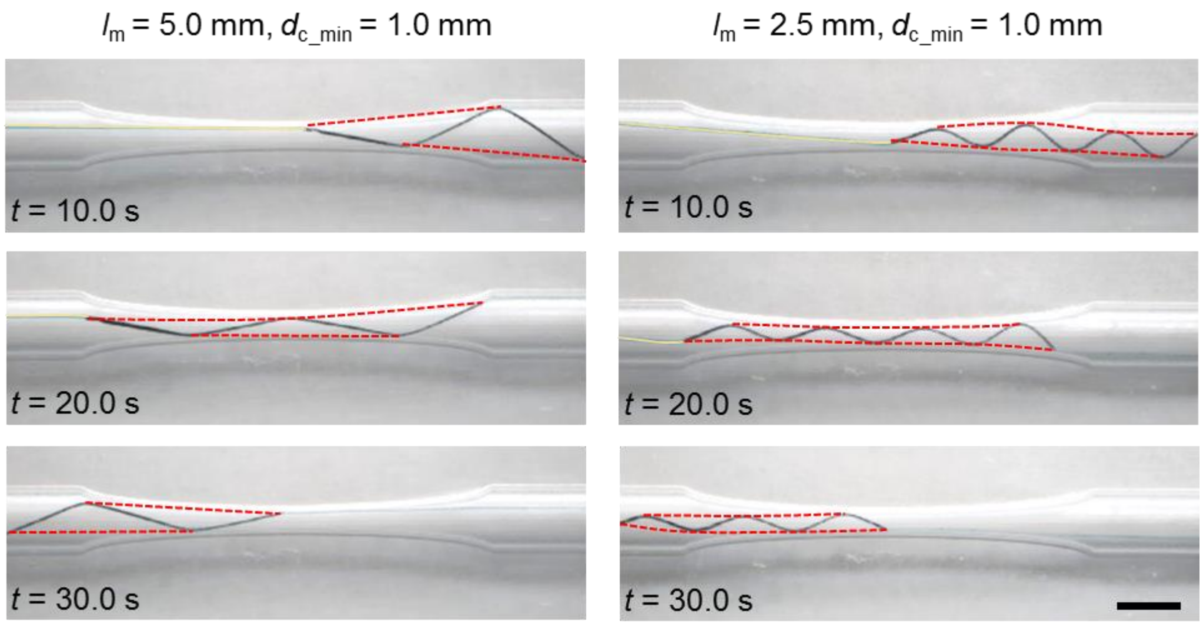

Fig. S4. Deformation of the tool with different lengths l_m of the magnetization segment during the retraction. In phantom B, with the minimum diameter of the channel inside the occlusion d_{c_min} was 1.0 mm, the tool with l_m = 5.0 mm could almost contact the luminal boundary everywhere and hence had a small visualization error (E_{avg} = 0.09 ± 0.003 mm). In contrast, the tool with l_m = 2.5 mm had difficulty in contacting all the luminal boundaries and therefore had a more notable visualization error (E_{avg} = 0.25 ± 0.026 mm). In all investigations, the flow rate Q of the blood analog pumped into the phantom was set to around 10.0 ml/min, the retraction speed v_r was set to 1.0 mm/s. The distance between the magnet and the lumen l_z was set to 50 mm. Scale bar: 3 mm.

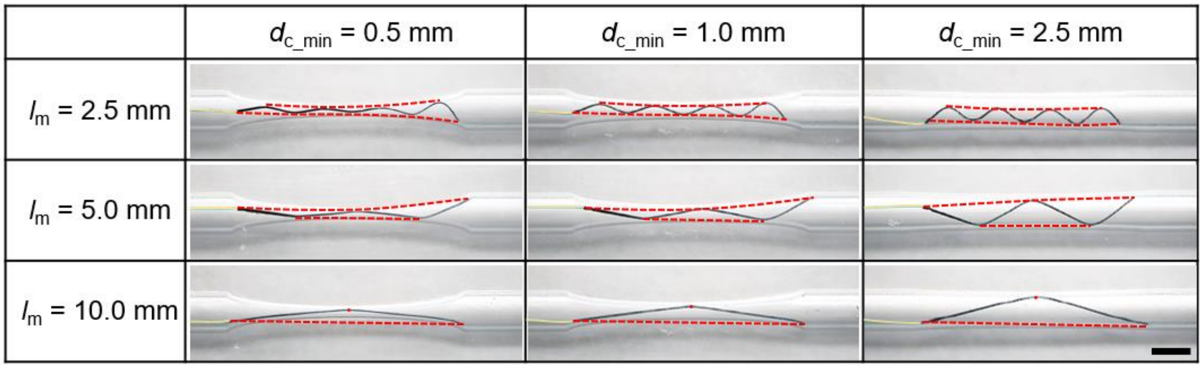

Fig. S5. Comparison of deformed active deformation segments (ADSs) at the same locations in various occlusions. The deformed ADS with l_m = 2.5 mm was hard to contact all lumen borders, especially when d_{c_min} = 2.5 mm (phantom E). In contrast, the deformed ADS with l_m = 5.0 mm and l_m = 10.0 mm both could contact the borders of the lumen even if d_{c_min} = 2.5 mm. However, the former design has more contact points, which may be useful for the operator's real-time detection. Therefore, we chose the tool with length l_m = 5.0 mm for further investigations. In all investigations, the flow rate Q of the blood analog pumped into the phantom was set to around 10.0 ml/min, the retraction speed v_r was set to 1.0 mm/s. The distance between the magnet and the lumen l_z was set to 50 mm. Scale bar: 3 mm.

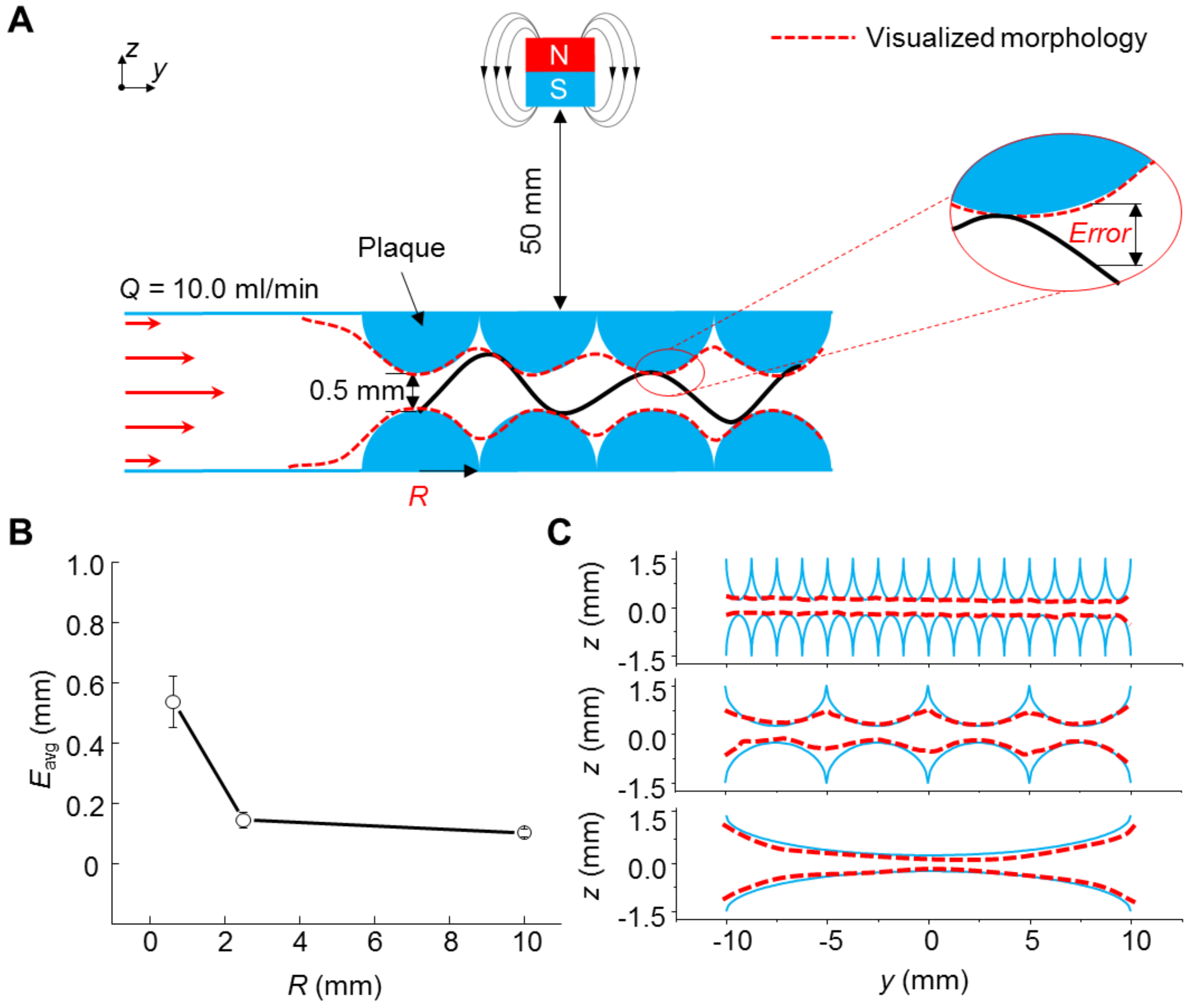

Fig. S6. Investigation on the morphology visualization of the complex occlusions (phantom A, T - U). (**A**) Schematics of the visualization and illustration of the occlusion (d_{c_min} = 0.5 mm) with some plaques. The cross-section of those plaques were standard ellipses, and the radius of the plaque in the flow direction was R. (**B**) Visualization error E_{avg} for different complex occlusions using the same tool. (**C**) Comparison of the lumen visualized by our approach with the ground truth. In all investigations, the length of the magnetization segment l_m of the tool was 5.0 mm, and the retraction speed v_r was set to 1.0 mm/s. For all phantoms, the length of the occlusion (in the y direction) l_o was 20.0 mm. The flow rate Q of the blood analog pumped into the phantom was set to around 10.0 ml/min. Error bars represent standard error (SD) (N = 5).

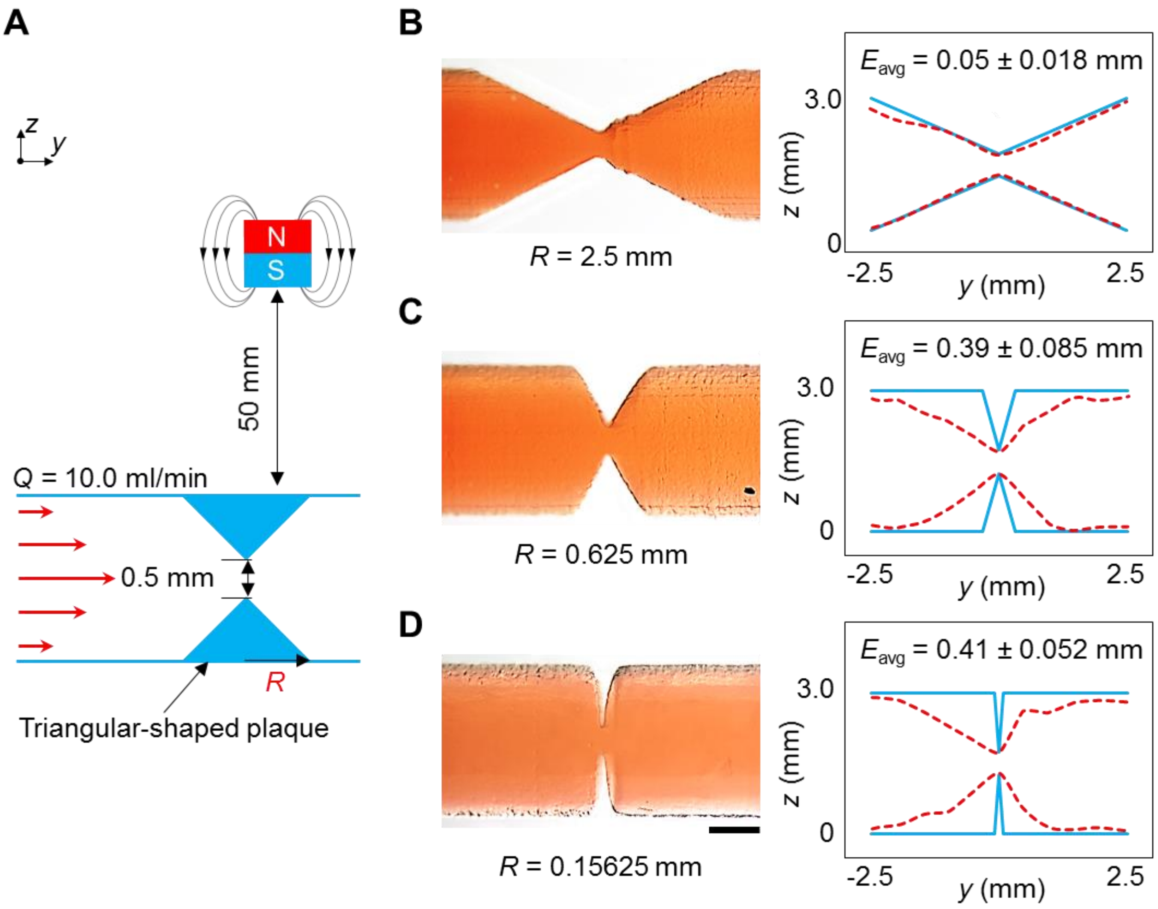

Fig. S7. Investigation on the morphology visualization of occlusions with a triangular-shaped plaque in extreme cases. (**A**) Schematics of the visualization and illustration of the occlusion (d_{c_min} = 0.5 mm). (**B - D**) Investigation results in phantom V - X. We visualized the morphology of the plaque and its surrounding area (length of the occluded area l_o = 5.0 mm). The length of the magnetization segment l_m of the tool was 5.0 mm, and the retraction speed v_r was set to 1.0 mm/s. The flow rate Q of the blood analog pumped into the phantom was set to around 10.0 ml/min. Scale bar: 1 mm.

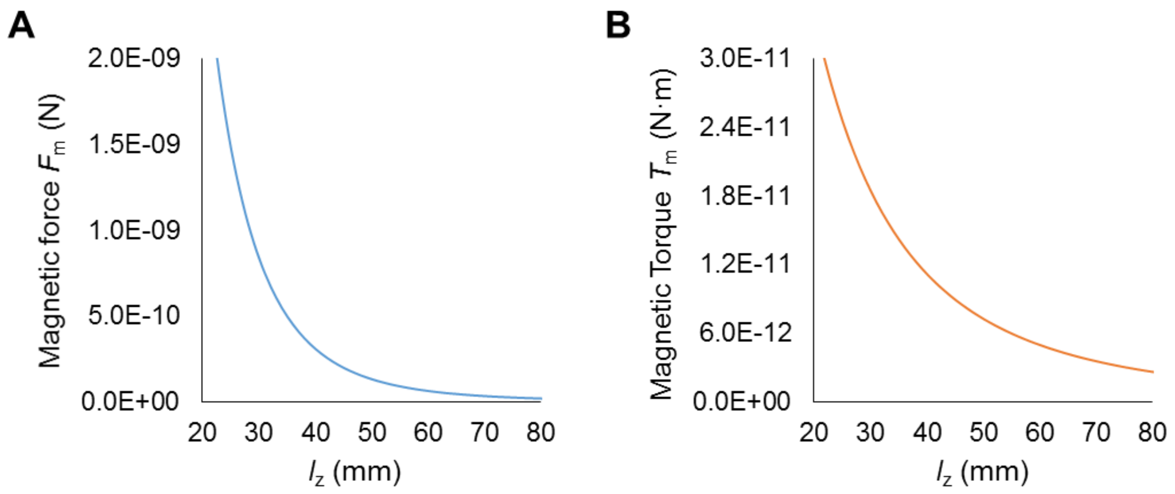

Fig. S8. Effects of the distance between the magnet and the lumen l_z on the magnetic force F_m and magnetic torque T_m applied on the ADS. Both F_m and T_m decreased with the increase of the l_z. However, the change in F_m showed a faster decay with increasing l_z, e.g., when l_z increased from 30 mm to 50 mm, T_m decreased by 60%, while F_m decreased by 84.0%, indicating that F_m showed a smaller influence on the deformation of the ADS in the range of distance l_z (20 – 140 mm) we investigated. The results were calculated by the magnetic dipole model on one magnetization segment of ADS.

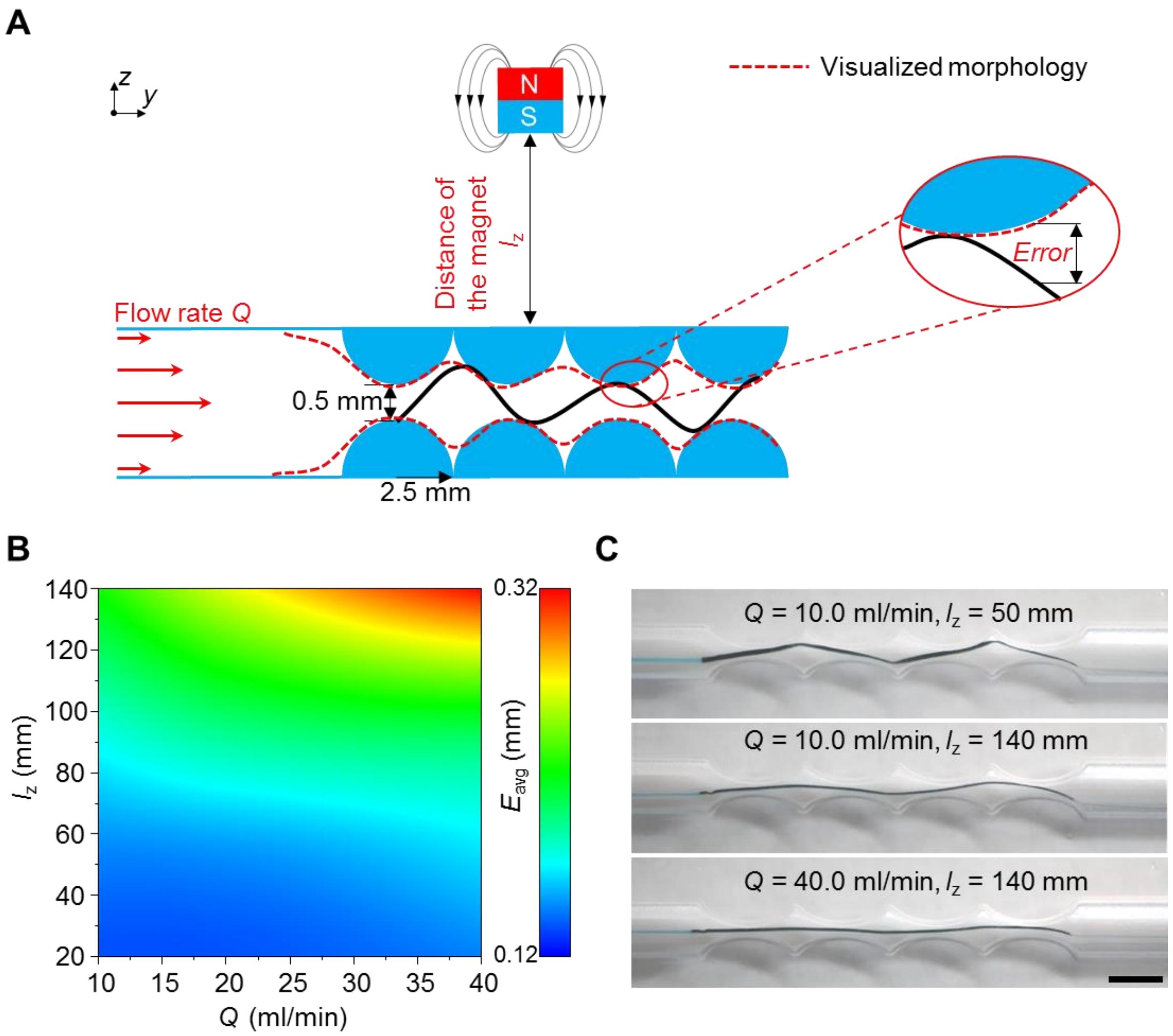

Fig. S9. Investigation on the workable conditions for the tool visualizing a complex occlusion (phantom T). (**A**) Schematics of the visualization. The work conditions include the flow rate of the blood analog Q and the distance between the magnet and the lumen l_z. The minimal diameter of the channel in occlusion d_{c_min} was 0.5 mm, and the radius of the axis in y direction R of plaques was 2.5 mm. (**B**) Investigation results. (**C**) Deformation of the tool in different conditions. In all investigations, the length of the magnetization segment l_m of the tool was 5.0 mm, and the retraction speed v_r was set to 1.0 mm/s. Scale bar: 3 mm.

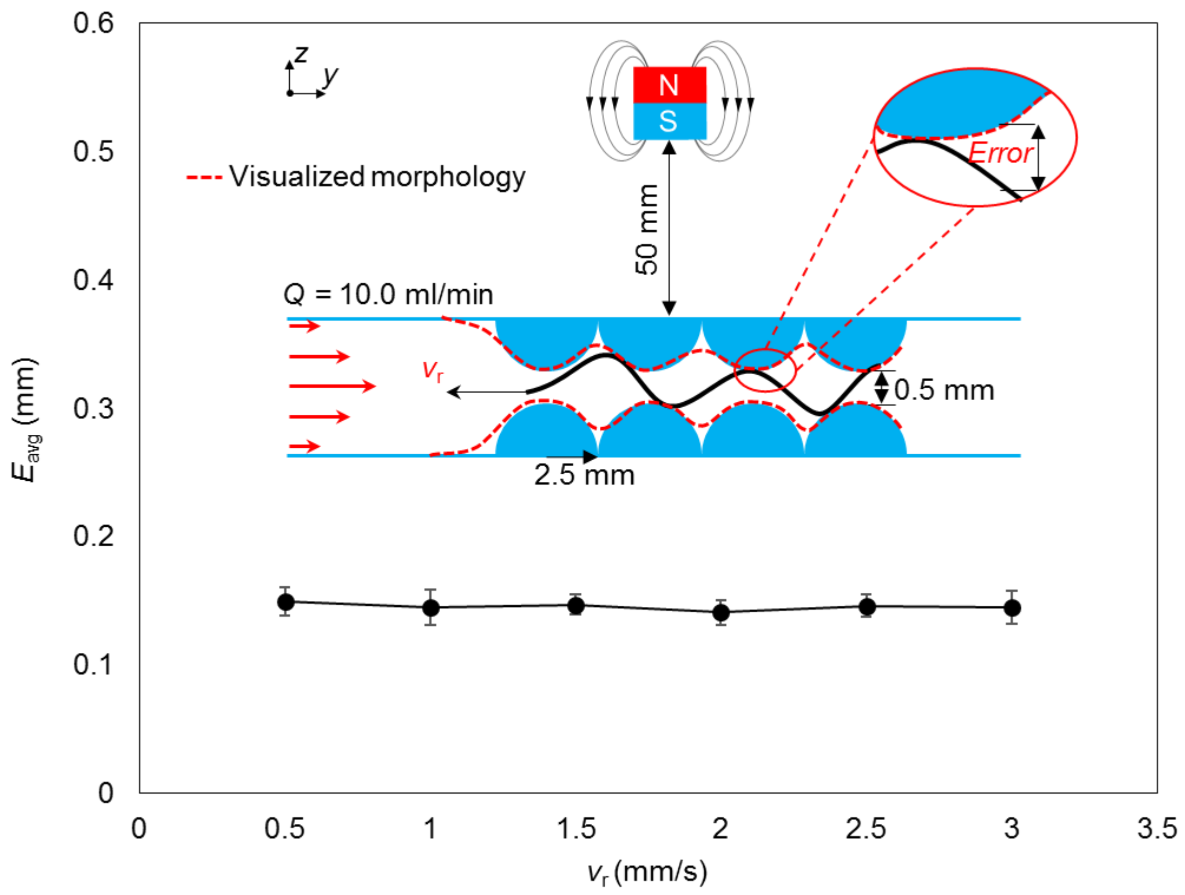

Fig. S10. Influence of the retraction speed v_r on the visualization error E_{avg}. The investigation was performed in the phantom T**.** The minimum diameter of the channel inside occlusion d_{c_min} was 0.5 mm, the radius of the axis in *y* direction *R* was 2.5 mm, and the length of magnetization segment l_m of the tool was 5.0 mm. The flow rate *Q* of the blood analog pumped into the phantom was set to around 10.0 ml/min. Error bars represent standard error (*SD*) (*N* = 5).

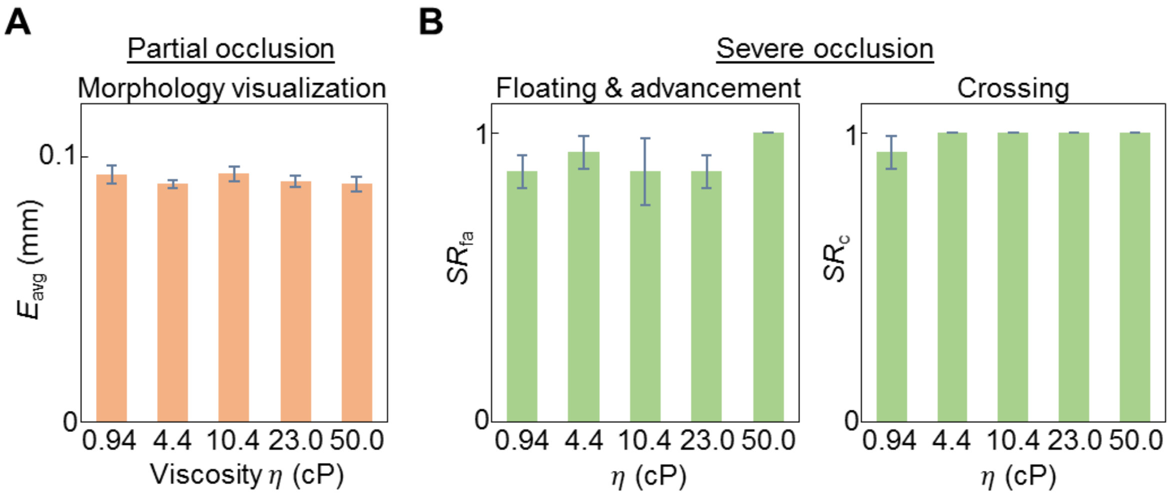

Fig. S11. Effects of fluidic viscosity (η) on the tool's performance. (**A**) Effects of η on the morphology visualization in phantom A. (**B**) Effects of η on the success rate of floating and advancement (SR_{fa}) of the tool advancing in the normal-sized lumen and the success rate (SR_c) of the tool crossing the microchannel in phantom I. Q = 10.0 ml/min in (A) and 3.6 - 5.0 ml/min in (B), v_r = 1.0 mm/s, l_z = 50 mm in (A), v_d = 0.5 mm/s in (B), and l_b = 0.6 mm. Error bars represent SD (N = 5 in (A) and N = 9 in (B)).

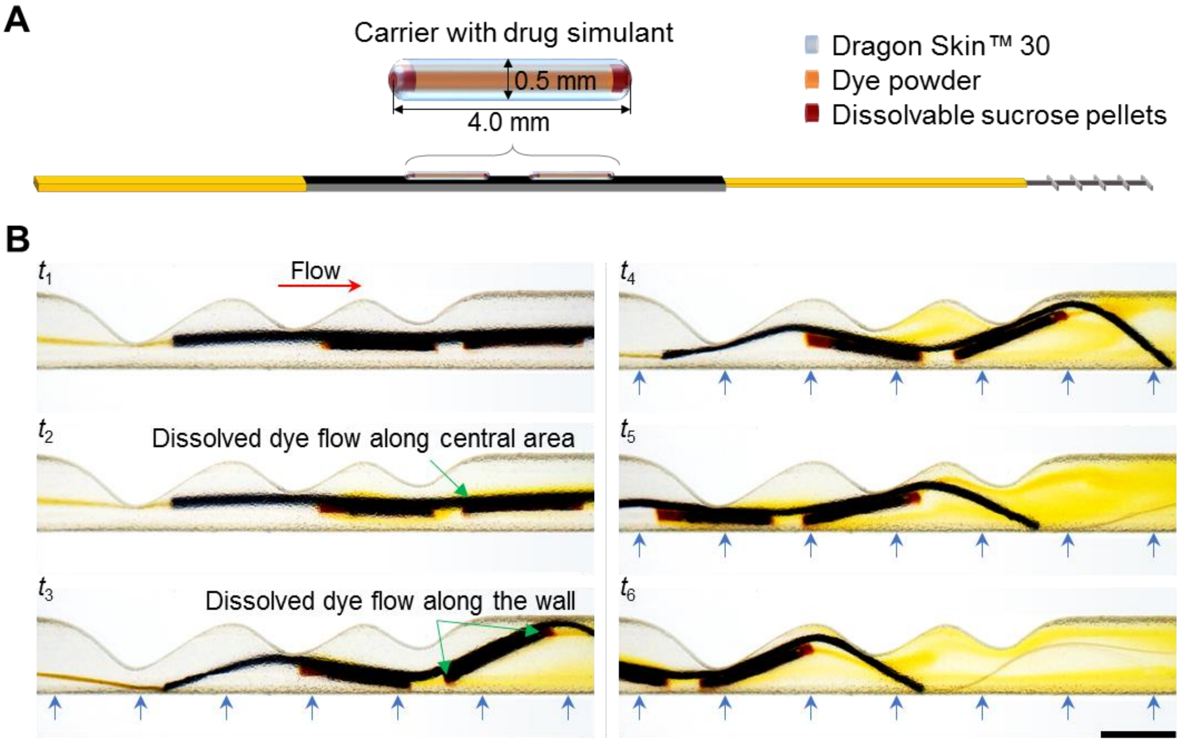

Fig. S12. Drug release at occlusions. (**A**) Schematics of the tool with drug carriers. The drug was simulated by the fluorescein dye powder (Fluorescein sodium, Fisher Scientific U.K. Limited). The carrier was a tubular structure made by Dragon Skin™ 30 and sealed with dissolvable sucrose pellets at two ends. The pellets could be fabricated by other degradable materials, e.g., polycaprolactone (PCL). The on-demand drug release can be realized by other wireless actuation mechanisms, such as heat (*54*, *119*) and magnetic field (*41*, *52*). (**B**) Release of drug simulant on the luminal wall in phantom G. After the deformation of ADS, the tool can help the drug simulant better distribute to the vicinity of occlusions ($t_3 – t_6$) rather than the center of the lumen (t_2). l_z = 50 mm, w_{ADS} = 0.5 mm. Scale bar: 3 mm.

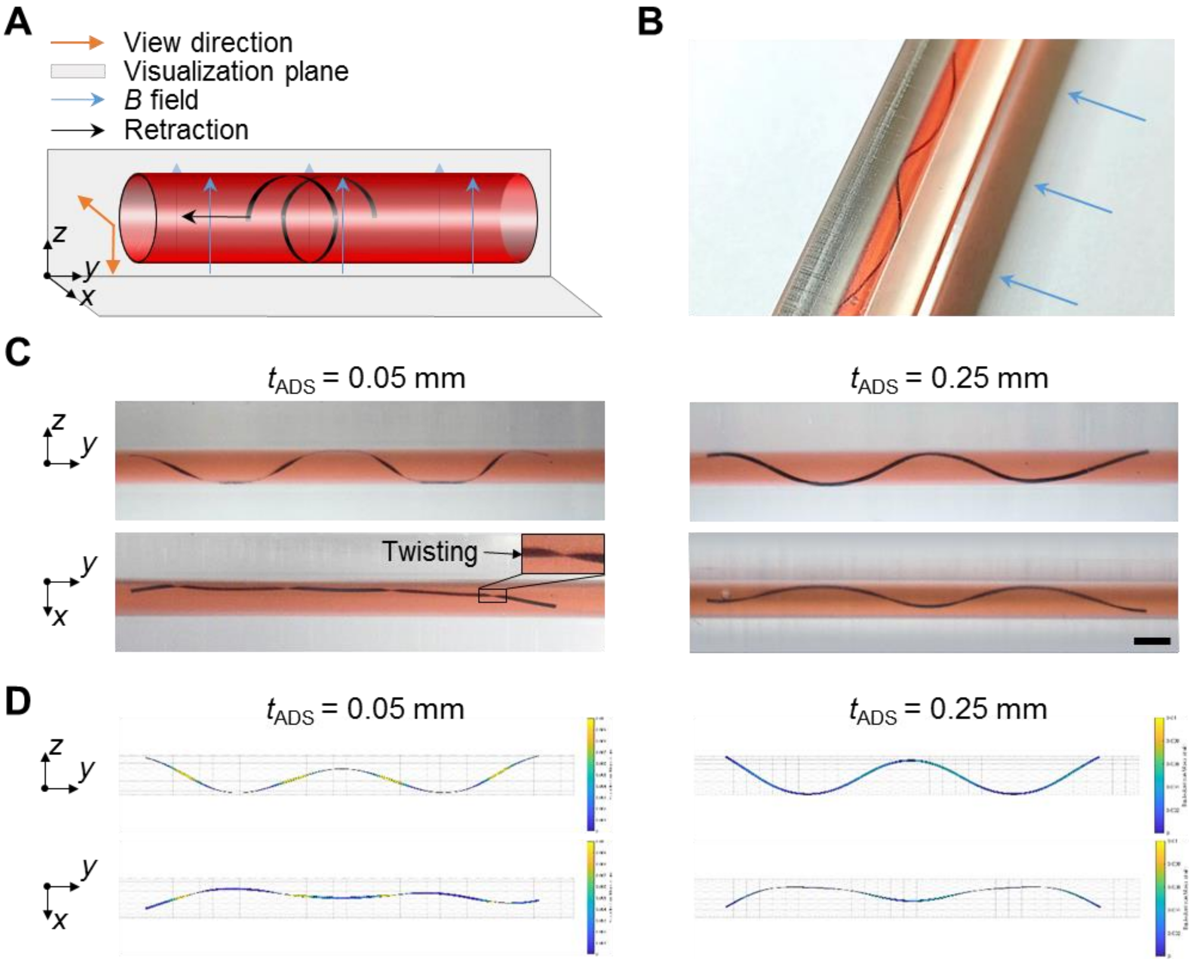

Fig. S13. Effects of the thickness t_{ADS} on helical deformation of the ADS in method 2 of 3D visualization. (**A**) Schematics for the method 2. (**B**) Snapshots of the helix-shaped ADS in the phantom F. (**C**) Snapshots of the deformed ADS in front (*yz* plane) and top (*xy* plane) views during investigation. (**D**) Snapshots of the simulation results. Actuated by the uniform magnetic field (with the strength of around 50 mT) provided by a Halbach array, the ADS generated severe local twists when t_{ADS} = 0.05 mm, while this phenomenon could be improved by increasing t_{ADS}. Scale bar: 3 mm.

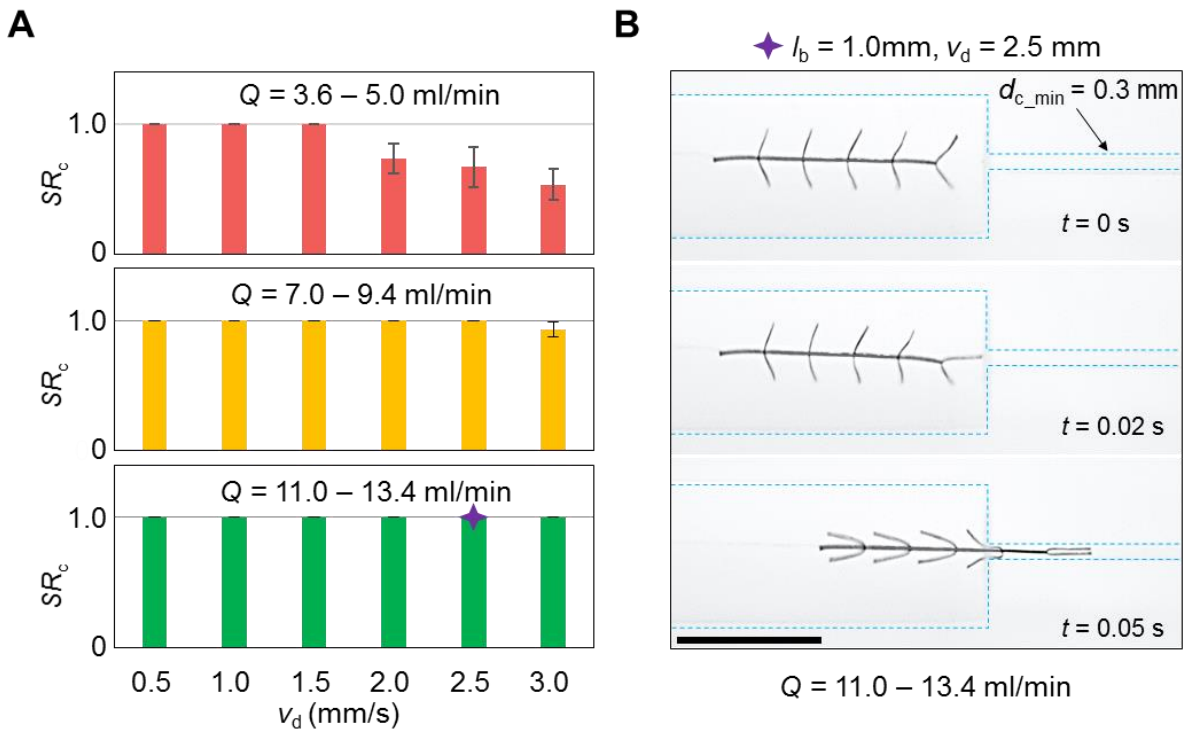

Fig. S14. Investigation on the success rate of crossing (*SR*c) with various flow rates (*Q*) and delivery speeds (*v*d). (**A**) Effects of *Q* and v_d on SR_c. (**B**). One typical case of FDS crossing the MC. Length of beams l_b = 1.0 mm. Error bars represent *SD* (N = 9). Scale bar: 3 mm.

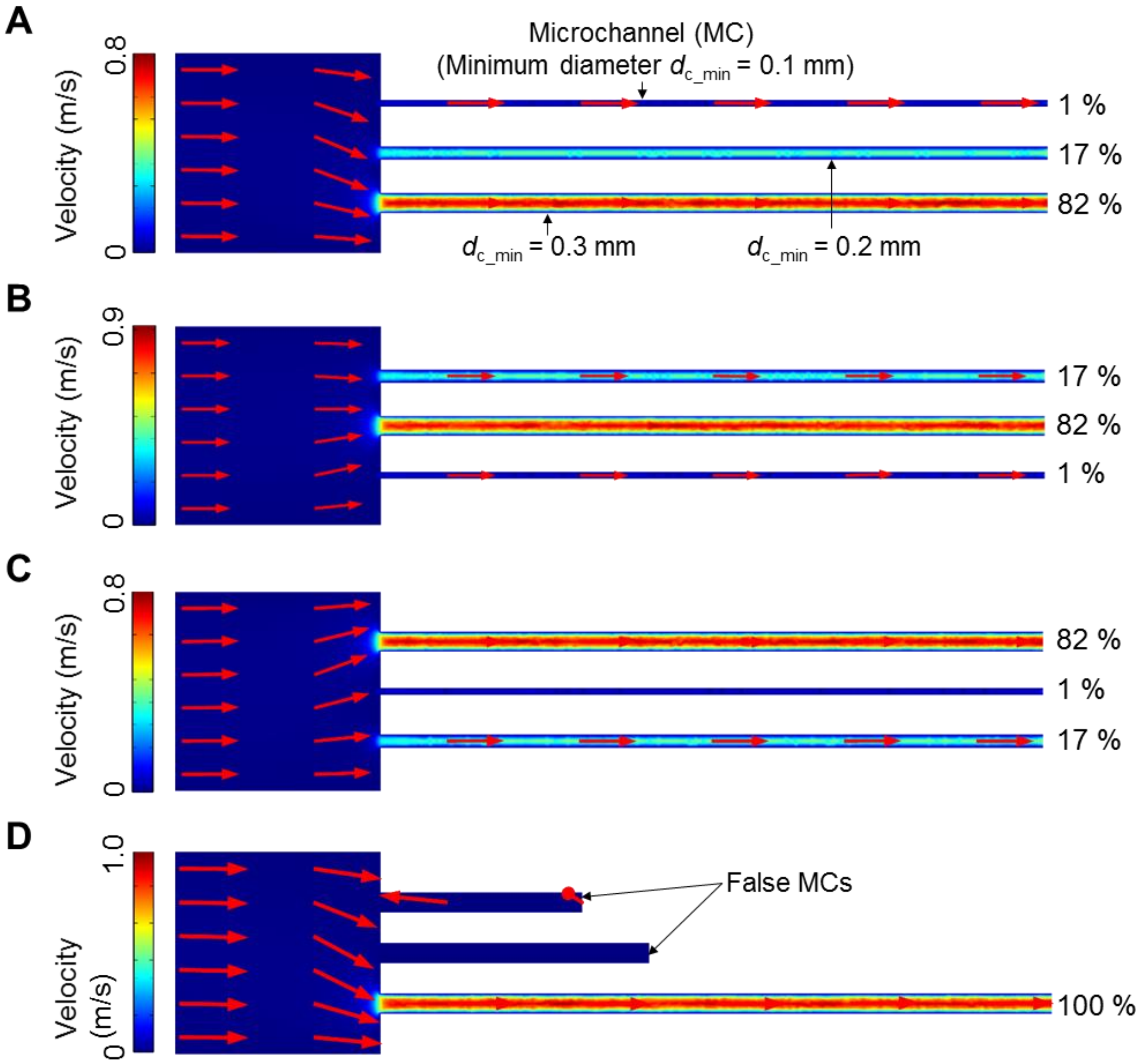

Fig. S15. Quantification of the flow distribution in various severe occlusions. (**A - C**) Flow rate distribution in MCs with various sizes. The minimum diameter of MCs d_{c_min} from top to bottom in three phantoms was 0.1 mm, 0.2 mm, 0.3 mm (phantom J), 0.2 mm, 0.3 mm, 0.1mm (phantom Y), and 0.3 mm, 0.1 mm, 0.2 mm (phantom Z), respectively. Most of the flow rate (82 %) was distributed to the MC with the largest d_{c_min} = 0.3 mm. (**D**) Flow rate distribution in the severe occlusion containing false MCs (phantom K). All the flow rate was distributed to the true MC. The red arrows represent the flow direction. The flow rate Q was set to 4.0 ml/min for all simulations.

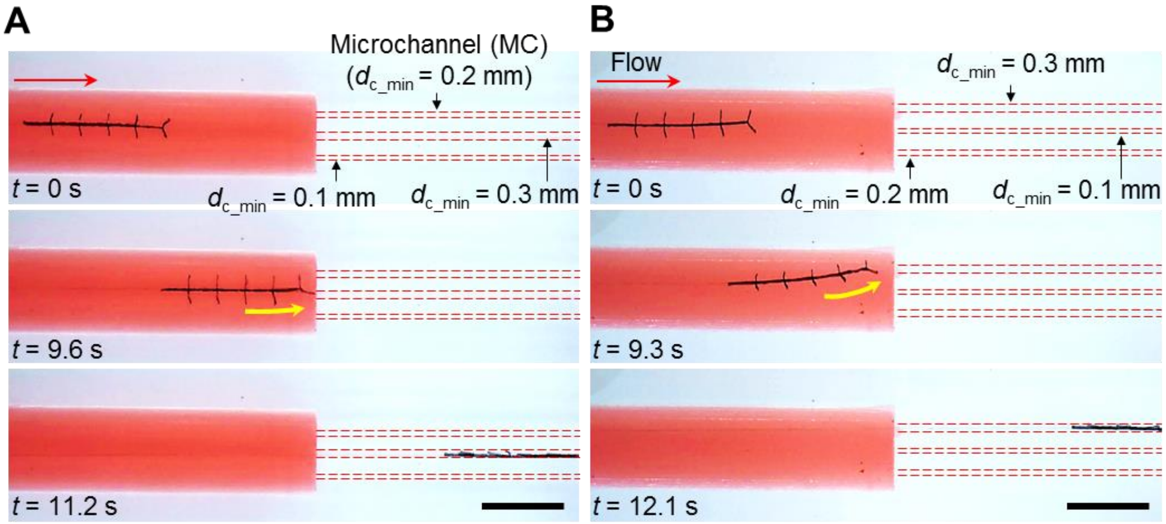

Fig. S16. Demonstrations of crossing microchannel (MC) with the highest flow rates. Snapshots of the tool crossing MCs, where the minimum diameter of MC d_{c_min} from top to bottom was 0.2 mm, 0.3 mm, and 0.1mm, respectively (phantom Y) in (**A**). d_{c_min} from top to bottom was 0.3 mm, 0.1 mm, and 0.2 mm, respectively (phantom Z) in (**B**). In two phantoms, the tool crossed the MC with the largest d_{c_min} = 0.3 mm. All scale bars: 3 mm.

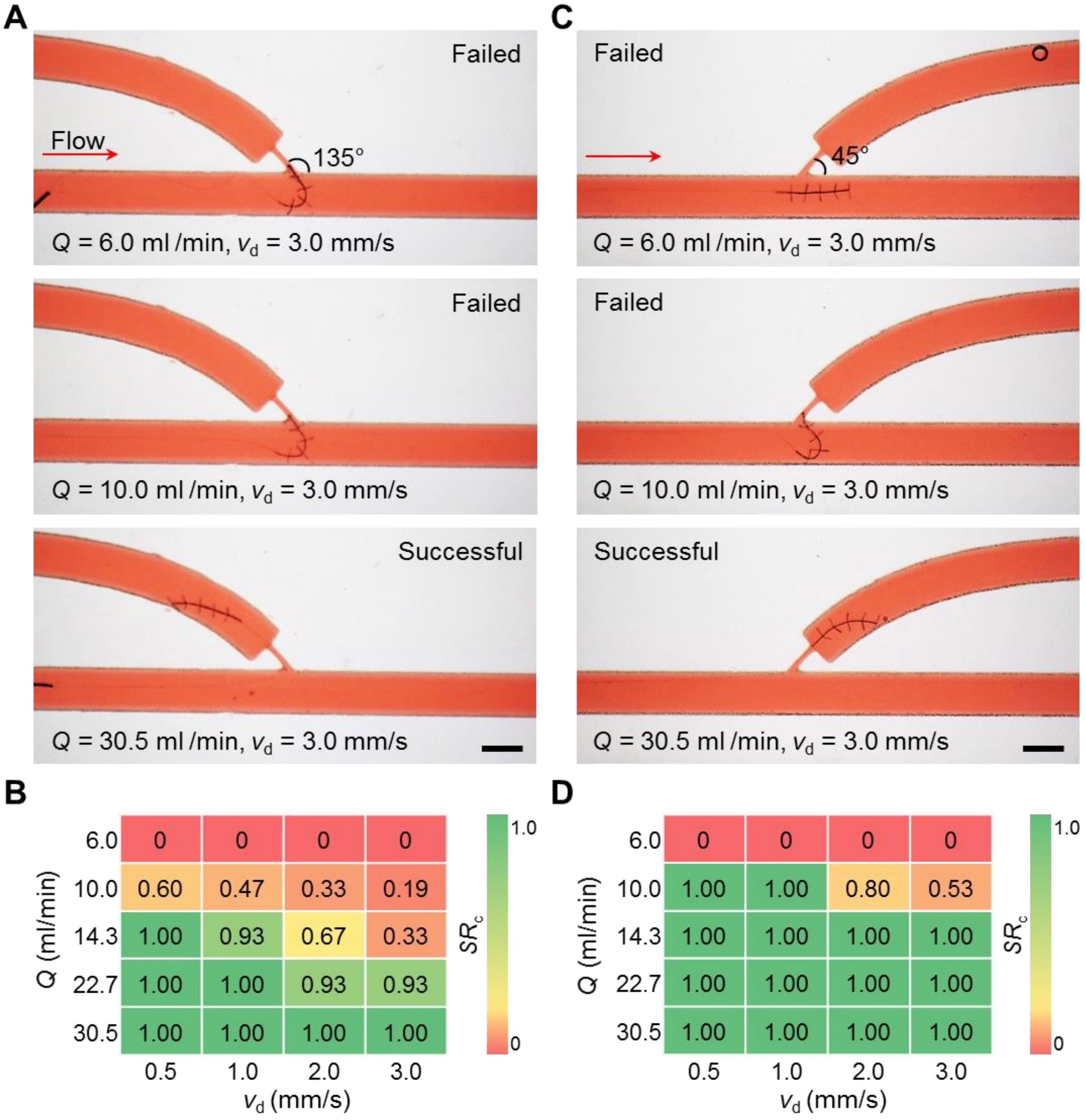

Fig. S17. Effects of flow rate Q and delivery speed v_d on success rate SR_c of crossing the side branch's MC. (**A - B**) Crossing of MC on the side branch with a bifurcating angle of 135° (phantom M). (A) Snapshots of failed and successful cases. (B) Effects of Q and v_d. (**C - D**) Crossing of MC on the side branch with a bifurcating angle of 45° (phantom N). (C) Snapshots of failed and successful cases. (D) Effects of Q and v_d. Higher flow rate Q and slower delivery speed v_d were favorable to cross the MC on the side branch. Number of trials N = 9. Scale bar: 3 mm.

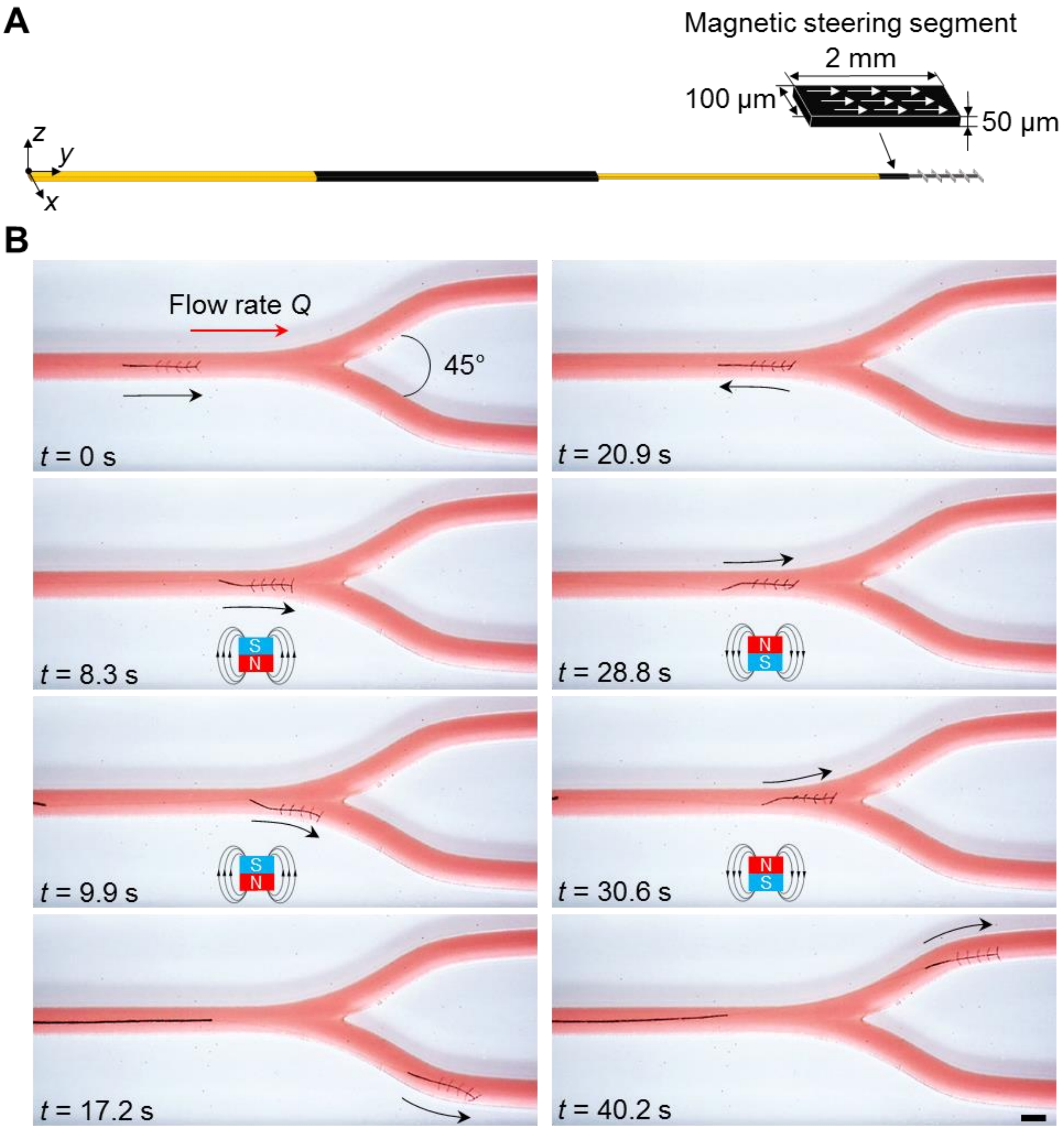

Fig. S18. Steering of the tool. (**A**) Illustration of the tool with a magnetic steering segment. The material of the steering segment was the same as the ADS. (**B**) Snapshots of steering and accessing different branches in phantom AA. The distance between the magnet and the lumen l_z was 40 mm. The pulsatile flow rate Q into the inlet was set to around 20.0 ml/min. Scale bar: 3 mm.

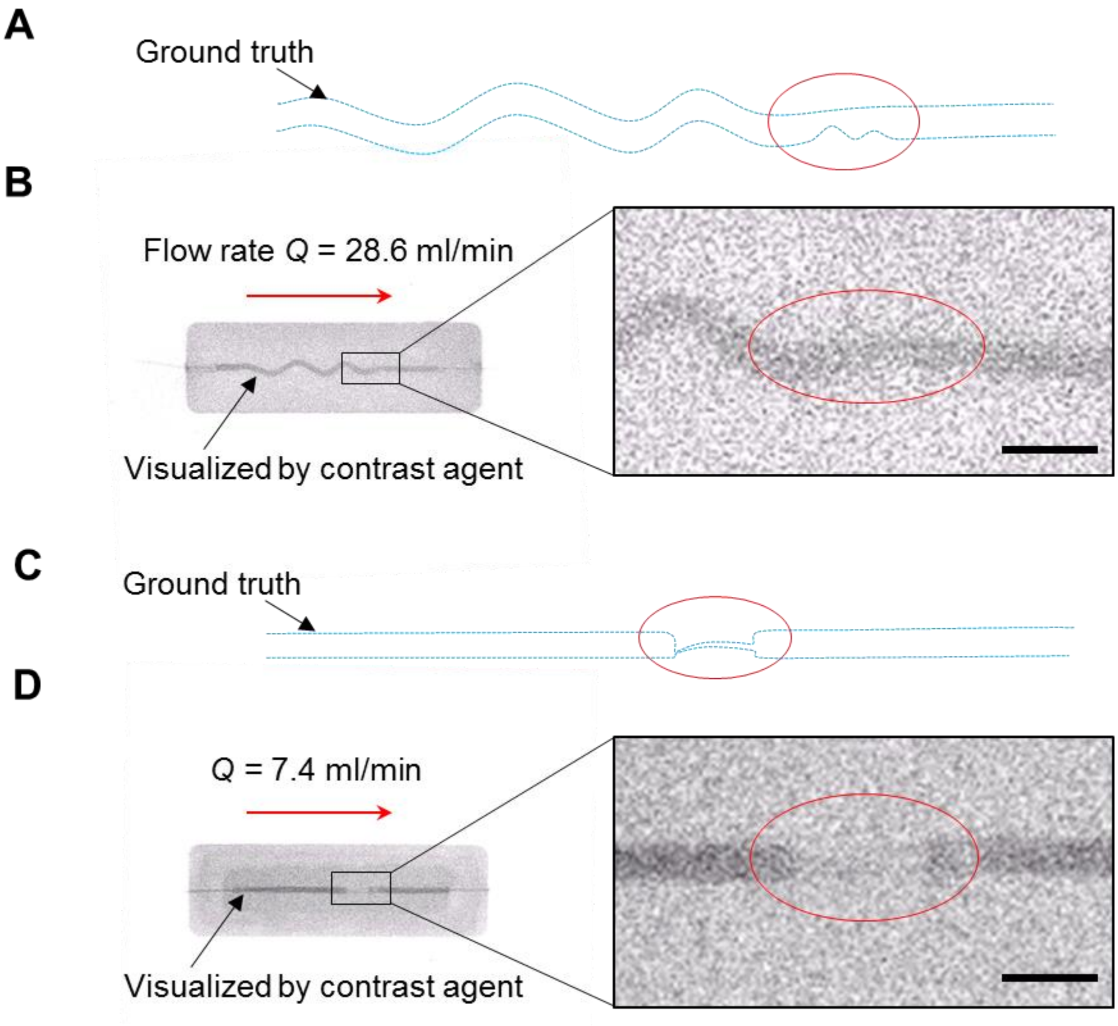

Fig. S19. Morphology visualization using medical contrast agent in agarose-based phantoms. (**A - B**) Visualization of the lumen with a partial occlusion (phantom P). (A) Ground truth of the lumen. (B) Lumen visualized by the medical contrast agent (Iomeron 400, solution for injection, Bracco UK Limited). The contrast agent could image the route of the lumen, but it was difficult to detect the detailed morphology of the partial occlusion. (**C - D**) Visualization of the lumen with a severe occlusion (phantom Q). (C) Ground truth of the lumen. (D) Lumen visualized by the medical contrast agent. The contrast agent successfully diffused to the distal end of the lumen. However, the diffusion route in the MC of the severe occlusion could not be visualized. X-ray imaging parameters for all detections were set 50 kV, 65 μA. All scale bars: 1 cm.

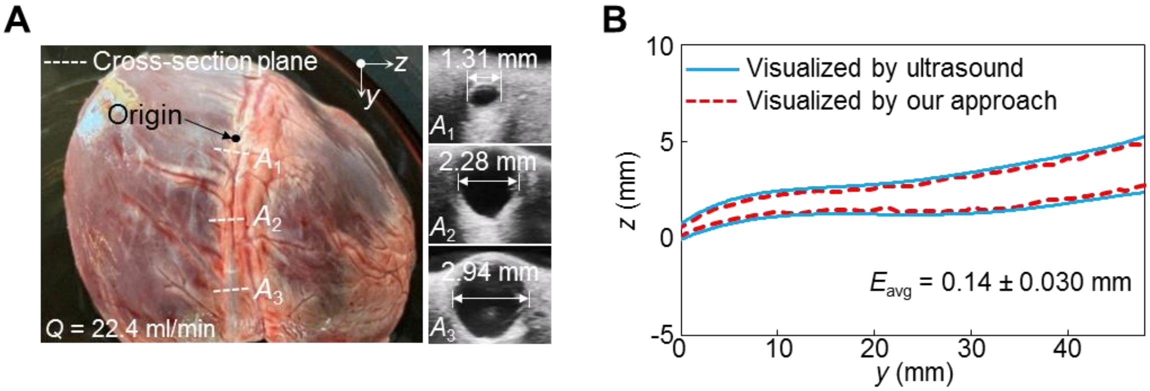

Fig. S20. Quantification of the vessel wall in a freshly cut porcine heart. (**A**) Quantification by ultrasound imaging (Vevo 3100, FUJIFILM Sonosite, Inc.) using a linear array transducer (MX 550D, FUJIFILM Visualsonics, Inc.). (**B**) Comparison of the morphology quantified by ultrasound and our approach.

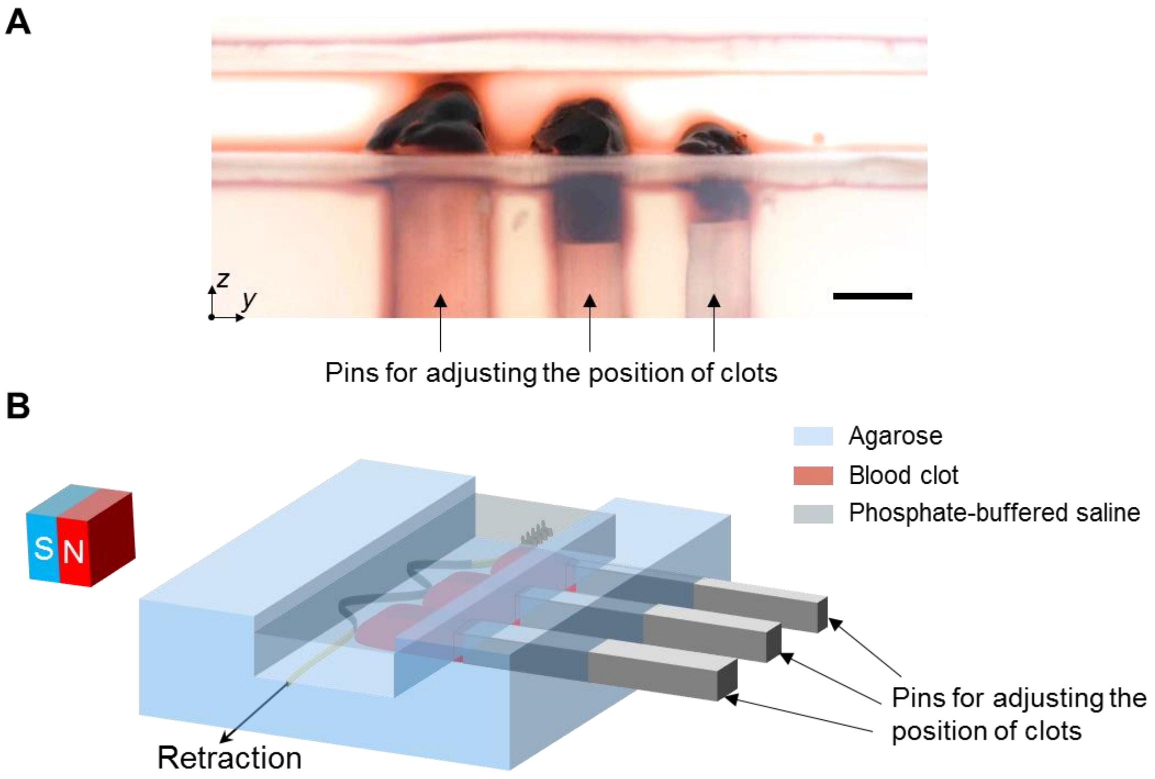

Fig. S21. Morphology visualization of blood clots fixed in an agarose-based phantom. (**A**) Snapshots of the phantom R. The positions of the blood clots were adjusted by pins. (**B**) Schematics of the visualization process. Scale bar: 3 mm.

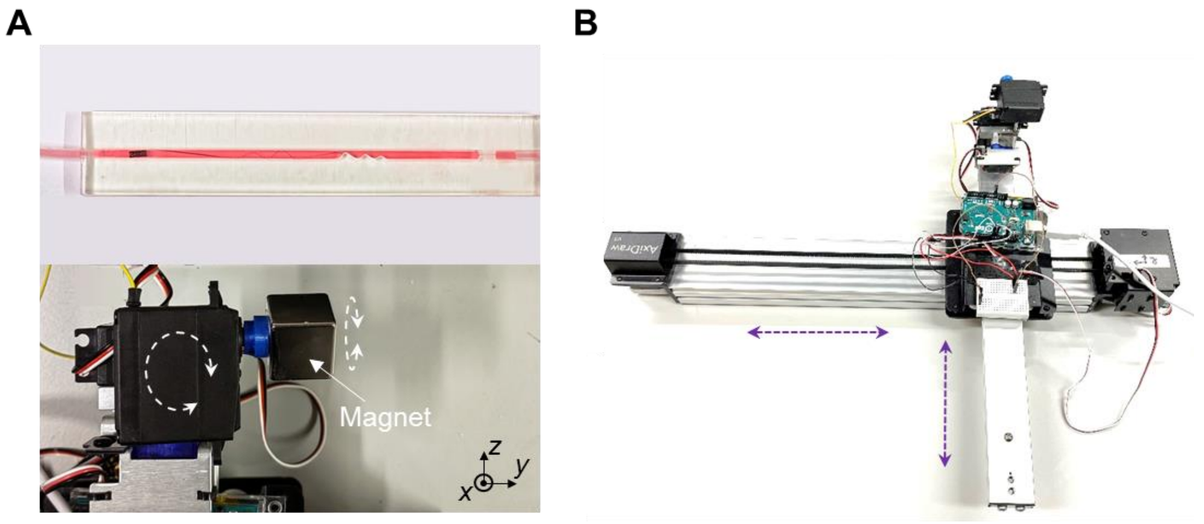

Fig. S22. Experimental setup for the tool working in untethered mode. (**A**) Servo motors used for the orientation and continuous rotation (in the *xz* and *yz* plane) of the permanent magnet. (**B**) The X-Y stage for controlling the translation of the permanent magnet integrated into the stepper motor.

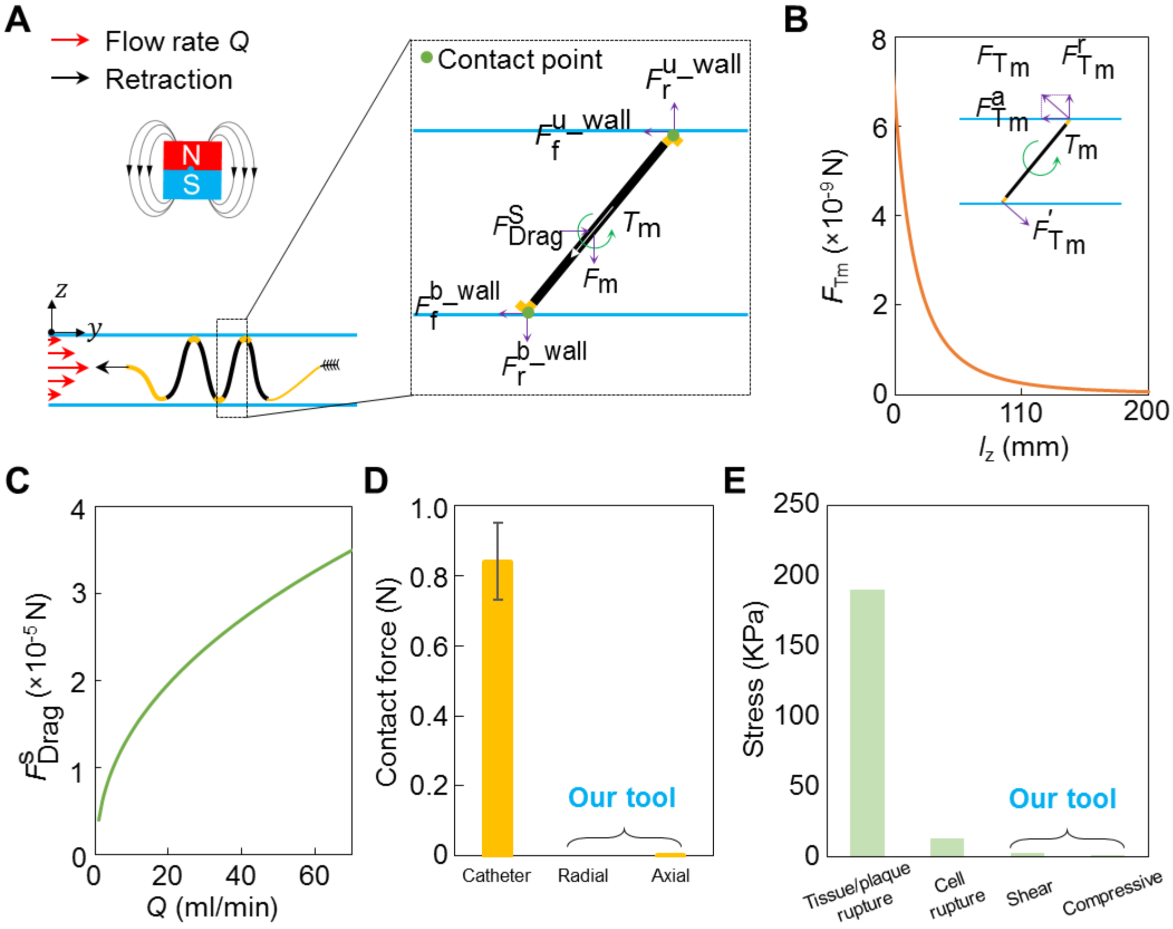

Fig. S23. Analysis of the force and contact stress of the tool. (**A**) Schematics and force diagrams when the ADS is in contact with the luminal wall. (**B**) Effects of the distance between the magnet and the lumen on the reaction force F_{T_m} generated by the magnetic torque. (**C**) Effects of flow rate Q on the drag force applied F_{Drag}^{S} on the one magnetization segment of ADS. (**D**) Comparison of the contact force generated by the medical catheter and our tool. (**E**) Comparison of stress that tissue/plaque on atherosclerotic blood vessels can withstand and the stress generated by our tool.

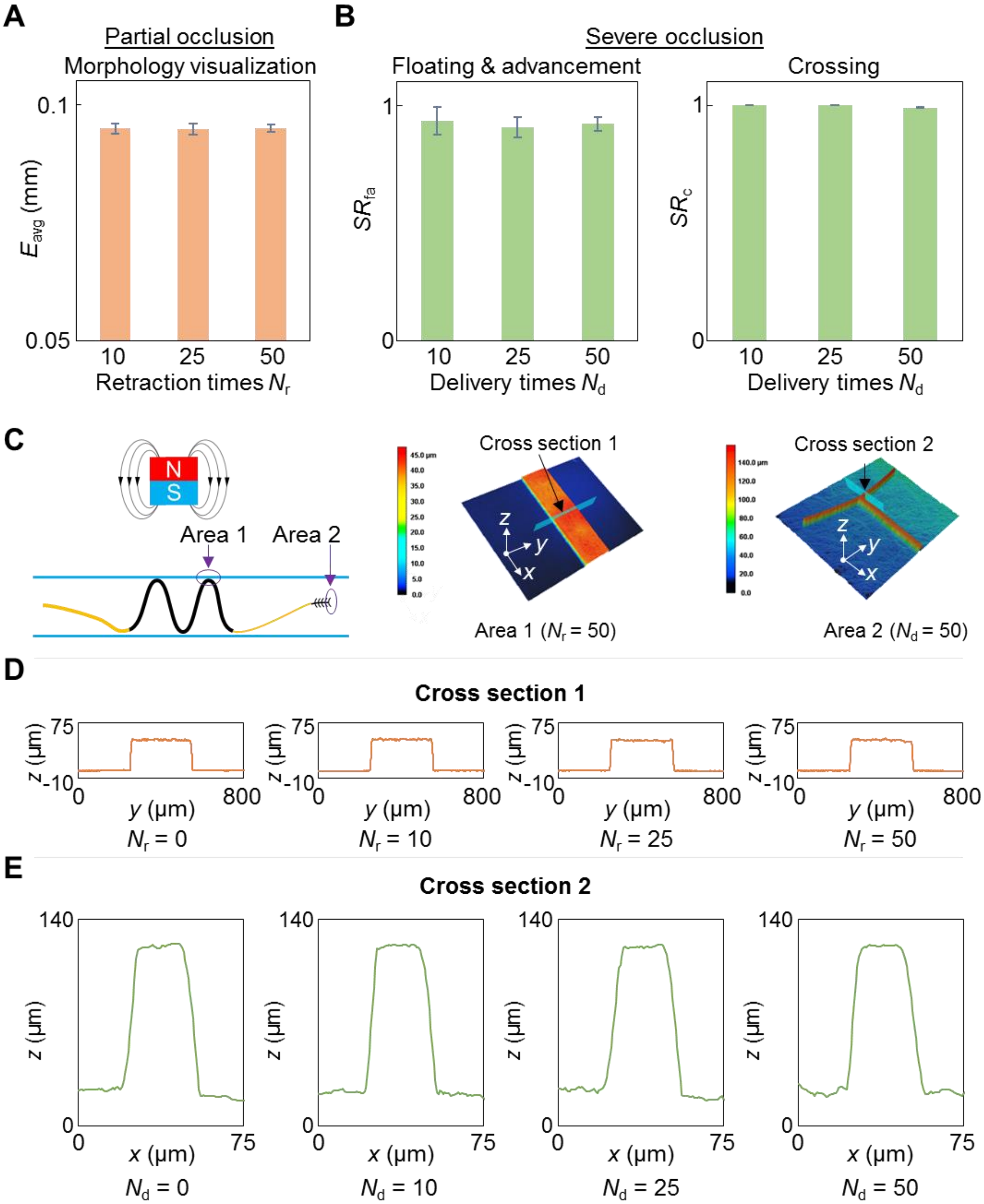

Fig. S24. Confirmation of mechanical durability. (**A**) Evaluations of ADS's function after different numbers of tests in phantom A. (**B**) Evaluations of FDS's function after different numbers of tests in phantom I. The experiments showed that the performance of the tool did not change significantly after 50 times of use (P = 0.325 in (A), 0.984, and 0.452 in (B), one-way ANOVA

test). (**C**) Surface profile of area 1 in ADS and area 2 in FDS after 50 tests. (**D**) Profile of cross section 1 after different numbers of tests. (**E**) Profile of cross section 2 after different numbers of tests. The images and height profiles were acquired by a 3D laser scanning microscope (VK-X250, Keyence Corporation). Q = 10.0 ml/min in (A) and 3.6 - 5.0 ml/min in (B), v_r = 1.0 mm/s, and l_z = 50 mm in (A), v_d = 0.5 mm/s in (B), l_b = 0.6 mm in all experiments. Error bars represent SD (N = 5). All scale bars: 0.5 mm.

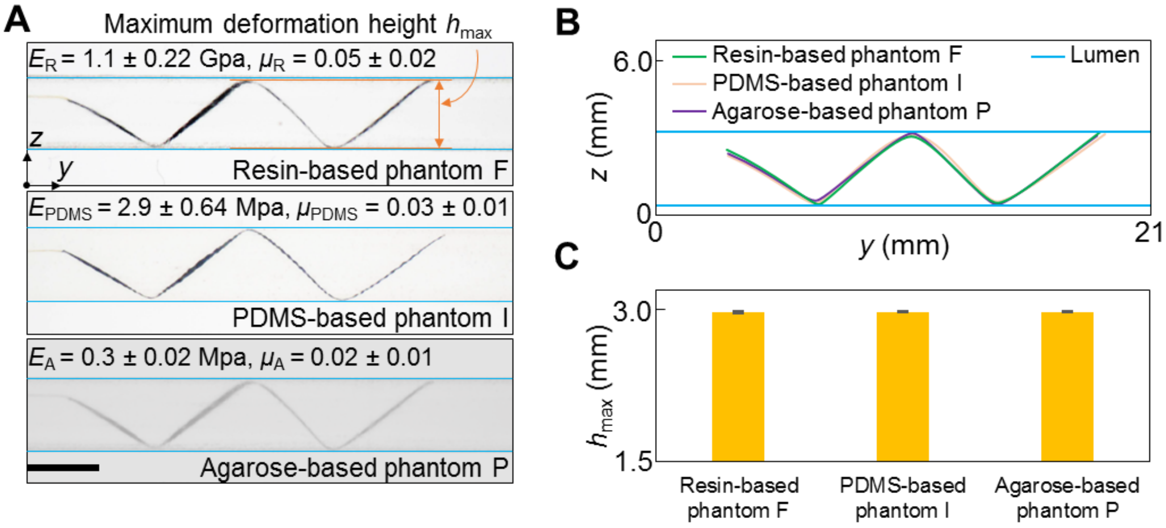

Fig. S25. Deformation of ADS in phantoms fabricated with various materials. (**A**) Snapshots of deformed ADS in normal-sized lumens of different phantoms. (**B**) Profiles of deformed ADS in different phantoms. (**C**) Maximum deformation height h_{max} of deformed ADS in different phantoms. The experiments showed that h_{max} did not change significantly in phantom fabricated with different materials (P = 0.877, one-way ANOVA test). Q = 10.0 ml/min, v_r = 1.0 mm/s, l_z = 40 mm, and l_b = 0.6 mm for all experiments. μ was the friction coefficient between the tool and the phantom. Error bars represent SD (N = 5). Scale bar: 3 mm.

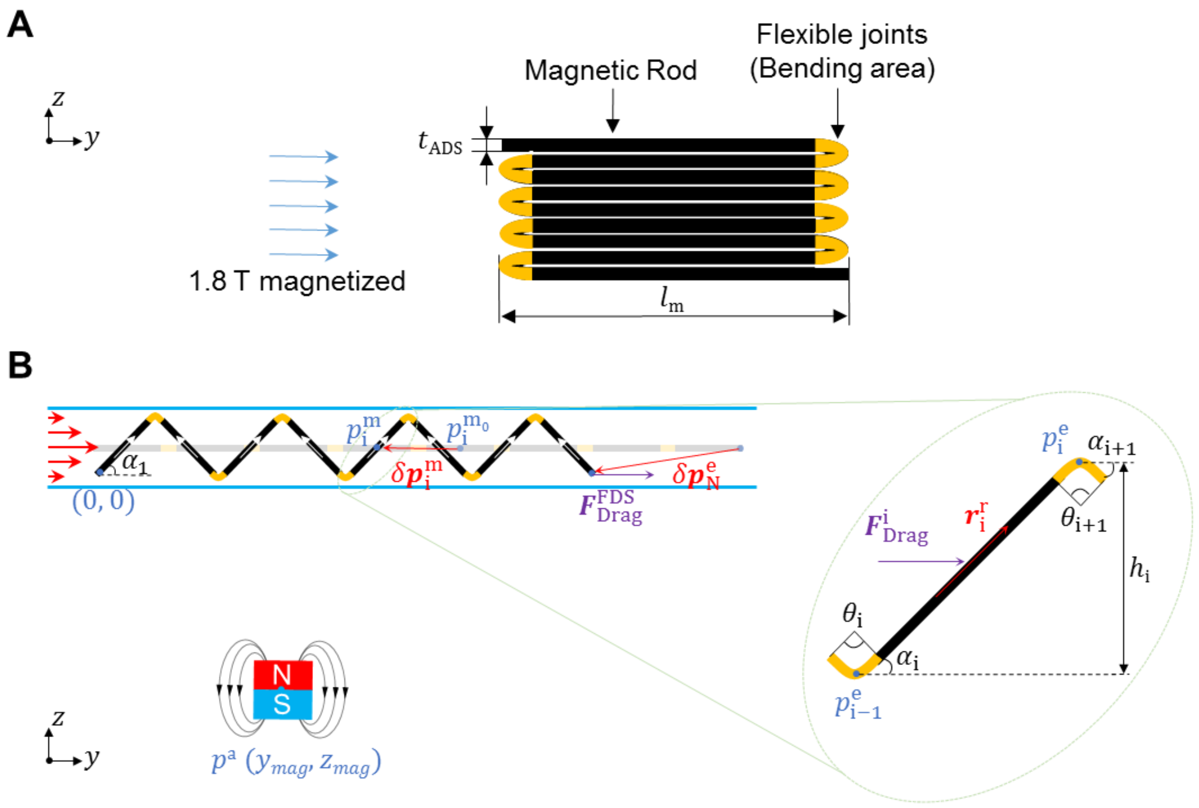

Fig. S26. Schematics of the deformation of the active deformation segment (ADS). (**A**) Illustration of the magnetization of the ADS. (**B**) Schematics of the deformed ADS.

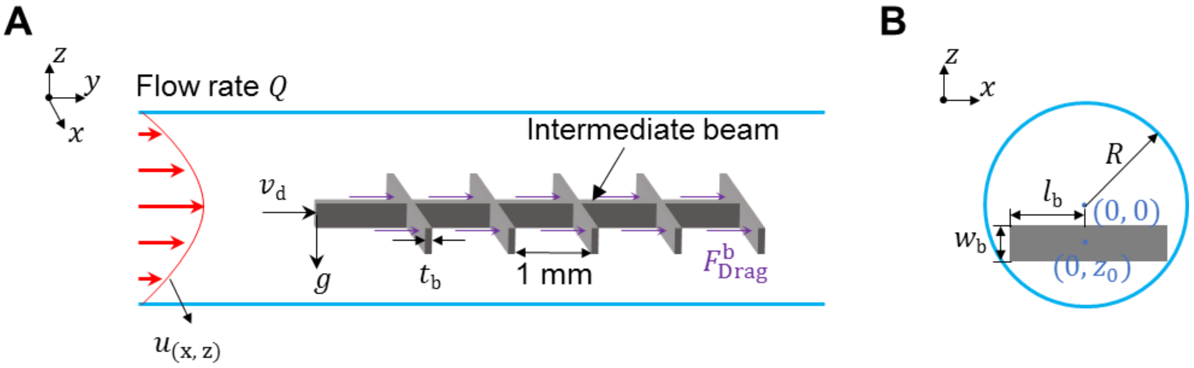

Fig. S27. Schematics of the flow drag-driven segment (FDS) in the lumen. (**A**) Diagrams of the drag force. (**B**) Location of the FDS in the *xz* plane.

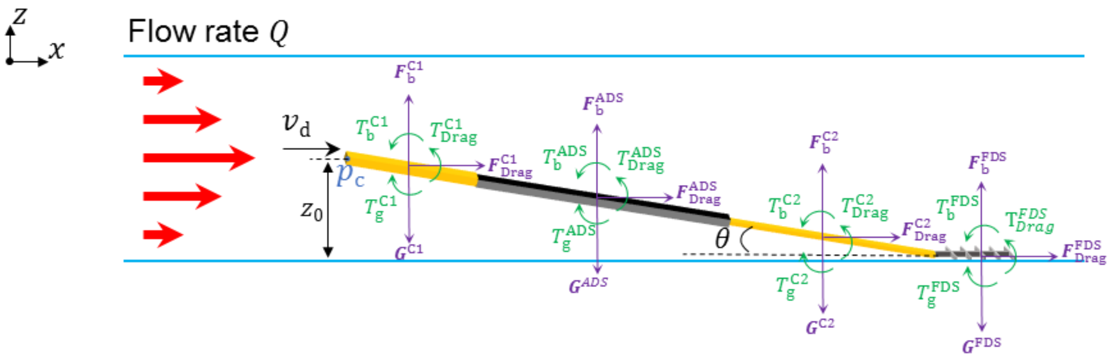

Fig. S28. Schematics of tool's floating.

Imaging modalities	Contrast agent-dependent imaging			Wire-based intravascular imaging		Our approach
	X-ray CT*	MRI*	USG*	IVUS*	OCT*	
**Spatial resolution (mm)**	0.05 - 0.20 (*120*)	0.03 - 0.10 (*120*)	0.05 - 0.50 (*120*)	0.10 – 0.20 (*121*)	0.01–0.02 (*121*)	~ 10^{-2}
**Accessible vessel diameter† (mm)**	Down to micrometers (*122*)			> 0.87 (*123*)	> 0.48 (*124*)	> 0.20
**Imaging objective**	Blood flow (*125*)			Luminal wall (*125*)		Morphological details in lumen
**Advantages**	• Large range of mapping (*122*)<br>• Large penetration depth (*122*)<br>• Noninvasive procedure (*126*)<br>• Assessment of properties of blood flow (*126*)			• Minimally invasive (*126*)<br>• Direct assessment of luminal walls (*17, 122, 125*)<br>• Analyze composition of surrounding materials, e.g., plaques (*27, 122, 125, 127*)<br>• High resolution (*121, 123, 125*)<br>• Real-time guidance (*125, 127*)		• Minimally invasive<br>• Direct and real-time depiction of morphological details<br>• Eliminate the need for contrast agent (and blood removal)<br>• Suitable for complex and tortuous vascular structure<br>• Tethered and untethered modes<br>• Soft and compliant interactions with surrounding structures
**Disadvantages**	• Indirect imaging and rely on the contrast agent to visualize the vessels (*125, 126, 128*)<br>• Contrast agents are hard to diffuse evenly and continuously along the lesions axially and radially (*129*)<br>• Struggle to accurately depict the morphological details of luminal walls (*16, 18*)<br>• High precision at the expense of safety (*130*)<br>• Potential allergy (*131*)			• Low range of mapping (*22-24*)<br>• Hard to navigate in complex and tortuous structures with relatively stiff cables and components and impose risks of perforations (*25, 27, 28*)<br>• Need to manage the blood flow (e.g., blood removal) (*3, 122*)		Currently<br>• Driven by flows<br>• Low range of mapping<br>• Cannot analyze the composition of surrounding materials
**Possible unfavorable outcomes**	• Misjudgment of the stenosis's location, grade, shape, and size (*16, 17*)<br>• Failure to identify the entry point/ tear of lesions (*14, 15*)<br>• Incorrect treatment planning and using the improperly sized medical stent or drug balloon (*19, 132, 133*)			• Vessel damage (*25, 27, 28*)<br>• Thrombosis or embolism (*25, 27*)<br>• Hematoma (*25*)<br>• Arterial dissection (*25, 27*)		• Require design and actuation optimizations given the low and high flow rates
**Clinical applications and prospectives**	• Identification of abnormalities (*16, 17*)<br>• Assessment of perfusion (*25*)<br>• Characterization of tumor (*134*)			• Assessment of plaque rupture (*27, 122, 125*)<br>• Real-time guidance for vascular interventions (*125*)<br>• Assessment of stent size and position (*27*)		• Visualize the morphological details, i.e., location, grade, shape, and size, of partial occlusions to help decide the following stenting and ballooning<br>• Locate the entry point and cross the severe occlusion

*X-ray CT: X-ray computed tomography; MRI: magnetic resonance imaging; USG: ultrasonography; IVUS: intravascular ultrasound; OCT: optical coherence tomography

Table S1. Comparison of imaging methods for visualizing morphological details in vasculature.

l_{ADS}, w_{ADS} and t_{ADS}	Length, width and thickness of the active deformation segment (ADS)
l_{FDS}, w_{FDS} and t_{FDS}	Length, width and thickness of the Fluid drag-driven segment (FDS)
l_{b}, w_{b} and t_{b}	Length, width and thickness of beams on FDS
l_{c1}, w_{c1} and t_{c1}	Length, width and thickness of the connection segment 1
l_{c2}, w_{c2} and t_{c2}	Length, width and thickness of the connection segment 2
l_{m}	Length of the magnetization segment in ADS
l_{o}	Length of occlusion
l_{mc}	Length of the microchannel (MC) inside occlusion
l_{z}	Distance between the magnet and the lumen
$l_{\mathrm{z\_min}}$	Minimum allowed distance between the magnet and the lumen
h_{max}	Maximum deformation height of the ADS
d_{n}	Diameter of the uniform-size lumen
$d_{\mathrm{c\_min}}$	Minimum diameter of the channel inside occlusion
A_{mc}	Cross-sectional area of the MC
v_{r}	Retraction speed of the tool
v_{d}	Delivery speed of the tool
R	Size of the internal plaques or thrombi
Q	Flow rate
E_{avg}	Average visualization error
SR_{fa}	Success rate of floating and advancement
SR_{c}	Success rate of crossing the MC
F_{m}	Magnetic force
T_{m}	Magnetic torque
T_{Drag}	Torques from fluidic drag
T_{b}	Torques from buoyancy
T_{g}	Torques from gravity
T_{t}	Threshold torque
η	Viscosity of blood analog

Table S2. Nomenclature of the variables

Label of phantom	Material	Fabrication	Structural features	Inlet flow rate *Q** (ml/min)
A – E	UV curable resin (Clear V4, Formlabs Inc.)	3D printing	Partial occlusion (PO)	10.0 – 40.0**
F			Normal-sized lumen (NO)	0 – 50.0**
G			PO	~ 20.0
H			PO	~12.0
I	PDMS (SylgardTM 184, Dow Inc.)	Mold technique	Severe occlusion (SO)	3.6 – 5.0
J – L				3.6 – 6.0
M – N	UV curable resin	3D printing	SO on side branches	6.0 – 30.5**
O	PDMS	Mold technique	SO	7.3 – 7.9
P	Agarose (A9539, Sigma-Aldrich)	Hybrid method	PO	28.6
Q			SO	7.4
R	Agarose + blood clots	Mold technique	PO	0
S	UV curable resin	3D printing	PO and SO	14.0
T – X			PO	~ 10.0
Y – Z	PDMS	Mold technique	SO	4.0 – 5.2
AA	UV curable resin	3D printing	Branch vessels	~ 20.0

*For partial occlusions, the flow rate is usually in the range of 10.0 – 70.0 ml/min, while for severe occlusions, flow rate is no more than 10.0 ml/min. ** range of *Q* for investigation.

Table S3. Fabrication, materials, and the flow rate for phantoms.

Fabrication	Advantages	Disadvantages
Molding* (*54*, *55*, *59*, *135*-*139*)	• Allow a wide range of materials<br>• Suitable for submillimeter-scale structure	• Limited design flexibility (e.g., 3D structures)<br>• Inefficiency
3D printing (*52*, *55*, *139*-*143*)	• Rapid prototyping<br>• High design and fabrication flexibility<br>• Suitable for multi-scale fabrication	• Limited adjustability of mechanical properties<br>• Limited available soft materials
Machining (*144*)	• Allow a wide range of materials<br>• Suitable for structures with above-millimeter sizes	• Not suitable for soft materials<br>• Not suitable for complex structures
Assembly (*127*, *138*)	• Allow the combination of different materials and components	• Bonding/connection issues between different components/materials<br>• Difficult to ensure high quality at small sizes
Lithography (*145*-*147*)	• Suitable for 2D microscale structures<br>• High resolution	• Hard to fabricate structures with complex shapes (e.g., 3D structures)

*Sacrificeable materials for negative mold: wax (*54*), water-soluble poly vinyl alcohol (PVA) filaments (*55*), sugar (*135*), acetone-soluble acrylonitrile butadiene styrene (ABS) plastic (*58*).

Table S4. Comparison of fabrication methods for vessel-related phantoms.

Material	Advantages	Disadvantages
PDMS (*54*, *55*, *135*, *136*, *145-147*)	• Good biocompatibility<br>• Strong durability<br>• Good optical transparency<br>• Enables fabrication of microstructures and complex shapes<br>• Wide range of modulus adjustment	• Not hemocompatible
Silicone polymer (*58*, *140*)	• Elastic and flexible<br>• Strong durability	• Lower transparency compared with PDMS<br>• Hydrophobic<br>• Not hemocompatible
Gelatin (*136*)	• Medium optical transparency<br>• Good biocompatibility<br>• Biodegradable	• Limited modulus adjustment range<br>• Low durability: solubility and hygroscopicity
Agar/agarose gel (*137*)	• Medium optical transparency<br>• Good biocompatibility<br>• Hydrophilic organic materials	• Low durability
Resin (*52*, *55*, *135*, *140-142*, *148*)	• A wide range of fabrication methods<br>• High durability and corrosion resistance	• Mostly not biocompatible<br>• Limited modulus adjustment range
Polycaprolactone (PCL) (*135*, *139*)	• Good biocompatibility<br>• Biodegradable<br>• FDA-approved materials for surgical implants and drug delivery devices	• Limited range of modulus adjustment
Hydrogel (*138*)	• Good biocompatibility<br>• Similar moisture, softness and mechanical properties to tissue	• Limited fabrication methods<br>• Low strength<br>• Low durability
Thermoplastic polymer (*139, 143, 144*)	• Allows fabrication of complex structures<br>• High durability	• Limited range of modulus adjustment

Table S5. Comparison of materials for vessel-related phantoms.

**Distance from the magnet to lumen l_z (mm)**	**Magnetic field *B* (T)**
0	0.450
20	0.200
40	0.076
60	0.036
80	0.021
100	0.012
120	0.007
140	0.005
160	0.003
180	0.002

Table S6. Relation of the magnetic field with the distance to the cubic actuator magnet (50 mm).

Mov. S1. Visualization of a complicated partial occlusion.

Mov. S2. Locate the entry point and cross the microchannel with the highest flow rate.

Mov. S3. Locate the entry point and cross the true microchannel.

Mov. S4. Cross the tortuous microchannel.

Mov. S5. Cross the microchannel on the side branch (45°).

Mov. S6. Cross the microchannel on the side branch (135°).

Mov. S7. Selective entry the microchannel with a relatively straight route.

Mov. S8. Cross the microchannel in the untethered mode.

Mov. S9. Visualize the morphology in the untethered mode.

REFERENCES AND NOTES

1. M. Thiriet, M. Delfour, A. Garon, in *PanVascular Medicine* (Springer Berlin Heidelberg, 2015), chap. 32, pp. 781–868.

2. D. H. K. Van Dam-Nolen, N. C. M. van Egmond, K. Dilba, K. Nies, A. G. van der Kolk, M. I. Liem, M. E. Kooi, J. Hendrikse, P. J. Nederkoorn, P. J. Koudstaal, A. van der Lugt, D. Bos, Sex differences in plaque composition and morphology among symptomatic patients with mild-to-moderate carotid artery stenosis. *Stroke* **53**, 370–378 (2022).

3. G. S. Mintz, G. Guagliumi, Intravascular imaging in coronary artery disease. *Lancet* **390**, 793–809 (2017).

4. G. S. Mintz, Z. Ali, A. Maehara, Use of intracoronary imaging to guide optimal percutaneous coronary intervention procedures and outcomes. *Heart* **107**, 755–764 (2021).

5. R.-D. Sui, C.-G. Wang, D.-W. Han, X.-Q. Zhang, Q. Li, C.-F. Xu, P.-B. Gong, Application of computed tomography angiography for evaluating clinical morphology in intracranial aneurysms–monocentric study. *J. Int. Med. Res.* **48**, 300060519894790 (2020).

6. V. Bhatia, B. Hosur, A. Kumar, Balloon-catheter buddy technique for coiling of very small aneurysm. *J. Clin. Interv. Radiol. ISVIR* **6**, 68–70 (2021).

7. H. H. Sievers, B. Rylski, M. Czerny, A. L. M. Baier, M. Kreibich, M. Siepe, F. Beyersdorf, Aortic dissection reconsidered: Type, entry site, malperfusion classification adding clarity and enabling outcome prediction. *Interact. Cardiovasc. Thorac. Surg.* **30**, 451–457 (2020).

8. D. Neves, A. Bento, R. Fernandes, L. Patricio, J. Aguiar, Spontaneous coronary artery dissection: Still a lot to learn. *Rev. Port. Cardiol.* **36**, 59.e1–59.e5 (2017).

9. D. Patel, Intravascular ultrasound imaging for the optimization of dialysis access interventions. *J. Vasc. Access* **21**, 838–846 (2020).

10. T. Wan, H. Feng, C. Tong, D. Li, Z. Qin, Automated identification and grading of coronary artery stenoses with X-ray angiography. *Comput. Methods Programs Biomed.* **167**, 13–22 (2018).

11. M. P. Opolski, S. Achenbach, CT angiography for revascularization of CTO: Crossing the borders of diagnosis and treatment. *JACC Cardiovasc. Imaging* **8**, 846–858 (2015).

12. E. S. Brilakis, K. Mashayekhi, E. Tsuchikane, N. Abi Rafeh, K. Alaswad, M. Araya, A. Avran, L. Azzalini, A. M. Babunashvili, B. Bayani, R. Bhindi, N. Boudou, M. Boukhris, N. Ž. Božinović, L. Bryniarski, A. Bufe, C. E. Buller, M. N. Burke, H. J. Büttner, P. Cardoso, M. Carlino, E. H. Christiansen, A. Colombo, K. Croce, F. Damas de los Santos, T. de Martini, J. Dens, C. di Mario, K. Dou, M. Egred, A. M. ElGuindy, J. Escaned, S. Furkalo, A. Gagnor, A. R. Galassi, R. Garbo, J. Ge, P. K. Goel, O. Goktekin, L. Grancini, J. A. Grantham, C. Hanratty, S. Harb, S. A. Harding, J. P. S. Henriques, J. M. Hill, F. A. Jaffer, Y. Jang, R. Jussila, A. Kalnins, A. Kalyanasundaram, D. E. Kandzari, H. L. Kao, D. Karmpaliotis, H. H. Kassem, P. Knaapen, R. Kornowski, O. Krestyaninov, A. V. G. Kumar, P. Laanmets, P. Lamelas, S. W. Lee, T. Lefevre, Y. Li, S. T. Lim, S. Lo, W. Lombardi, M. McEntegart, M. Munawar, J. A. Navarro Lecaro, H. M. Ngo, W. Nicholson, G. K. Olivecrona, L. Padilla, M. Postu, A. Quadros, F. H. Quesada, V. S. Prakasa Rao, N. Reifart, M. Saghatelyan, R. Santiago, G. Sianos, E. Smith, J. C. Spratt, G. W. Stone, J. W. Strange, K. Tammam, I. Ungi, M. Vo, V. H. Vu, S. Walsh, G. S. Werner, J. R. Wollmuth, E. B. Wu, R. M. Wyman, B. Xu, M. Yamane, L. F. Ybarra, R. W. Yeh, Q. Zhang, S. Rinfret, Guiding principles for chronic total occlusion percutaneous coronary intervention. *Circulation* **140**, 420–433 (2019).

13. B. Wang, K. F. Chan, K. Yuan, Q. Wang, X. Xia, L. Yang, H. Ko, Y. X. J. Wang, J. J. Y. Sung, P. W. Y. Chiu, L. Zhang, Endoscopy-assisted magnetic navigation of biohybrid soft microrobots with rapid endoluminal delivery and imaging. *Sci. Robot.* **6**, eabd2813 (2021).

14. S. Yamamoto, TCTAP C-071 intravascular ultrasound guided switchback wiring for chronic total occlusion. *J. Am. Coll. Cardiol.* **73**, S140-S141 (2019).

15. M. Nakashima, Y. Ikari, J. Aoki, K. Tanabe, S. Tanimoto, K. Hara, Intravascular ultrasound-guided chronic total occlusion wiring technique using 6 Fr catheters via bilateral transradial approach. *Cardiovasc. Interv. Ther.* **30**, 68–71 (2015).

16. R. B. Allan, C. L. Delaney, Identification of micro-channels within chronic total occlusions using contrast-enhanced ultrasound. *J. Vasc. Surg.* **74**, 606–614.e1 (2021).

17. E. A. Sanidas, IVUS in CTO lesions: “Rolling into deep”. *JACC Case Rep.* **2**, 966-967 (2020).

18. B. K. Courtney, N. R. Munce, K. J. Anderson, A. S. Thind, G. Leung, P. E. Radau, F. S. Foster, I. A. Vitkin, R. S. Schwartz, A. J. Dick, G. A. Wright, B. H. Strauss, Innovations in imaging for chronic total occlusions: A glimpse into the future of angiography's blind-spot. *Eur. Heart J.* **29**, 583–593 (2008).

19. V. Anagnostakou, G. J. Ughi, A. S. Puri, M. J. Gounis, Optical coherence tomography for neurovascular disorders. *Neuroscience* **474**, 134–144 (2021).

20. S. Aumann, S. Donner, J. Fischer, F. Müller, Optical coherence tomography (OCT): Principle and technical realization, in *High Resolution Imaging in Microscopy and Ophthalmology: New Frontiers in Biomedical Optics* (Springer International Publishing, 2019), pp. 59–85.

21. C. Schultz, M. van der Ent, P. W. Serruys, E. Regar, Optical coherence tomography to guide treatment of chronic occlusions? *JACC Cardiovasc. Interv.* **2**, 366–367 (2009).

22. K. Sadamatsu, M. Okutsu, S. Sumitsuji, T. Kawasaki, S. Nakamura, Y. Fukumoto, K. Tsujita, S. Sonoda, Y. Kobayashi, Y. Ikari, Practical utilization of cardiac computed tomography for the success in complex coronary intervention. *Cardiovasc. Interv. Ther.* **36**, 178–189 (2021).

23. S. D. Tomasello, P. Giudice, T. Attisano, M. Boukhris, A. R. Galassi, The innovation of composite core dual coil coronary guide-wire technology: A didactic coronary chronic total occlusion revascularization case report. *J. Saudi Heart Assoc.* **26**, 222–225 (2014).

24. T. Muramatsu, *Current Trend and Techniques of Percutaneous Coronary Intervention for Chronic Total Occlusion*, (Springer Nature, 2020).

25. A. Posa, A. Tanzilli, P. Barbieri, L. Steri, F. Arbia, G. Mazza, V. Longo, R. Iezzi, Digital subtraction angiography (DSA) technical and diagnostic aspects in the study of lower limb arteries. *Radiation* **2**, 376–386 (2022).

26. A. Shaffeek, J. Ravikrishnan, M. C. Rajendran, M. H. A. Rasheed, Broken catheters: A review of surgical management. *The Arab Journal of Interventional Radiology* **2**, S33 (2018).

27. R. Xu, Q. Zhao, T. Wang, Y. Yang, J. Luo, X. Zhang, Y. Feng, Y. Ma, A. A. Dmytriw, G. Yang, S. Chen, B. Yang, L. Jiao, Optical coherence tomography in cerebrovascular disease: Open up new horizons. *Transl. Stroke Res.* **14**, 137–145 (2023).

28. T. Umemoto, A. Pacchioni, D. Nikas, B. Reimers, Recent developments of imaging modalities of carotid artery stenting. *J. Cardiovasc. Surg. (Torino)* **58**, 25–34 (2017).

29. L. V. Wang, J. Yao, A practical guide to photoacoustic tomography in the life sciences. *Nat. Methods* **13**, 627–638 (2016).

30. P. K. Upputuri, M. Pramanik, Recent advances in photoacoustic contrast agents for in vivo imaging. *Wiley Interdiscip. Rev. Nanomed. Nanobiotechnol.* **12**, e1618 (2020).

31. P. J. van den Berg, K. Daoudi, W. Steenbergen, Review of photoacoustic flow imaging: Its current state and its promises. *Photoacoustics* **3**, 89–99 (2015).

32. W. Choi, D. Oh, C. Kim, Practical photoacoustic tomography: Realistic limitations and technical solutions. *J. Appl. Phys.* **127**, 230903 (2020).

33. G. K. Karagiannidis, A. Papathanasiou, P. D. Diamantoulakis, A. Saratzis, N. Saratzis, A low complexity and cost method to diagnose arterial stenosis using lightwave wearables, in *2019 IEEE 19th International Conference on Bioinformatics and Bioengineering (BIBE)*, Athens Greece, 28 to 30 October 2019, pp. 675–680.

34. P. B. Shah, Management of coronary chronic total occlusion. *Circulation* **123**, 1780–1784 (2011).

35. B. Dave, Recanalization of chronic total occlusion lesions: A critical appraisal of current devices and techniques. *J. Clin. Diagn. Res.* **10**, OE01-OE07 (2016).

36. T. Krcmar, I. Grgic Romic, V. Tomulic, T. Jakljevic, L. Bastiancic, I. Zeljkovic, Case Report: Optical coherence tomography usage for treatment of the chronically lost stent in the left main coronary artery. *Front. Cardiovasc. Med.* **9**, 825542 (2022).

37. C. Creaney, S. J. Walsh, Antegrade chronic total occlusion strategies: A technical focus for 2020. *Interv. Cardiol.* **15**, e08 (2020).

38. N. R. Sutton, E. R. Bates, Balancing the benefits, risks, and costs of chronic total occlusion percutaneous coronary intervention. *Circ. Cardiovasc. Interv.* **12**, e007809 (2019).

39. L. F. Ybarra, S. Rinfret, E. S. Brilakis, D. Karmpaliotis, L. Azzalini, J. A. Grantham, D. E. Kandzari, K. Mashayekhi, J. C. Spratt, H. C. Wijeysundera, Z. A. Ali, C. E. Buller, M. Carlino, D. J. Cohen, D. E. Cutlip, T. de Martini, C. di Mario, A. Farb, A. V. Finn, A. R. Galassi, C. M. Gibson, C. Hanratty, J. M. Hill, F. A. Jaffer, M. W. Krucoff, W. L. Lombardi, A. Maehara, P. F. A. Magee, R. Mehran, J. W. Moses, W. J. Nicholson, Y. Onuma, G. Sianos, S. Sumitsuji, E. Tsuchikane, R. Virmani, S. J. Walsh, G. S. Werner, M. Yamane, G. W. Stone, S. Rinfret, G. W. Stone; On behalf of the Chronic Total Occlusion Academic Research Consortium, Definitions and clinical trial design principles for coronary artery chronic total occlusion therapies: CTO-ARC consensus recommendations. *Circulation* **143**, 479–500 (2021).

40. Z. M. Liu, M. Li, X. Dong, Z. Ren, W. Hu, M. Sitti, Creating three-dimensional magnetic functional microdevices via molding-integrated direct laser writing. *Nat. Commun.* **13**, 2016 (2022).

41. J. Zhang, Z. Ren, W. Hu, R. H. Soon, I. C. Yasa, Z. Liu, M. Sitti, Voxelated three-dimensional miniature magnetic soft machines via multimaterial heterogeneous assembly. *Sci. Robot.* **6**, eabf0112 (2021).

42. X. Hu, I. C. Yasa, Z. Ren, S. R. Goudu, H. Ceylan, W. Hu, M. Sitti, Magnetic soft micromachines made of linked microactuator networks. *Sci. Adv.* **7**, eabe8436 (2021).

43. Y. Yan, L. Shui, S. Liu, Z. Liu, Y. Liu, Terrain adaptability and optimum contact stiffness of Vibro-bot with arrayed soft legs. *Soft Robot.* **9**, 981–990 (2022).

44. W. Hu, G. Z. Lum, M. Mastrangeli, M. Sitti, Small-scale soft-bodied robot with multimodal locomotion. *Nature* **554**, 81–85 (2018).

45. M. Sitti, Miniature soft robots—Road to the clinic. *Nat. Rev. Mater.* **3**, 74–75 (2018).

46. M. Li, A. Pal, A. Aghakhani, A. Pena-Francesch, M. Sitti, Soft actuators for real-world applications. *Nat. Rev. Mater.* **7**, 235–249 (2022).

47. P. E. Dupont, B. J. Nelson, M. Goldfarb, B. Hannaford, A. Menciassi, M. K. O'Malley, N. Simaan, P. Valdastri, G. Z. Yang, A decade retrospective of medical robotics research from 2010 to 2020. *Sci. Robot.* **6**, eabi8017 (2021).

48. X. Dong, G. Z. Lum, W. Hu, R. Zhang, Z. Ren, P. R. Onck, M. Sitti, Bioinspired cilia arrays with programmable nonreciprocal motion and metachronal coordination. *Sci. Adv.* **6**, eabc9323 (2020).

49. Z. Ren, M. Zhang, S. Song, Z. Liu, C. Hong, T. Wang, X. Dong, W. Hu, M. Sitti, Soft-robotic ciliated epidermis for reconfigurable coordinated fluid manipulation. *Sci. Adv.* **8**, eabq2345 (2022).

50. M. Han, L. Chen, K. Aras, C. Liang, X. Chen, H. Zhao, K. Li, N. R. Faye, B. Sun, J. H. Kim, W. Bai, Q. Yang, Y. Ma, W. Lu, E. Song, J. M. Baek, Y. Lee, C. Liu, J. B. Model, G. Yang, R. Ghaffari, Y. Huang, I. R. Efimov, J. A. Rogers, Catheter-integrated soft multilayer electronic arrays for multiplexed sensing and actuation during cardiac surgery. *Nat. Biomed. Eng.* **4**, 997–1009 (2020).

51. D. H. Kim, N. Lu, R. Ghaffari, Y. S. Kim, S. P. Lee, L. Xu, J. Wu, R. H. Kim, J. Song, Z. Liu, J. Viventi, B. de Graff, B. Elolampi, M. Mansour, M. J. Slepian, S. Hwang, J. D. Moss, S. M. Won, Y. Huang, B. Litt, J. A. Rogers, Materials for multifunctional balloon catheters with capabilities in cardiac electrophysiological mapping and ablation therapy. *Nat. Mater.* **10**, 316–323 (2011).

52. Y. Wu, X. Dong, J. K. Kim, C. Wang, M. Sitti, Wireless soft millirobots for climbing three-dimensional surfaces in confined spaces. *Sci. Adv.* **8**, eabn3431 (2022).

53. C. Wang, S. Wang, H. Pan, L. Min, H. Zheng, H. Zhu, G. Liu, W. Yang, X. Chen, X. Hou, Bioinspired liquid gating membrane-based catheter with anticoagulation and positionally drug release properties. *Sci. Adv.* **6**, eabb4700 (2020).

54. T. Wang, H. Ugurlu, Y. Yan, M. Li, M. Li, A. M. Wild, E. Yildiz, M. Schneider, D. Sheehan, W. Hu, M. Sitti, Adaptive wireless millirobotic locomotion into distal vasculature. *Nat. Commun.* **13**, 4465 (2022).

55. L. Pancaldi, P. Dirix, A. Fanelli, A. M. Lima, N. Stergiopulos, P. J. Mosimann, D. Ghezzi, M. S. Sakar, Flow driven robotic navigation of microengineered endovascular probes. *Nat. Commun.* **11**, 6356 (2020).

56. Y. Kim, G. A. Parada, S. Liu, X. Zhao, Ferromagnetic soft continuum. *Sci. Robot.* **4**, eaax7329 (2019).

57. Y. Kim, E. Genevriere, P. Harker, J. Choe, M. Balicki, R. W. Regenhardt, J. E. Vranic, A. A. Dmytriw, A. B. Patel, X. Zhao, Telerobotic neurovascular interventions with magnetic manipulation. *Sci. Robot.* **7**, eabg9907 (2022).

58. T. Gopesh, J. H. Wen, D. Santiago-Dieppa, B. Yan, J. S. Pannell, A. Khalessi, A. Norbash, J. Friend, Soft robotic steerable microcatheter for the endovascular treatment of cerebral disorders. *Sci. Robot.* **6**, eabf0601 (2021).

59. Z. Ren, R. Zhang, R. H. Soon, Z. Liu, W. Hu, P. R. Onck, M. Sitti, Soft-bodied adaptive multimodal locomotion strategies in fluid-filled confined spaces. *Sci. Adv.* **7**, eabh2022 (2021).

60. E. Melotti, M. Belmonte, C. Gigante, V. Mallia, S. Mushtaq, E. Conte, D. Neglia, G. Pontone, C. Collet, J. Sonck, L. Grancini, A. L. Bartorelli, D. Andreini, The role of multimodality imaging for percutaneous coronary intervention in patients with chronic total occlusions. *Front. Cardiovasc. Med.* **9**, 823091 (2022).

61. A. J. Festas, A. Ramos, J. P. Davim, Medical devices biomaterials– Areview. *Proc. Inst. Mech. Eng. L* **234**, 218–228 (2020).

62. K. Wei, M. Ragosta, J. Thorpe, M. Coggins, S. Moos, S. Kaul, Noninvasive quantification of coronary blood flow reserve in humans using myocardial contrast echocardiography. *Circulation* **103**, 2560–2565 (2001).

63. L. M. A. Crane, G. Themelis, H. J. G Arts, K. T. Buddingh, A. H. Brouwers, V. Ntziachristos, G. M. van Dam, A. G. J. van der Zee, Intraoperative near-infrared fluorescence imaging for sentinel lymph node detection in vulvar cancer: First clinical results. *Gynecol. Oncol.* **120**, 291–295 (2011).

64. J. Xue, H. Deng, X. Jia, Y. Wang, X. Lu, X. Ding, Q. Li, A. Yang, Establishing a new formula for estimating renal depth in a Chinese adult population. *Medicine (Baltimore)* **96**, e5940 (2017).

65. P. S. Rahko, Evaluation of the skin-to-heart distance in the standing adult by two-dimensional echocardiography. *J. Am. Soc. Echocardiogr.* **21**, 761–764 (2008).

66. B. H. Strauss, A. Segev, G. A. Wright, B. Qiang, N. Munce, K. J. T. Anderson, G. Leung, A. J. Dick, R. Virmani, J. Butany, Microvessels in chronic total occlusions: Pathways for successful guidewire crossing? *J. Interv. Cardiol.* **18**, 425–436 (2005).

67. N. Viceconte, R. Teijeiro-Mestre, N. Foin, A. C. Lindsay, C. D. Mario, Optical coherence tomography to guide the treatment of chronic total occlusions, in *Chronic Total Occlusions: A Guide to Recanalization* (John Wiley & Sons, 2013), pp. 60–66.

68. P. Fefer, M. Carlino, B. H. Strauss, Intraplaque therapies for facilitating percutaneous recanalization of chronic total occlusions. *Can. J. Cardiol.* **26**, 32A–36A (2010).

69. D. J. Sobczynski, M. B. Fish, C. A. Fromen, M. Carasco-Teja, R. M. Coleman, O. Eniola-Adefeso, Drug carrier interaction with blood: A critical aspect for high-efficient vascular-targeted drug delivery systems. *Ther. Deliv.* **6**, 915–934 (2015).

70. M. S. Ahmad, N. Suardi, A. Shukri, H. Mohammad, A. A. Oglat, A. Alarab, O. Makhamrah, Chemical characteristics, motivation and strategies in choice of materials used as liver phantom: A literature review. *J. Med. Ultrasound* **28**, 7–16 (2020).

71. A. Tejo-Otero, F. Fenollosa-Artés, I. Achaerandio, S. Rey-Vinolas, I. Buj-Corral, M. Á. Mateos-Timoneda, E. Engel, Soft-tissue-mimicking using hydrogels for the development of phantoms. *Gels* **8**, 40 (2022).

72. Y. Yu, H. Yuk, G. A. Parada, Y. Wu, X. Liu, C. S. Nabzdyk, K. Youcef-Toumi, J. Zang, X. Zhao, Multifunctional "hydrogel skins" on diverse polymers with arbitrary shapes. *Adv. Mater.* **31**, e1807101 (2019).

73. A. Volk, C. J. Kahler, Density model for aqueous glycerol solutions. *Exp. Fluids* **59**, 75 (2018).

74. M. Y. Yousif, D. W. Holdsworth, T. L. Poepping, A blood-mimicking fluid for particle image velocimetry with silicone vascular models. *Exp. Fluids* **50**, 769–774 (2011).

75. D. J. Lin, N. D. Jiao, Z. D. Wang, L. Q. Liu, A magnetic continuum robot with multi-mode control using opposite-magnetized magnets. *IEEE Robot. Autom. Lett.* **6**, 2485–2492 (2021).

76. B. Xu, C. Li, Y. Guo, K. Xu, Y. Yang, J. Yu, Current understanding of chronic total occlusion of the internal carotid artery. *Biomed. Rep.* **8**, 117–125 (2018).

77. O. Kawarada, Y. Yokoi, Y. Honda, P. J. Fitzgerald, Awareness of anatomical variations for infrapopliteral intervention. *Catheter. Cardiovasc. Interv.* **76**, 888–894 (2010).

78. S. Banerjee, K. Sarode, A. Patel, A. Mohammad, R. Parikh, E. J. Armstrong, S. Tsai, N. W. Shammas, E. S. Brilakis, Comparative assessment of guidewire and microcatheter vs a crossing device–based strategy to traverse infrainguinal peripheral artery chronic total occlusions. *J. Endovasc. Ther.* **22**, 525–534 (2015).

79. Y.-Y. Zhang, Endovascular recanalization for chronic total occlusion of intracranial vertebral artery: A mini review. *J. Neuroendovascular Ther.* **14**, 81–84 (2020).

80. F. Gao, H. Zheng, X. Guo, X. Sun, Z. Miao, A novel angiographic classification for the endovascular recanalization of symptomatic nonacute extracranial vertebral artery occlusion. *J. Neurointerv. Surg.* **14**, 210–214 (2022).

81. V. N. Krishnamurthy, J. L. Eliason, P. K. Henke, J. E. Rectenwald, Intravascular ultrasound–Guided true lumen reentry device for recanalization of unilateral chronic total occlusion of iliac arteries: Technique and follow-up. *Ann. Vasc. Surg.* **24**, 487–497 (2010).

82. C. J. Grilli, C. R. Fedele, O. M. Tahir, C. W. Wrigley, M. J. Garcia, G. Kimbiris, D. J. Agriantonis, D. A. Leung, Recanalization of chronic total occlusions of the superior mesenteric artery in patients with chronic mesenteric ischemia: Technical and clinical outcomes. *J. Vasc. Interv. Radiol.* **25**, 1515–1522 (2014).

83. F. F. Zhou, Y. H. Liu, P. C. Ge, Z. H. Chen, X. Q. Ding, J. Y. Liu, Q. W. Jia, F. H. An, L. H. Li, L. S. Wang, W. Z. Ma, Z. J. Yang, E. Z. Jia, Coronary artery diameter is inversely associated with the severity of coronary lesions in patients undergoing coronary angiography. *Cell. Physiol. Biochem.* **43**, 1247–1257 (2017).

84. H. V. Anderson, M. J. Stokes, M. Leon, S. A. Abu-Halawa, Y. Stuart, R. L. Kirkeeide, Coronary artery flow velocity is related to lumen area and regional left ventricular mass. *Circulation* **102**, 48–54 (2000).

85. J. H. Moon, D. Y. Lee, W. C. Cha, M. J. Chung, K. S. Lee, B. H. Cho, J. H. Choi, Automatic stenosis recognition from coronary angiography using convolutional neural networks. *Comput. Methods Programs Biomed.* **198**, 105819 (2021).

86. M. Hokari, S. Kuroda, H. Yasuda, N. Nakayama, S. Abe, Y. Iwasaki, H. Saito, Lumen morphology in mild-to-moderate internal carotid artery stenosis correlates with neurological symptoms. *J. Neuroimaging* **21**, 348–354 (2011).

87. A. M. Elhfnawy, P. U. Heuschmann, M. Pham, J. Volkmann, F. Fluri, Stenosis length and degree interact with the risk of cerebrovascular events related to internal carotid artery stenosis. *Front. Neurol.* **10**, 317 (2019).

88. B. N. Modi, S. Sankaran, H. J. Kim, H. Ellis, C. Rogers, C. A. Taylor, R. Rajani, D. Perera, Predicting the physiological effect of revascularization in serially diseased coronary arteries. *Circ. Cardiovasc. Interv.* **12**, e007577 (2019).

89. M. Carlino, G. L. Buchanan, C. Godino, The microchannel technique, in *Chronic Total Occlusions: A Guide to Recanalization*, R. Waksman, S. Saito, Eds. (John Wiley & Sons, 2013), pp. 166–171.

90. A. S. Thind, G. Leung, N. R. Munce, J. J. Graham, K. J. T. Anderson, A. J. Dick, B. H. Strauss, G. A. Wright, F. S. Foster, Investigation of micro-ultrasound for microvessel imaging in a model of chronic total occlusion. *Ultrason. Imaging* **29**, 167–181 (2007).

91. K. Yokoi, S. Sonoda, G. Yoshioka, K. Jojima, M. Natsuaki, K. Node, Proximal optimization technique facilitates wire entry into stumpless chronic total occlusion of side branch. *JACC Cardiovasc. Interv.* **14**, e231–e233 (2021).

92. Y. T. Wong, Endovascular treatment of diabetic foot ischemic ulcer—Technical review. *J. Interv. Med.* **3**, 17–26 (2020).

93. S. Sumitsuji, K. Shokry, M. Mahmoud, M. A. A. Elbaset, Retrograde versus antegrade percutaneous coronary intervention of patients with chronic total occlusion of the coronary arteries by using coronary angiography. *Eur. J. Pharm. Med. Res.* **4**, 196–207 (2017).

94. A. Sakes, E. Regar, J. Dankelman, P. Breedveld, Crossing total occlusions: Navigating towards recanalization. *Cardiovasc. Eng. Technol.* **7**, 103–117 (2016).

95. N. R. Munce, G. A. Wright, A. Mariampillai, B. A. Standish, M. K. K. Leung, L. Tan, K. Lee, B. K. Courtney, A. A. Teitelbaum, B. H. Strauss, I. A. Vitkin, V. X. D. Yang, Doppler optical coherence tomography for interventional cardiovascular guidance: In vivo feasibility and forward-viewing probe flow phantom demonstration. *J. Biomed. Opt.* **15**, 011103 (2010).

96. A. Avci, S. Fidan, M. M. Tabakçı, C. Toprak, E. Alizade, E. Acar, E. Bayam, M. Tellice, A. Naser, R. Kargın, Association between the Gensini score and carotid artery stenosis. *Korean Circ. J.* **46**, 639–645 (2016).

97. Z. Szijgyarto, R. Rampat, G. S. Werner, C. Ho, N. Reifart, T. Lefevre, Y. Louvard, A. Avran, M. Kambis, H. J. Buettner, C. di Mario, A. Gershlick, J. Escaned, G. Sianos, A. Galassi, R. Garbo, O. Goktekin, M. Meyer-Gessner, B. Lauer, S. Elhadad, A. Bufe, N. Boudou, H. Sievert, V. Martin-Yuste, L. Thuesen, A. Erglis, E. Christiansen, J. Spratt, L. Bryniarski, T. Clayton, D. Hildick-Smith, Derivation and validation of a chronic total coronary occlusion intervention procedural success score from the 20,000-patient EuroCTO registry: The EuroCTO (CASTLE) score. *JACC Cardiovasc. Interv.* **12**, 335–342 (2019).

98. G. Christopoulos, D. E. Kandzari, R. W. Yeh, F. A. Jaffer, D. Karmpaliotis, M. R. Wyman, K. Alaswad, W. Lombardi, J. A. Grantham, J. Moses, G. Christakopoulos, M. N. J. Tarar, B. V. Rangan, N. Lembo, S. Garcia, D. Cipher, C. A. Thompson, S. Banerjee, E. S. Brilakis, Development and validation of a novel scoring system for predicting technical success of chronic total occlusion percutaneous coronary interventions: The PROGRESS CTO (Prospective Global Registry for the Study of Chronic Total Occlusion Intervention) score. *JACC: Cardiovasc. Interv.* **9**, 1–9 (2016).

99. D. Syrseloudis, G. G. Secco, E. A. Barrero, A. C. Lindsay, M. Ghione, K. Kilickesmez, N. Foin, R. Martos, C. D. Mario, Increase in J-CTO lesion complexity score explains the disparity between recanalisation success and evolution of chronic total occlusion strategies: Insights from a single-centre 10-year experience. *Heart* **99**, 474–479 (2013).

100. R. Behling, F. Gruner, Diagnostic X-ray sources-present and future. *Nucl. Instrum. Methods Phys. Res. A: Accel. Spectrom. Detect. Assoc. Equip.* **878**, 50–57 (2018).

101. J. T. Bushberg, J. M. Boone, *The Essential Physics of Medical Imaging* (Lippincott Williams & Wilkins, 2011).

102. M. U. Ghani, M. D. Wong, L. Ren, D. Wu, B. Zheng, J. X. Rong, X. Wu, H. Liu, Characterization of continuous and pulsed emission modes of a hybrid micro focus x-ray source for medical imaging applications. *Nucl. Instrum. Methods Phys. Res. A* **853**, 70–77 (2017).

103. K. Horner, V. Rushton, K. Tsiklakis, P. N. Hirschmann, P.F. van der Stelt, A,-M. Glenny, X. L. Velders, S. Pavitt, “European guidelines on radiation protection in dental radiology; The safe use of radiographs in dental practice” (European Commission, Directorate-General for Energy and Transport. Radiation Protection Publications Office, 2004), ISBN: 92-984-5958-1.

104. ACO Radiology, ACR-SPR practice parameter for imaging pregnant or potentially pregnant adolescents and women with ionizing radiation (Reston, VA, 2018).

105. H. J. Park, J. H. Son, T. B. Kim, M. K. Kang, K. Han, E. H. Kim, A. Y. Kim, S. H. Park, Relationship between lower dose and injection speed of iodinated contrast material for CT and acute hypersensitivity reactions: An observational study. *Radiology* **293**, 565–572 (2019).

106. S. E. Mirvis, Diagnostic imaging of the urinary system following blunt trauma. *Clin. Imaging* **13**, 269–280 (1989).

107. J. C. Hellinger, A. Pena, M. Poon, F. P. Chan, M. Epelman, Pediatric computed tomographic angiography: Imaging the cardiovascular system gently. *Radiol. Clin. North Am.* **48**, 439–467 (2010).

108. N. Munce, “The potential of optical coherence tomography for intravascular imaging of chronic total occlusions,” thesis, University of Toronto, Toronto, CA (2009).

109. Y. I. Cho, D. J. Cho, R. S. Rosenson, Endothelial shear stress and blood viscosity in peripheral arterial disease. *Curr. Atheroscler. Rep.* **16**, 404 (2014).

110. Y. A. Çengel, J. M. Cimbala, *Fluid Mechanics: Fundamentals and Applications* (McGraw-Hill Higher Education, 2010).

111. E. Janssen, Flow past a flat plate at low Reynolds numbers. *J. Fluid Mech.* **3**, 329–343 (1958).

112. S. Tomotika, T. Aoi, The steady flow of a viscous fluid past an elliptic cylinder and a flat plate at small reynolds numbers. *Q. J. Mech. Appl.Math.* **6**, 290–312 (1953).

113. K. M. In, D. H. Choi, M. U. Kim, Two-dimensional viscous flow past a flat plate. *Fluid Dyn. Res.* **15**, 13–24 (1995).

114. V. E. Donohue, F. McDonald, R. Evans, In vitro cytotoxicity testing of neodymium-iron-boron magnets. *J. Appl. Biomater.* **6**, 69–74 (1995).

115. K. P. Rajan, A. Al-Ghamdi, R. Parameswar, G. B. Nando, Blends of thermoplastic polyurethane and polydimethylsiloxane rubber: Assessment of biocompatibility and suture holding strength of membranes. *Int J Biomater* **2013**, 240631 (2013).

116. S. Kim, S. H. Ye, A. Adamo, R. A. Orizondo, J. Jo, S. K. Cho, W. R. Wagner, A biostable, anti-fouling zwitterionic polyurethane-urea based on PDMS for use in blood-contacting medical devices. *J. Mater. Chem. B* **8**, 8305–8314 (2020).

117. V. Thondapu, C. V. Bourantas, N. Foin, I.-K. Jang, P. W. Serruys, P. Barlis, Biomechanical stress in coronary atherosclerosis: Emerging insights from computational modelling. *Eur. Heart J.* **38**, 81–92 (2016).

118. A. Hooglugt, O. Klatt, S. Huveneers, Vascular stiffening and endothelial dysfunction in atherosclerosis. *Curr. Opin. Lipidol.* **33**, 353–363 (2022).

119. Y. Tang, M. Li, T. Wang, X. Dong, W. Hu, M. Sitti, Wireless miniature magnetic phase-change soft actuators. *Adv. Mater.* **34**, e2204185 (2022).

120. J. Wallyn, N. Anton, S. Akram, T. F. Vandamme, Biomedical imaging: Principles, technologies, clinical aspects, contrast agents, limitations and future trends in nanomedicines. *Pharm. Res.* **36**, 78 (2019).

121. E. Rothstein, H. Aronow, B. M. Hawkins, M. N. Young, Intravascular imaging for peripheral vascular disease and endovascular intervention. *Curr. Cardiovasc. Imaging Rep.* **13**, 551861 (2020).

122. L. E. Mantella, K. Liblik, A. M. Johri, Vascular imaging of atherosclerosis: Strengths and weaknesses. *Atherosclerosis* **319**, 42–50 (2021).

123. R. Varada, L. Nemani, S. S. Kanugagadda, Intravascular ultrasound. *Indian J. Cardiovasc. Dis. Women WINCARS* **04**, 213–227 (2019).

124. J. Li, S. Thiele, B. C. Quirk, R. W. Kirk, J. W. Verjans, E. Akers, C. A. Bursill, S. J. Nicholls, A. M. Herkommer, H. Giessen, R. A. McLaughlin, Ultrathin monolithic 3D printed optical coherence tomography endoscopy for preclinical and clinical use. *Light Sci. Appl.* **9**, 124 (2020).

125. A. Boese, A. K. Sivankutty, M. Friebe, Optical endovascular imaging combining endoscopy, NBI and OCT, a feasibility study. *Curr. Dir. Biomed. Eng.* **5**, 577–580 (2019).

126. O. Sarioglu, A. E. Capar, U. Belet, Interventional treatment options in pseudoaneurysms: Different techniques in different localizations. *Pol. J. Radiol.* **84**, E319–E327 (2019).

127. U. Bozuyuk, A. Aghakhani, Y. Alapan, M. Yunusa, P. Wrede, M. Sitti, Reduced rotational flows enable the translation of surface-rolling microrobots in confined spaces. *Nat. Commun.* **13**, 6289 (2022).

128. K. C. Koskinas, G. J. Ughi, S. Windecker, G. J. Tearney, L. Raber, Intracoronary imaging of coronary atherosclerosis: Validation for diagnosis, prognosis and treatment. *Eur. Heart J.* **37**, 524–535 (2016).

129. M. Michalska, W. Kazimierczak, W. Leszczynski, K. Nadolska, L. Bryl, Contemporary follow-up imaging after endovascular repair of lower extremity atherosclerotic lesions. *Pol. J. Radiol.* **83**, E521–E529 (2018).

130. E. Lin, A. Alessio, What are the basic concepts of temporal, contrast, and spatial resolution in cardiac CT? *J. Cardiovasc. Comput. Tomogr.* **3**, 403–408 (2009).

131. H. Rafii-Tari, C. J. Payne, G. Z. Yang, Current and emerging robot-assisted endovascular catheterization technologies: A review. *Ann. Biomed. Eng.* **42**, 697–715 (2014).

132. P. Agostoni, J. Kortsmit, A. Colombo, Accurate stent placement in challenging percutaneous coronary interventions using the stent positioning assist system. *Cardiovasc. Revasc. Med.* **43**, 123–129 (2022).

133. P. T. Campbell, K. R. Kruse, C. R. Kroll, J. Y. Patterson, M. J. Esposito, The impact of precise robotic lesion length measurement on stent length selection: Ramifications for stent savings. *Cardiovasc. Revasc. Med.* **16**, 348–350 (2015).

134. A. Y. Park, B. K. Seo, Up-to-date Doppler techniques for breast tumor vascularity: Superb microvascular imaging and contrast-enhanced ultrasound. *Ultrasonography* **37**, 98–106 (2018).

135. L. M. Bellan, S. P. Singh, P. W. Henderson, T. J. Porri, H. G. Craighead, J. A. Spector, Fabrication of an artificial 3-dimensional vascular network using sacrificial sugar structures. *Soft Matter* **5**, 1354–1357 (2009).

136. L. K. Ryan, F. S. Foster, Tissue equivalent vessel phantoms for intravascular ultrasound. *Ultrasound Med. Biol.* **23**, 261–273 (1997).

137. B. Rivkin, C. Becker, B. Singh, A. Aziz, F. Akbar, A. Egunov, D. D. Karnaushenko, R. Naumann, R. Schäfer, M. Medina-Sánchez, D. Karnaushenko, O. G. Schmidt, Electronically integrated microcatheters based on self-assembling polymer films. *Sci. Adv.* **7**, eabl5408 (2021).

138. H. Nisar, J. Moore, R. Piazza, E. Maneas, E. C. S. Chen, T. M. Peters, A simple, realistic walled phantom for intravascular and intracardiac applications. *Int. J. Comput. Assist. Radiol. Surg.* **15**, 1513–1523 (2020).

139. J. Cantrell, S. Rohde, D. Damiani, R. Gurnani, L. DiSandro, J. Anton, A. Young, A. Jerez, D. Steinbach, C. Kroese, P. Ifju, Experimental characterization of the mechanical properties of 3D printed ABS and polycarbonate parts. *Adv. Opt. Methods Exp. Mech.* **3**, 89–105 (2017).

140. D. Jin, Q. Wang, K. F. Chan, N. Xia, H. Yang, Q. Wang, S. C. H. Yu, L. Zhang, Swarming self-adhesive microgels enabled aneurysm on-demand embolization in physiological blood flow. *Sci. Adv.* **9**, eadf9278 (2023).

141. S. Jeon, A. K. Hoshiar, K. Kim, S. Lee, E. Kim, S. Lee, J. Y. Kim, B. J. Nelson, H. J. Cha, B. J. Yi, H. Choi, A magnetically controlled soft microrobot steering a guidewire in a three-dimensional phantom vascular network. *Soft Robot* **6**, 54–68 (2019).

142. G. Pittiglio, P. Lloyd, T. da Veiga, O. Onaizah, C. Pompili, J. H. Chandler, P. Valdastri, Patient-specific magnetic catheters for atraumatic autonomous endoscopy. *Soft Robot.* **9**, 1120–1133 (2022).

143. J. Hwang, S. Jeon, B. Kim, J. Y. Kim, C. Jin, A. Yeon, B. J. Yi, C. H. Yoon, H. J. Park, S. Pané, B. J. Nelson, H. Choi, An electromagnetically controllable microrobotic interventional system for targeted, real-time cardiovascular intervention. *Adv. Healthc. Mater.* **11**, 2102529 (2022).

144. Y. Zhang, X. Wu, R. A. Vadlamani, Y. Lim, J. Kim, K. David, E. Gilbert, Y. Li, R. Wang, S. Jiang, A. Wang, H. Sontheimer, D. English, S. Emori, R. V. Davalos, S. Poelzing, X. Jia, Multifunctional ferromagnetic fiber robots for navigation, sensing, and treatment in minimally invasive surgery. bioRxiv 2023.01.27.525973 (2023). https://doi.org/10.1101/2023.01.27.525973.

145. R. Lima, S. Wada, S. Tanaka, M. Takeda, T. Ishikawa, K. I. Tsubota, Y. Imai, T. Yamaguchi, In vitro blood flow in a rectangular PDMS microchannel: Experimental observations using a confocal micro-PIV system. *Biomed. Microdevices* **10**, 153–167 (2008).

146. J. S. Choi, Y. Piao, T. S. Seo, Fabrication of a circular PDMS microchannel for constructing a three-dimensional endothelial cell layer. *Bioprocess Biosyst. Eng.* **36**, 1871–1878 (2013).

147. M. Waqas, M. Mokin, J. Lim, K. Vakharia, M. E. Springer, K. M. Meess, R. W. Ducharme, C. N. Ionita, S. V. S. Nagesh, L. C. Gutierrez, K. V. Snyder, J. M. Davies, E. I. Levy, A. H. Siddiqui, Design

and physical properties of 3-dimensional printed models used for neurointervention: A systematic review of the literature. *Neurosurgery* **87**, E445–E453 (2020).

148. C. M. Laurence Klotz, Can high resolution micro-ultrasound replace MRI in the diagnosis of prostate cancer? *Focus* **6**, 419–423 (2020).
